# Genetic and codon usage analyses reveal the evolution of the seoul virus

**DOI:** 10.3389/fgene.2025.1544577

**Published:** 2025-06-12

**Authors:** Yamei Wei, Yanan Cai, Xu Han, Zhanying Han, Yanbo Zhang, Yonggang Xu, Caixiao Jiang, Qi Li

**Affiliations:** ^1^ School of Public Health, Hebei Medical University, Shijiazhuang, Hebei, China; ^2^ Institute for Viral Disease Control and Prevention, Hebei Provincial Centre for Disease Control and Prevention, Shijiazhuang, Hebei, China

**Keywords:** hantavirus, seoul virus, genetic, phylogenetic, codon usage bias, evolution

## Abstract

**Introduction:**

Seoul virus (*Orthohantavirus seoulense*, SEOV), a member of the *Hantaviridae*, causes hemorrhagic fever with renal syndrome (HFRS) through rodent hosts. However, its molecular evolutionary dynamics and codon usage patterns remain poorly understood.

**Methods:**

This study integrated coding sequences from GenBank and previously acquired SEOV strains to systematically analyze genetic evolution and codon usage bias.

**Results:**

It revealed that SEOV evolved seven clades (A-G) with distinct amino acid variation sites and geographic clustering. Recombination events were identified during evolution, alongside purifying and positive selection on specific sites (e.g., codon 259 in the S segment and codon 11 in the M segment). The three viral segments (L, M, and S) exhibited weak codon usage bias, predominantly driven by natural selection, with host adaptation significantly influencing evolutionary trajectories. The S segment demonstrated the strongest pathogenicity due to its closer codon usage alignment with *Homo sapiens* (*H. sapiens)* and *Rattus norvegicus* (*R. norvegicus*), whereas the L segment showed the lowest host adaptation. Divergent codon preferences among clades highlighted adaptive strategies in host-virus interactions.

**Conclusion:**

These findings elucidate the evolutionary mechanisms of SEOV and provide a theoretical foundation for live attenuated vaccine design and region-specific viral control strategies.

## 1 Introduction

Hemorrhagic fever with renal syndrome (HFRS) is an important zoonotic disease caused by hantaviruses (HV). Symptoms vary depending on the viral strain and the individual’s immune response, but commonly include fever, headache, acute renal dysfunction, and hemorrhagic manifestations (Tariq and Kim, 2022; Zhu et al., 2013). Seoul virus (SEOV) is an HV known to cause HFRS by contact with infected rodents, particularly *Rattus norvegicus*. It has a worldwide geographic range and is present in rodent hosts in many countries, including Russia, South Korea, the United States, France, China, and Japan (Guterres et al., 2015; Heyman et al., 2004; Jonsson et al., 2010; Seo et al., 2022; Singh et al., 2022). It seriously threatens human health and economic development.

There are currently no specific, effective antiviral therapies available for HV (Singh et al., 2022). The development of effective antiviral therapies and vaccines for HFRS remains an important area of research. Here, we address this gap by studying SEOV genomic structure through an analysis of codon usage pattern, which should aid in improved vaccine development (Liu et al., 2019).

SEOV (*Orthohantavirus seoulense*) is a segmented RNA virus classified in the class *Bunyaviricetes*, order *Elliovirales*, and family *Hantaviridae* (Kuhn et al., 2024). The genome of the SEOV consists of three segments: L (large), M (medium), and S (small). The L and S segments encode the viral RNA polymerase (RdRp) and nucleocapsid protein (NP), while the M segment encodes the glycoprotein (Gn and Gc) (Plyusnin et al., 1996). Codon usage is the driving force behind viral evolution (Bera et al., 2017). One evolutionary way to improve translation efficiency while maintaining the same sequence of amino acids is through codon degeneration or redundancy (McFeely et al., 2022; Rahman et al., 2018). Frequently used codons are present across many species, a phenomenon termed codon usage bias, which is ubiquitous from prokaryotes to eukaryotes to viruses (Grantham et al., 1980; Parvathy et al., 2022; Patil et al., 2021; Zhao et al., 2016; Zu et al., 2022). Codon selection is influenced by various factors, including recombination, mutational bias, nucleotide content, natural selection, mutational pressure, protein secondary motifs, protein hydrophilicity, hydrophobicity, transcription factors, and the external environment (Martin et al., 2015; Palidwor et al., 2010;Plotkin and Kudla, 2011; Zhao et al., 2021; Zhou et al., 2016). However, mutational pressure and natural selection are considered the major drivers of differences in codon usage between organisms (Plotkin and Kudla, 2011). Different viruses have different patterns of codon usage that result from varied external forces. For example, the patterns of Japanese encephalitis virus (Suresh et al., 2023), severe fever with thrombocytopenia syndrome virus (SFTSV) (Zu et al., 2022), and hantaan virus (HTNV) (Ata et al., 2021) were primarily shaped by natural selection, whereas the patterns of H1N1 (Wong et al., 2010) were primarily driven by mutational pressure. However, codon usage patterns in SEOV are poorly understood.

Here, the SEOV coding sequence was used for codon usage analyses. The analyse provides an understanding of the evolutionary characteristics of SEOV, shedding light on its genetic diversity, adaptive strategies, and potential public health implications.

## 2 Materials and methods

### 2.1 Data collection

All sequences were obtained from GenBank (Release 254.0, accessed December 2022) with complete coding sequences (CDS) of the three segments (L, M, and S) of SEOV. Background information was extracted from GenBank for the three segment sequences, including the strain, host, collection date, and geographic location. To ensure the accuracy of the background information, we verified the sequence information in comparison to published articles. Previously, we obtained the complete sequences of 62 SEOV strains (6 from virus isolates and 56 from tissue samples) from Hebei Province using RT‒PCR combined with NGS (Wei et al., 2023). A total of 550 sequences (L: 108; M: 196; S: 245) were obtained from 297 isolates, including 62 strains reported in previous studies (Wei et al., 2023). Among them, sequences with 100% identity were removed from the study, and a total of 273 isolates (L: 95; M: 166; S: 220) were considered for further analysis in FASTA format (Supplementary Table S1).

### 2.2 Genetic evolution

#### 2.2.1 Recombination analysis

Because recombination strongly affects phylogenetic inference and codon usage, we detected recombination signals using RDP4 for the entire dataset. We selected seven primary exploratory methods for detecting recombination signals included in the recombination detection program (RDP) (Martin et al., 2015). To be considered reliable, a recombination event must satisfy *P* < 0.01. The occurrence of a recombination event in the same sequence also had to be confirmed by at least two algorithms (Su et al., 2019).

#### 2.2.2 Phylogenetic and amino acid-specific mutation site analysis

Recombinant isolates were initially excluded. Multiple sequence alignment and homology were conducted via the Clustal W method implemented by MegAlign Pro (DNASTAR, Inc.). The jModelTest v 2.1.7 software (Posada, 2008), evaluates different evolutionary models and selects GTR + G + I as the best fitting alternative model for all segments of SEOV. Phylogenetics was completed using the maximum likelihood (ML) method in MEGA v 7.0.26 (Kumar et al., 2016), with 1000 bootstrap replicates to assess nodal support. The outgroup used was Hantaan virus (76–118). All analyzed sequences are shown in Supplementary Table S1. The final phylogeny was displayed using FigTree v l.4.4.

A comparative analysis of the three segments at the amino acid and evolutionary branch levels was performed to identify specific mutation sites using the metadata-driven comparative analysis tool (meta-CATS) on the ViPR website (Pickett et al., 2013).

#### 2.2.3 Selection pressure analyses

To assess selective pressure on coding sites, the ratio of nonsynonymous to synonymous nucleotide substitutions per site (dN/dS) was calculated. Several methods have been employed to estimate the dN/dS averages and identify sites of positive selection (Kosakovsky and Frost, 2005; Murrell et al., 2013; 2012). These methods, including SLAC, FEL, FUBAR, and MEME, were accessed via the Datamonkey web server (Weaver et al., 2018). To identify positively selected sites, statistical significance thresholds were set at *P* < 0.05 or posterior probability >0.9 and at least two methods showed statistical significance.

### 2.3 Codon usage pattern

#### 2.3.1 Composition analysis

Nucleotide composition analysis was performed using the CAIcal server (http://genomes.urv.es/CAIcal/) to assess codon usage bias in the SEOV genetic sequence. The analysis focused on examining the frequency and distribution of different nucleotides, including nucleotide content, composition of the 3rd codon position, and composition of GCs at the three codon positions (Puigbo et al., 2008). In addition, the analysis excluded certain codons, such as AUG (Met), UGG (Trp), and the three termination codons UAA, UGA, and UAG, to avoid their influence on the assessment of codon usage bias. We will employ the branch-site model (Model A, NSsites = 2) in PAML 4.9 (Yang, 2007) to identify positively selected sites using likelihood ratio tests (LRT).

#### 2.3.2 Relative synonymous codon usage (RSCU)

The RSCU was calculated using the CAIcal server to characterize the codon usage bias of the SEOV genome, with a value of 1 indicating equal usage of synonymous codons, and values greater than 1.0 or less than 1.0 representing overrepresentation or underrepresentation of synonymous codons, respectively (Sharp et al., 1986). In this particular analysis, thresholds of RSCU >1.6 were considered to indicate overrepresentation, while RSCU <0.6 were considered to indicate underrepresentation (Ata et al., 2021; Wong et al., 2010). Additionally, average RSCU values for *R. norvegicus* (*R. norvegicus*) and *H. sapiens* (*Homo sapiens)* were calculated using the Codon Usage Database (https://www.kazusa.or.jp/codon/).

#### 2.3.3 Trends in codon usage

To identify major trends in SEOV codon usage patterns, a correspondence analysis (COA) approach was used. For every gene in the analysis, 59 dimensions were represented. Each dimension corresponds to an RSCU value for a significant codon. COA calculations were based on RSCU values using CodonW (http://sourceforge.net/projects/codonw).

#### 2.3.4 Codon usage bias

Effective codon counts (ENCs) were analyzed to assess the extent of codon usage bias in different SEOV genes. The values typically range from 20 to 61, with lower values representing higher bias (Smith, 2022; Wright, 1990). A value of ENC close to 45 suggests rational codon usage (Suresh et al., 2023). The ENC values were obtained from CodonW.

#### 2.3.5 Factors contributing to bias

ENC plots were generated to examine the relationship between mutational pressure and codon usage bias. In these plots, the values of GC3s are plotted on the abscissa, and the values of ENC are plotted on the ordinate. The formula typically used for the calculation of the expected ENC values on the basis of the given GC3s is:

$${ENC}^{expected=}2+s+29/\left[s^{2}+{\left(1-s\right)}^{2}\right]$$

By comparing the observations of the genes with the expected ENC curves based on GC3, it is possible to make a preliminary judgment on the extent of the effect of mutational stress on codon usage preferences and to further explore other possible influencing factors.

Parity rule 2 (PR2) analysis calculated the AU bias vs. GC bias through the composition of the 3rd codon position of 4-codon degeneracy amino acids, and used the correspondence to analyze the effects of mutation and selection on the pattern of codon usage. The results of the PR2 analysis indicated that the affected codon was a random mutation (A3 = T3, G3 = C3) or a combination of mutation and selection (A3 ≠ T3, G3 ≠ C3) (Sueoka, 1995; Sueoka, 1999).

Based on SEOV codon usage, neutrality analysis can reveal the extent of mutation and selection. With GC3 as the abscissa and GC12 as the ordinate, the effects were expressed as linear relationships. As the regression slope approaches 1, the effect of directional mutational pressure increases (Sueoka, 1988).

#### 2.3.6 Codon usage adaptation

The codon adaptation index (CAI) compares the RSCU values of SEOV genes to the RSCU values of highly expressed genes in the host species (*H. sapiens* and *R. norvegicus*). A higher CAI indicates a greater similarity between the codon usage pattern of SEOV genes and the reference set of codon usage for highly expressed genes in the host. This suggests that the gene may be highly expressed and under strong selective pressure (Sharp and Li, 1987). The CAI values of SEOV were calculated using the RSCU of *H. sapiens* and *R. norvegicus* as a reference.

The relative codon deviation index (RCDI) assesses how closely the codon usage patterns of each SEOV gene match those found in host genomes (*H. sapiens* and *R. norvegicus*) and tests the degree of deoptimization of the viral genome. Lower RCDI values indicate a high degree of host adaptation, while higher values indicate the expression of certain genes during latency or low levels of viral replication (Mueller et al., 2006; Puigbo et al., 2010). RCDIs for SEOV were calculated using the RCDI/eRCDI online server (http://genomes.urv.es/CAIcal/RCDI/).

The similarity index for codons (SiD) analysis allows an assessment of the extent to which codon usage is influenced by the overall codon usage preferences in the host genome. Higher values indicate codon usage that is more consistent with the preferences of the host genome, and *vice versa* (Zhou et al., 2013). The formula is calculated as follows.

$$R\left(A,B\right)=\frac{\sum_{i=1}^{59}a_{i}^{*}b_{i}}{\sqrt{\sum_{i=1}^{59}a_{i}^{2*}\sum_{i=1}^{59}b_{i}^{2}}}$$

$$D\left(A,B\right)=\frac{1-R\left(A,B\right)}{2}$$

Here, ai and bi represent the RSCU of the 59 synonymous codons in the coding sequence of SEOV and the same codons in the host, respectively. D (A, B) is used to summarize the overall impact of codon usage in the host on SEOV.

## 3 Results

### 3.1 Genetic evolution

#### 3.1.1 Recombination analysis

Our analysis revealed nine recombination events, and these recombination events occurred in all three segments (Table 1). All recombinant strains except Hu02-258/NGS were obtained from China. The recombinant strains were mainly found in *R. norvegicus* (8/9) and not in *H. sapiens* (Supplementary Table S1).

**TABLE 1:** Recombination statistics for the segments of SEOV.

Segment	L				M			S	
Name	HN4	LN04	LN05	Hu02-258/NGS	GZRn110	WuhanMm24	HBT64/2014	RuianRn33	CixiRn76
Minor Parent	Humber	LN03	LN06	80–39	unknown	WuhanRf02	HBT65/2014	unknown	CixiRf56
Major Parent	GZ488	LN06	LN03	SOV/Rn19-5	GZRn98	Tcho	HBT63/2013	CixiRn76	unknown
Begin	3247	6456	6423	5463	598	3145	2209	1036	547
End	4131	757	747	2154	1471	122	3561	1278	1106
RDPRCS	0.739	0.698	0.591	0.502	0.671	0.623	0.619	0.733	0.547
RDP	5.74E-22	9.25E-06	4.36E-05	─	3.65E-06	6.22E-05	─	─	─
GENECONV	3.39E-20	1.42E-06	─	─	2.02E-03	2.92E-02	─	9.70E-03	─
Bootscan	6.44E-23	3.73E-08	4.36E-05	─	2.10E-06	6.11E-05	─	1.13E-04	─
Maxchi	3.46E-08	1.33E-02	9.37E-03	1.02E-03	2.10E-07	3.70E-02	1.37E-02	1.33E-04	2.21E-02
Chimaera	3.08E-09	6.33E-03	7.79E-03	2.76E-02	1.17E-07	─	─	2.75E-03	─
SiSscan	5.04E-13	4.51E-06	4.95E-02	1.35E-04	3.09E-10	─	─	1.23E-07	3.95E-03
3Seq	9.37E-13	2.32E-06	─	─	─	─	1.07E-03	1.12E-03	─

Note: “─“- not significant.

#### 3.1.2 Phylogenetic and amino acid-specific mutation site analysis

**FIGURE 1:**
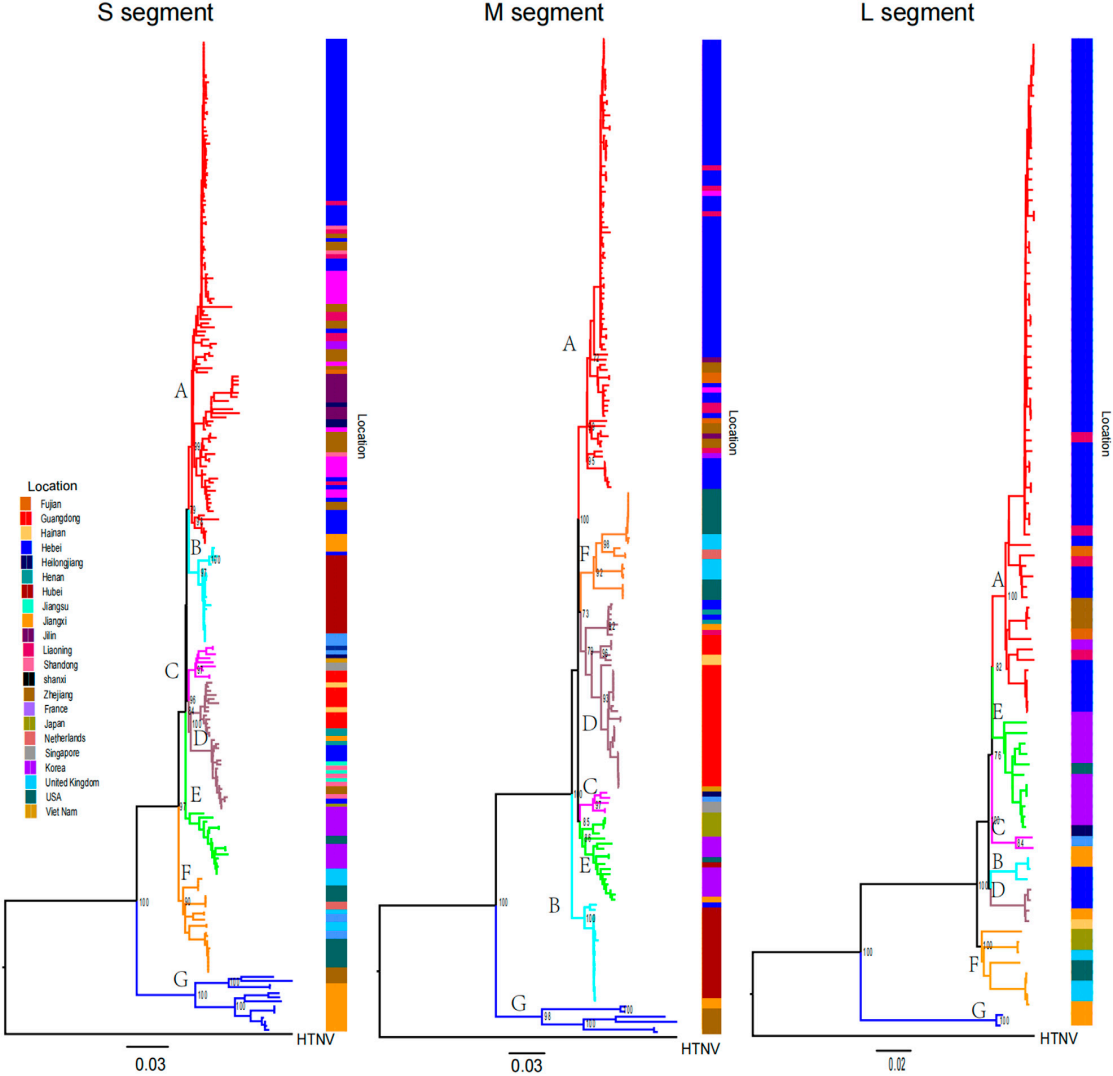
Phylogenetic tree of SEOV reconstructed by ML. Clades A to G and sequences from different locations have different colors. Bootstrap values for major nodes are shown.

Phylogenetic trees were constructed via the ML method. From the phylogenetic tree, we found that SEOV can be divided into seven clades (A-G) and that most of the sequences are within the three segment trees and are clustered primarily by location (Figure 1). The Chinese sequences analyzed in this study were grouped into A, B, D, and G, which represent four genetic lineages with different geographical locations. Clade A consisted of sequences from northern (Hebei, Beijing), northeastern (Liaoning, Heilongjiang, Jilin), and eastern (Shandong, Zhejiang, Fujian) China. Clade B contained sequences from Jiangxi and Wuhan, and Clade D consisted of sequences from southern (Guangdong, Hainan) and eastern (Shandong, Jiangsu, Zhejiang) China. Clades A, B, and D contained a few sequences from outside the main endemic areas, while Clade G consisted of sequences from Jiangxi and Zhejiang. Clades C and E contained sequences from Southeast Asia (Vietnam and Singapore) and East Asia (Korea and Japan), respectively. Clade F contains sequences from the United States and the United Kingdom. However, Group E also included several sequences from the U.S. The 62 sequences we previously obtained are primarily distributed across Group A, B, and D, with Group A dominating (58 sequences, 93.5%), followed by Group B (4 sequences, 6.5%), and Group D (1 sequence, 0.02%).

**FIGURE 2:**
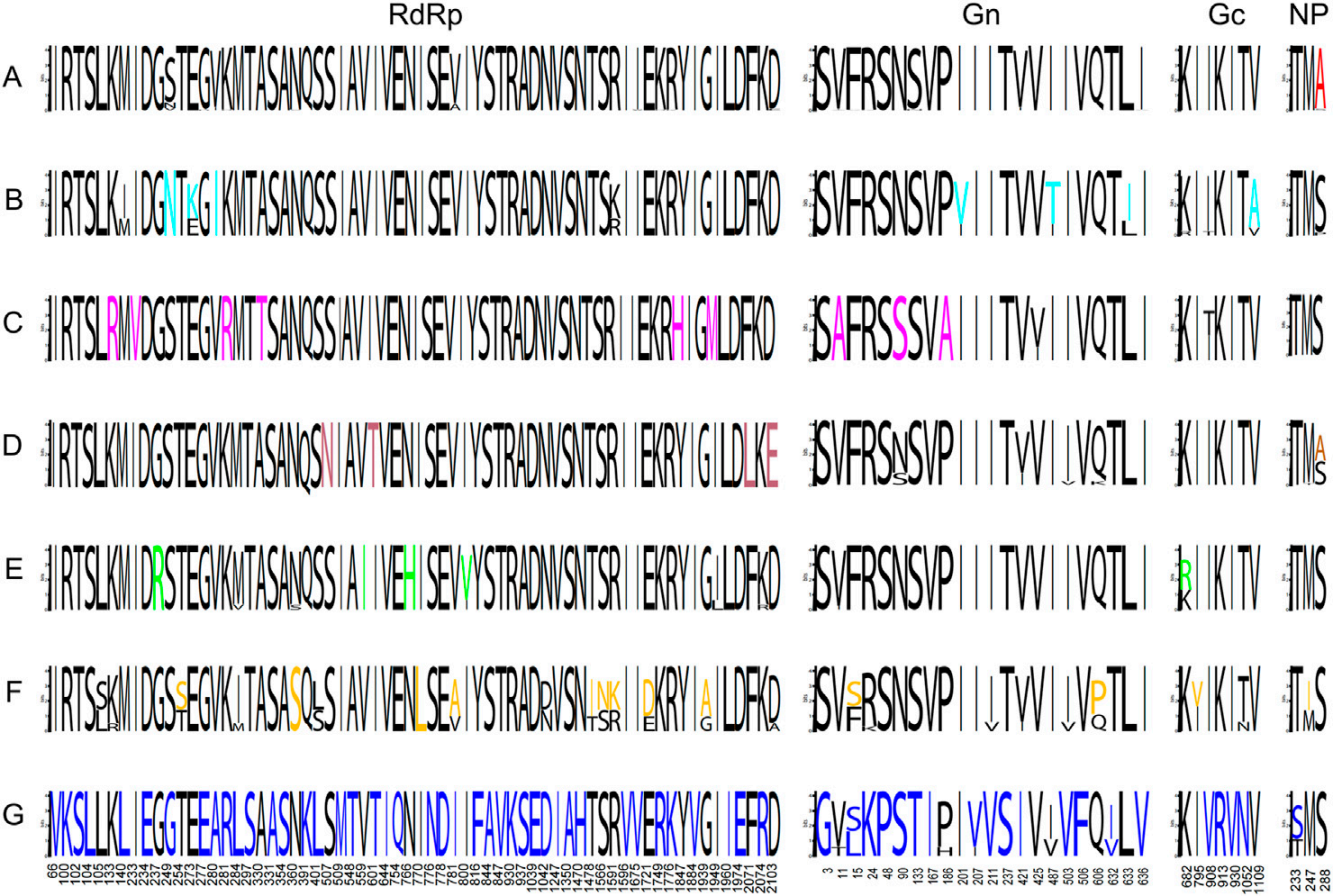
Specific amino acid mutation sites at the evolutionary branch level. The specific amino acid mutation sites in clades A to G have different colors. The numbers below indicate the positions of amino acid mutation sites in their corresponding encoded proteins.

We analyzed specific amino acid mutation sites at the evolutionary branch level, provided that the frequency of the mutation site was greater than 50% in all sequences of the corresponding clade. A total of 96 such sites were found, with 64, 22, 7, and 3 mutation sites in the RdRp, Gn, Gc, and NP genes, respectively (Figure 2). Further analysis revealed that the mutation sites occurred mainly in clade G, with 59 specific amino acid mutation sites (42, 15, 4 and 1 mutation site each of RdRp, Gn, Gc and NP) associated with clade G. The remaining gene clusters had their own specific amino acid mutation sites (Supplementary Tables S2–S5). These mutation sites indicate the molecular characteristics of SEOV sequences in different clusters and can be used as potential molecular markers for SEOV classification.

#### 3.1.3 Selection pressure analyses

By calculating the dN/dS values for the coding regions, positive selection sites and clades were identified. All three segments had dN/dS values well below 1, indicating a tendency toward purifying selection. The S segment exhibited reduced purifying selection compared to the L and M segments (dN/dS = 0.0716 vs. 0.031 and 0.0457, respectively). Two sites with evidence for positive selection were detected at codon 259 (S segment) and codon 11 (M segment) (Table 2). Evidence for episodic diversifying selection of two branches (93HBX11 and R22) was found by BSREL in the phylogeny of the M and S segments.

**TABLE 2:** Positive selection points identified by different methods.

Gene	FUBAR		SLAC			FEL			MEME		
	Num	Site	dN/dS	Num	Site	dN/dS	Num	Site	dN/dS	Num	Site
L	0	—	0.031	0	—	0.031	0	—	0.026	21	115, 198, 245, 249, 333, 409, 446, 541, 773, 810, 890, 1053, 1079, 1126, 1183, 1423, 1495, 1880, 2012, 2014, 2143
M	2	11, 1085	0.0457	1	11	0.0457	1	11	0.0373	21	11, 96, 102, 327, 409, 439, 484, 537, 625, 627, 674, 711, 738, 882, 965, 968, 980, 982, 1022, 1064, 1065
S	0	—	0.0716	0	—	0.0716	1	259	0.0654	8	61, 259, 331, 334, 336, 369, 399, 403

Note: Num: Estimated number of amino acid sites under positive selection; Site: Positions of the candidate sites; Underline: Possible positive selection sites.

To further investigate the positive selection sites, we employed a branch-site model of PAML to detect sites under positive selection. This model allows for the detection of positive selection acting on specific branches of the phylogenetic tree, which is particularly useful for identifying episodic diversifying selection. The results from PAML were consistent with the findings from the SLAC, FEL, FUBAR, and MEME methods, confirming the presence of positive selection at codon 259 (S segment) and codon 11 (M segment) (Supplementary Table S6).

### 3.2 Codon usage pattern

#### 3.2.1 Nucleotide contents of SEOV

First, we calculated the nucleotide composition of SEOV (Supplementary Tables S7–S9). In all three segments, the content of A was significantly higher compared to U, G, or C, and the mean percentage of AU was higher than that of GC (Table 3). For more detailed information on codon composition, the content of the nucleotide at the 3rd codon position was also examined, and the contents of A3 and U3 were greater than those of G3 and C3. The enrichment of A and U nucleotides in SEOV coding sequences is emphasized. An important indicator of base composition bias is the content of GC at each codon position. The content of GC3 was lower than the total content of GC and was the lowest among all codon positions (Table 3). This finding suggested that AU nucleotides occur more frequently at the 3rd codon. The 3rd codon position is critical for understanding synonymous codon usage bias, as mutations at this position often do not alter the encoded amino acid (synonymous mutations). This position is more susceptible to mutational pressure and natural selection, making it a key determinant of codon adaptation to host translation machinery. Therefore, the nucleotide composition influences codon usage in the SEOV coding sequence.

**TABLE 3:** The nucleotide contents of the three SEOV segments are presented as the average and standard deviation.

Nucleotides	Mean ± standard deviation		
	L segment	M segment	S segment
A	32.44 ± 0.10	30.31 ± 0.16	31.36 ± 0.28
U	30.06 ± 0.16	29.61 ± 0.16	22.70 ± 0.32
G	21.17 ± 0.09	21.50 ± 0.16	26.11 ± 0.22
C	16.32 ± 0.09	18.58 ± 0.16	19.83 ± 0.25
A3	43.48 ± 0.43	41.89 ± 0.67	38.72 ± 0.91
U3	46.13 ± 0.48	44.41 ± 0.58	33.38 ± 1.08
G3	23.96 ± 0.45	19.47 ± 0.75	31.90 ± 0.91
C3	15. 73 ± 0.28	21. 82 ± 0.56	22. 73 ± 0.87
AU	62. 51 ± 0.15	59.92 ± 0.25	54. 06 ± 0.32
GC	37.49 ± 0.15	40. 08 ± 0.25	45.94 ± 0.32
GC1	45.90 ± 0.15	44.87 ± 0.25	52.97 ± 0.35
GC2	34.30 ± 0.06	41.65 ± 0.12	40.02 ± 0.17
GC3	32.28 ± 0.43	33.71 ± 0.77	44.84 ± 0.90

#### 3.2.2 RSCU analysis of SEOV

RSCU analysis revealed that the RSCU values of most codons ranged from 0.6 to 1.6, indicating a stable genetic composition of SEOV. Of the 18 most frequently used codons, 17 (10 A, 7 U) were in the L segment, 15 (8 A, 7 U) were in the M segment, and 11 (5 A, 6 U) were in the S segment end in A/U (Table 4). Analysis of codon overrepresentation revealed that almost all overrepresented codons ended in A/U. These results support the view that codon usage in SEOV genes is biased toward A/U end codons.

**TABLE 4:** RSCU patterns for SEOV and its host species.

Amino acid	Codon	L segment	M segment	S segment	*H. sapiens*	*R. norvegicus*
Phe	UUU	1.49	1.48	1.11	0.93	0.83
Phe	UUC	*0.51*	*0.52*	0.89	1.07	1.17
Leu	UUA	**1.66**	1.39	0.86	0.46	0.36
Leu	UUG	1.2	1.27	0.88	0.77	0.76
Leu	CUU	1.41	1.09	1.41	0.79	0.75
Leu	CUC	*0.33*	0.93	0.68	1.17	1.22
Leu	CUA	0.86	0.83	*0.40*	*0.43*	*0.45*
Leu	CUG	*0.54*	*0.50*	**1.78**	**2.37**	**2.46**
Ile	AUU	1.43	1.54	0.98	1.08	0.98
Ile	AUC	0.73	0.71	1.15	1.41	1.57
Ile	AUA	0.84	0.75	0.87	*0.51*	*0.45*
Val	GUU	1.46	**1.72**	0.81	0.73	0.65
Val	GUC	*0.45*	0.94	1.09	0.95	1.02
Val	GUA	1.25	0.64	0.73	*0.47*	*0.45*
Val	GUG	0.84	0.71	1.37	**1.85**	**1.88**
Ser	UCU	1.47	1.13	0.83	1.13	1.12
Ser	UCC	*0.42*	*0.36*	*0.15*	1.31	1.35
Ser	UCA	**2.01**	**2.40**	**2.89**	0.9	0.83
Ser	UCG	*0.21*	*0.06*	*0.22*	*0.33*	*0.33*
Ser	AGU	1.34	1.35	0.98	0.9	0.9
Ser	AGC	*0.55*	0.70	0.93	1.44	1.46
Pro	CCU	**1.77**	1.52	**1.81**	1.15	1.2
Pro	CCC	*0.28*	*0.56*	*0.54*	1.29	1.25
Pro	CCA	**1.72**	**1.72**	1.34	1.11	1.12
Pro	CCG	*0.23*	*0.20*	*0.31*	*0.45*	*0.43*
Thr	ACU	1.17	1.22	0.79	0.99	0.96
Thr	ACC	*0.26*	*0.30*	*0.21*	1.42	1.46
Thr	ACA	**2.4**	**2.30**	**2.88**	1.14	1.13
Thr	ACG	*0.18*	*0.18*	*0.13*	*0.46*	*0.46*
Ala	GCU	1.49	0.94	1.12	1.06	1.14
Ala	GCC	*0.32*	1.02	0.73	1.6	1.57
Ala	GCA	**2.14**	**1.97**	**2.09**	0.91	0.9
Ala	GCG	*0.05*	*0.07*	*0.06*	*0.42*	*0.4*
Tyr	UAU	1.58	1.29	1.53	0.89	0.81
Tyr	UAC	*0.42*	0.71	*0.47*	1.11	1.19
His	CAU	1.47	1.49	**1.68**	0.84	0.78
His	CAC	*0.53*	*0.51*	*0.32*	1.16	1.22
Gln	CAA	1.11	1.06	0.89	*0.53*	*0.49*
Gln	CAG	0.89	0.94	1.11	1.47	1.51
Asn	AAU	1.22	1.27	0.85	0.94	0.82
Asn	AAC	0.78	0.73	1.15	1.06	1.18
Lys	AAA	1.18	1.31	*0.57*	0.87	0.76
Lys	AAG	0.82	0.69	1.43	1.13	1.24
Asp	GAU	1.52	1.41	1.16	0.93	0.86
Asp	GAC	*0.48*	*0.59*	0.84	1.07	1.14
Glu	GAA	1.11	1.36	1.16	0.84	0.79
Glu	GAG	0.89	0.64	0.84	1.16	1.21
Cys	UGU	1.27	1.31	1.26	0.91	0.91
Cys	UGC	0.73	0.69	0.74	1.09	1.09
Arg	CGU	*0.43*	*0.16*	*0.48*	*0.48*	*0.54*
Arg	CGC	*0.19*	*0.02*	0.81	1.1	1.06
Arg	CGA	0.6	0.80	*0.23*	0.65	0.73
Arg	CGG	*0.54*	*0.51*	*0.47*	1.21	1.18
Arg	AGA	**1.93**	**2.70**	**2.11**	1.29	1.21
Arg	AGG	**2.32**	**1.81**	**1.90**	1.27	1.28
Gly	GGU	**1.81**	1.60	0.77	0.65	0.7
Gly	GGC	*0.46*	0.81	*0.31*	1.35	1.34
Gly	GGA	0.96	0.68	1.08	1	1.02
Gly	GGG	0.77	0.92	**1.84**	1	0.95

Note: Codons with RSCU ≥1.6 are shown in bold; the most frequently used codons are underlined; codons with RSCU <1.6 are shown in italic.

**FIGURE 3:**
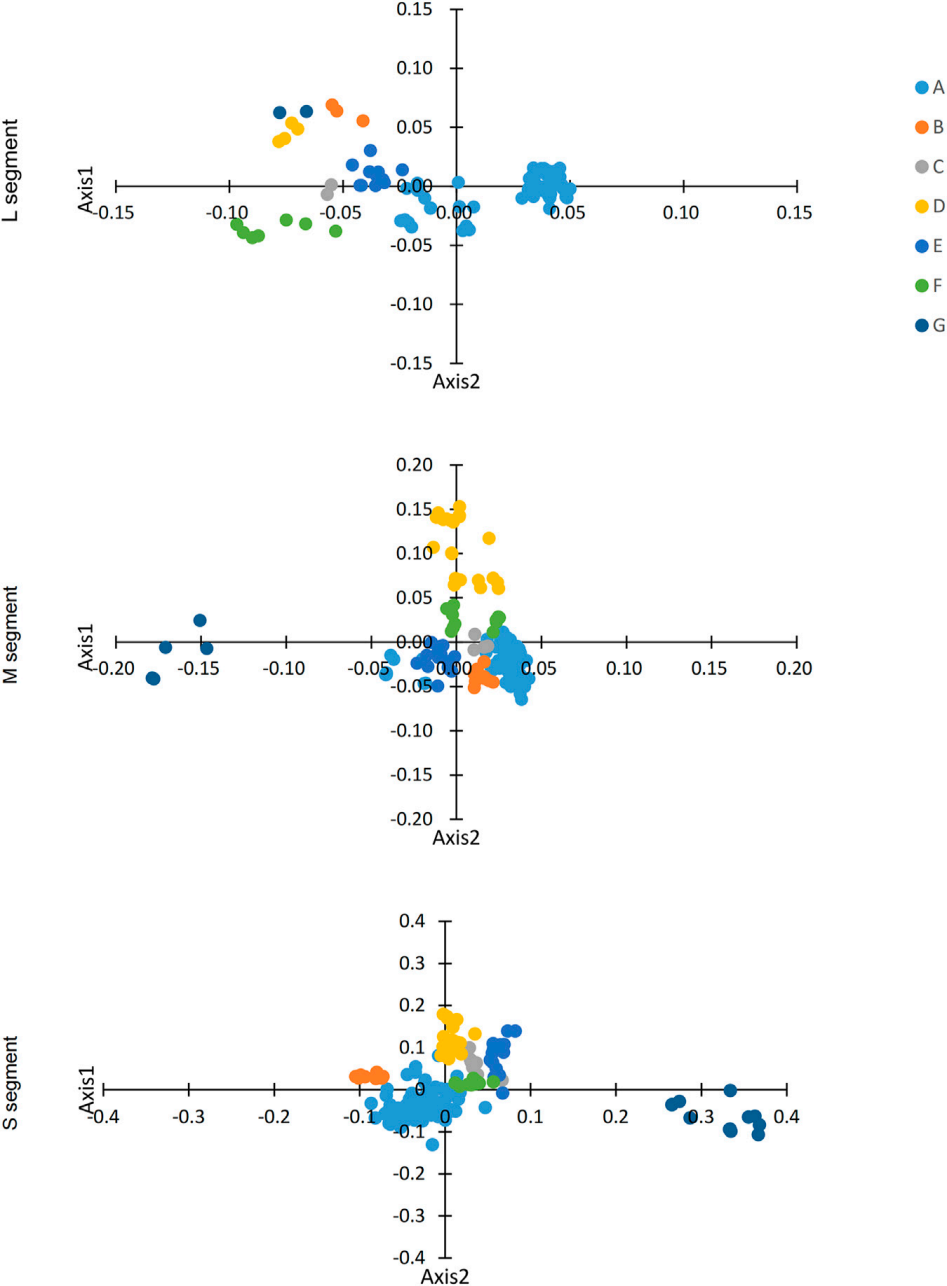
COA analysis of SEOV. The first 2D coordinate was used to plot the codon positions.

The RSCU values of the three codon segments of SEOV were also evaluated in comparison to the RSCU values in other hosts (*H. sapiens* and *R. norvegicus*) (Table 4; Supplementary Figure S1). In the L and M segments, codon usage patterns were not consistent with those of *H. sapiens* or *R. norvegicus*, except for AGG (Arg) in the L segment for *R. norvegicus* and AGA (Arg) in the M segment for *H. sapiens*. However, the codon usage of the S segment was more similar to that of the host than was that of the L and M segments (seven in *H. sapiens* and six in *R. norvegicus*) (Table 4). As shown in Figure 3, the S segment codons (e.g., AGA for Arg, GCA for Ala) exhibited RSCU values closer to *H. sapiens* than other segments, suggesting host adaptation. We performed further RSCU analyses based on the host of isolation by calculating RSCU values for each segment of SEOV strains isolated from *H. sapiens* and *R. norvegicus* and compared them with the corresponding hosts (Supplementary Table S10). Compared with those of the overall sequences, the preferred codons for the S segment in the sequences of the *H. sapiens* isolate were slightly lower (Ile and Val), whereas the preferred codons for the other segments were the same as those in the overall sequence. The preferred codons for the segments in the sequence of the *R. norvegicus* isolate remained consistent with the overall sequence. In conclusion, the S segment codon usage pattern differed least from that of the host codon and was more likely to achieve higher expression in host cells. The clade-specific RSCU analyses were consistent with the overall sequence (Supplementary Tables S11-S13), suggesting that codon usage patterns have limited SEOV evolution to some extent.

#### 3.2.3 Trends in codon usage of SEOV

We identified variation in synonymous codon usage in different SEOV sequences by performing COA analysis on the three segments of SEOV. The proportions of total variation on the first two principal axes are as follows: L: ƒ' 1 = 35.58%, ƒ' 2 = 10.15%; M: ƒ' 1 = 25.32%, ƒ' 2 = 17.35%; S: ƒ' 1 = 27.12%, ƒ' 2 = 14.17%. Notably, the three segments of SEOV were grouped into seven clusters on the major axis (Figure 3). Examination of this variation showed that the clustered form of the strains was consistent with the clustered form generated in the phylogenetic analysis. Specific clustering among clade A was more dispersed than among the other clusters, which may be related to the high diversity of strains in this cluster.

#### 3.2.4 Codon usage bias in SEOV

The mean effective codon counts (ENCs) for the three segments were 47.02 ± 0.32 (L), 47.98 ± 0.54 (M), and 49.19 ± 0.90 (S) (Supplementary Table S14). Notably, all the values are >35, indicating that SEOV has a low bias in codon usage and a conserved composition. Among the three segments of SEOV, the S segment had the highest ENC value (F = 347.611, *P* < 0.001), and the mean ENC values of the different clades varied significantly (L: F = 16.354, *P* < 0.001; M: F = 13.952, *P* < 0.001; S: F = 32.646, *P* < 0.001) (Supplementary Figure S2).

#### 3.2.5 Factors influencing codon usage patterns in SEOV

**FIGURE 4:**
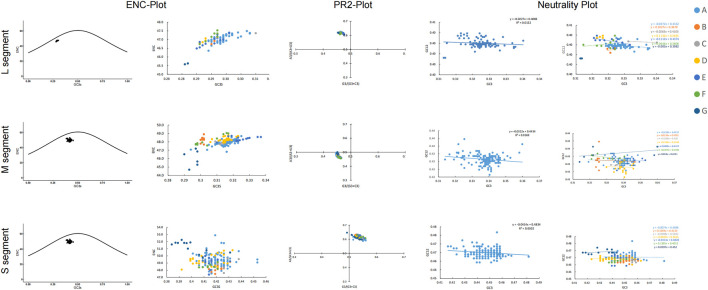
Factors contributing to bias in SEOV codon usage. In the ENC plot, the curve is the expectation curve for each sequence position in the SEOV. PR2 analysis: A value of 0.5 or 0.5 indicates that the effects of mutational pressure and natural selection, respectively, did not cause bias. Neutral plot: Regression plots with slopes closer to 1 indicate greater mutation pressure.

We also used the ENC plot and PR2 analysis to evaluate the influence of mutational pressure on the usage of SEOV codons. In the ENC plot, not all SEOV sequences lie on the expected curve, but all SEOV sequences cluster below it. This indicates that SEOV codon usage is influenced by factors other than mutation (Figure 4). PR2 analysis of purines and pyrimidines in the fourfold degenerate codons showed that most SEOVs were distant from the origin (0.5, 0.5), indicating an unequal nucleotide distribution in the three segments. The above results suggest that natural selection also plays a role in codon usage for SEOV.

Neutrality analysis reveals the extent of the effects of mutational pressure and natural selection on the patterns of codon usage. In the M and S segments, GC12 and GC3 were significantly correlated (M: r = 0.192, P = 0.013; S: r = 0.174, P = 0.011). The linear regression slopes for these two segments were 0.0322 and 0.0414 (Figure 4), indicating direct mutation pressure effects of 3.22% and 4.1%, respectively. Natural selection effects were 96.78% and 95.86% in the M and S segments, respectively. In the L segment, GC12 and GC3 were not significantly correlated (r = 0.114, P = 0.270), and the regression slope was 0.0227, indicating a direct mutational pressure effect of 2.27%. Thus, the influence of natural selection remained dominant (Figure 4). Consistent evidence was also found when examining selection pressure in the SEOV coding region, with the S segment (dN/dS = 0.0716) being less affected by selection pressure (Table 2). Natural selection was dominant, although the extent of its effect differed between the clades.

#### 3.2.6 Codon usage adaptation in SEOV

**FIGURE 5:**
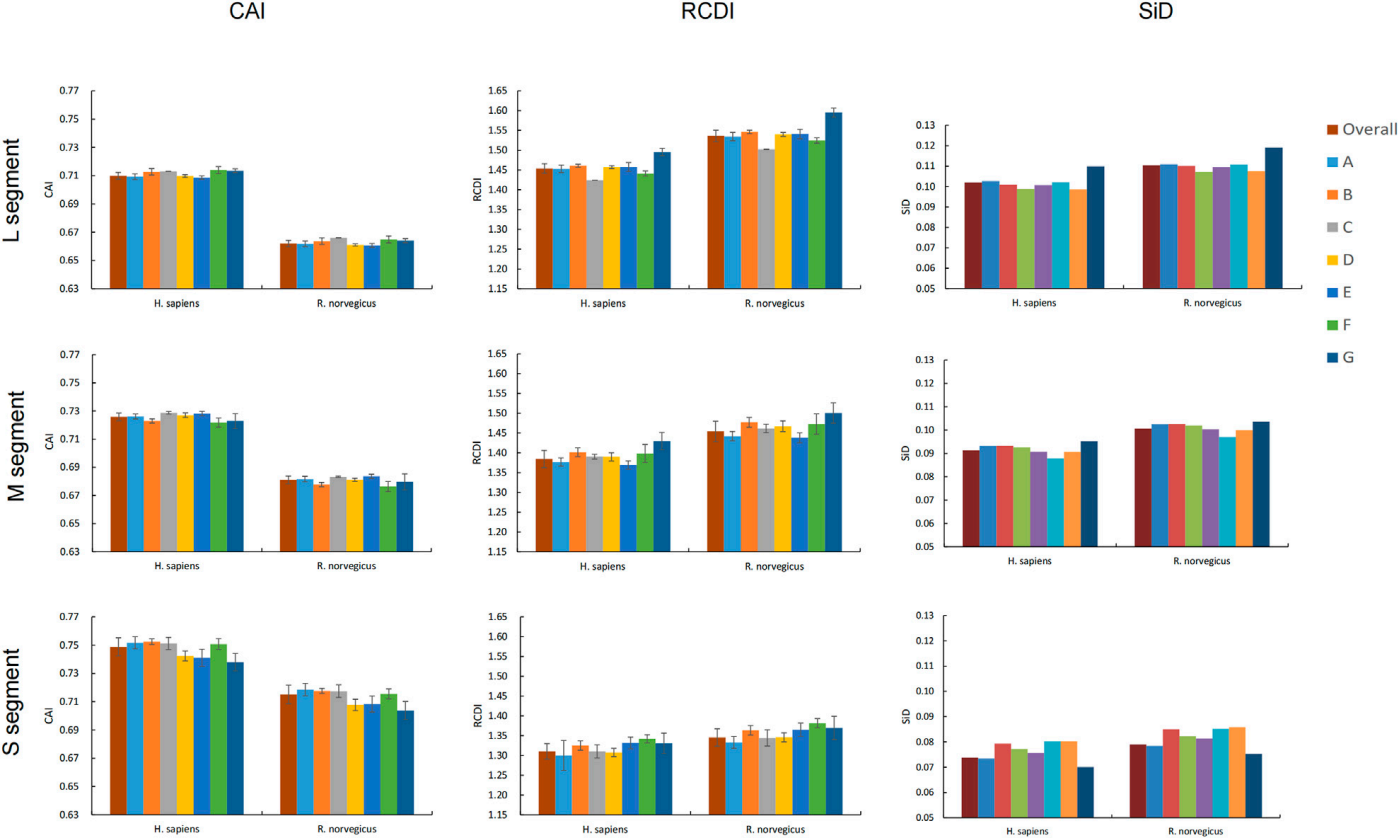
Adaptation of SEOV to the host. Different clades are represented by different colors.

CAI values for all codons were calculated using codon usage in *H. sapiens* and *R. norvegicus* as a reference to assess SEOV adaptation and codon usage optimization in *H. sapiens* and *R. norvegicus*. In each genomic segment, SEOV codon usage adaptation and expression levels were greater in *H. sapiens* than in *R. norvegicus* (Figure 5) (L: F = 20052.877, *P* < 0.001; M: F = 22046.520, *P* < 0.001; S: F = 2918.071, *P* < 0.001). Among the three segments, the CAI values are the highest in the S segment (0.749 ± 0.006 for *H. sapiens* and 0.715 ± 0.007 for *R. norvegicus*) (*H. sapiens*: F = 2522.114, *P* < 0.001; *R. norvegicus*: F = 4648.776, *P* < 0.001). CAI values were also calculated for each clade and were relatively similar for all seven SEOV clades (Figure 5; Supplementary Tables S15-S16). Taken together, these results show that *H. sapiens* has higher levels of adaptation and expression of SEOV codon usage than *R. norvegicus* does, with the S segment having the highest proportion of adaptation to *H. sapiens* and *R. norvegicus* of the three segments.

RCDI analysis measures the extent to which codons in SEOV are deoptimized relative to codon usage patterns in the host genome. The RCDI values of SEOV were greater in *R. norvegicus* than in *H. sapiens* for each genomic segment (Figure 5). The RCDI values were highest in the L segment (1.454 ± 0.012 for *H. sapiens* and 1.536 ± 0.014 for *R. norvegicus*), and the lowest were in the S segment (1.310 ± 0.020 for *H. sapiens* and 1345 ± 0.022 for *R. norvegicus*) (*H. sapiens*: F = 1979.200, *P* < 0.001; *R. norvegicus*: F = 2733.935, *P* < 0.001). We also calculated the RCDI for each segment within each clade compared to *H. sapiens* and *R. norvegicus* and found that clade G had the highest RCDI values for segments L and M, while clade F had the highest RCDI for segment S (Figure 5; Supplementary Tables S17, S18). These results indicate that codon optimization of SEOV is greater in *R. norvegicus* than in *H. sapiens* and that codon optimization of SEOV is clade specific, with noted segment variance, differentially affecting segments L > M > S.

We used SiD analyses to evaluate the influence of SEOV usage patterns in *H. sapiens* and *R. norvegicus*. Clade SiD values were calculated for each segment, and we found that the SiD values of *R. norvegicus* were greater than those of *H. sapiens* across the three SEOV genome segments. This finding suggested that the selection pressure for the usage pattern of the SEOV codon is greater in *R. norvegicus* than in *H. sapiens*. However, the L segment has the greatest impact of the three segments. At the clade level, there were some discrepancies between the results of the clade-specific SiD analysis and overall sequence analysis (Figure 5). Overall, *R. norvegicus* exerted greater selective pressure to originate and evolve SEOV codon usage than did *H. sapiens*, with the L segment exhibiting the most codon usage evolution and the S segment being the least evolved.

## 4 Discussion

In this study, we collected the available CDSs of SEOV from GenBank prior to December 2022 and the genome sequences of 62 SEOV isolates from Hebei Province obtained from our previous study. A comprehensive and systematic evolutionary analysis was performed (Wei et al., 2023).

To characterize the genetic diversity of the strains, we focused on viral genome clustering features and, by phylogenizing the three segments of SEOV, identified seven well supported clades (groups A-G). The results suggest that there is significant genetic diversity in SEOV. COA analysis also revealed that SEOV sequence clustering patterns were consistent with phylogenetic divergence. The genetic diversity of SEOV correlated with geographic location due to the strong geographic aggregation of SEOV. Notably, the majority of recombinant strains analyzed in this study originated from China, which reflects the current availability of SEOV genomic data in public repositories such as GenBank. While this geographic focus provides critical insights into regional viral dynamics, it underscores the need for expanded global surveillance to comprehensively understand SEOV evolution. Future studies incorporating sequences from underrepresented regions (e.g., Europe, Africa, and the Americas) will further validate our findings and elucidate broader evolutionary patterns. Despite this limitation, our identification of clade-specific mutation sites and codon adaptation strategies highlights the role of localized host-pathogen interactions in shaping viral diversity. We found a high number of site-specific mutation sites in the RdRp gene, and combined with selection pressure analysis, we detected a high number of nonsynonymous mutations in the RdRp gene. RdRp is responsible for the transcription and replication of hantaviral genomes, and the mutations identified in this study merit further investigation to determine the relationships between site-specific RdRp gene mutations and viral genome transcription and replication (Kukkonen et al., 2005). Notably, the highest number of site-specific mutation sites was present in clade G, whose SEOV sequences were all from Jiangxi and Zhejiang, with isolation dates ranging from 1999 to 2021. This finding suggested that the space of viral gene variation is more important than the time of variation in SEOV. These findings may be related to the unique geographical location of the region. Furthermore, the S segment, which encodes the nucleocapsid protein, plays a crucial role in viral replication and immune evasion (Plyusnin et al., 1996). The positive selection observed at codon 259 in the S segment may be associated with enhanced viral fitness and pathogenicity, which has significant implications for the development of antiviral therapies and vaccines (Liu et al., 2019). The role of codon selection and translation control is limited by nucleotide biases in RNA viruses, an important determinant of specific codon usage (van Hemert et al., 2016). Nucleotide analysis of SEOV revealed that the contents of A and U were greater than those of G and C, which is consistent with the findings of other hemorrhagic viruses (Ata et al., 2021; Noor et al., 2023; Rahman et al., 2018). All three segments of SEOV displayed weak codon usage bias, as evidenced by ENC values consistently above 35. Comparative ENC analyses across seven evolutionary clades further demonstrated remarkably conserved codon usage patterns among SEOV lineages, suggesting limited divergence in translational optimization mechanisms. These findings align with both overrepresented and underrepresented RSCUs. Such stability mirrors trends observed in diverse RNA viruses, reinforcing the evolutionary constraints shaping their codon adaptation strategies (Bera et al., 2017; Rahman et al., 2018; Wang et al., 2023; Zu et al., 2022). Previous studies have shown that the low codon bias (codon diversity) of RNA viruses helps to reduce host‒virus competition during synthesis, thereby improving viral replication efficiency in host cells (Butt et al., 2016). The three segments of SEOV have different degrees of codon usage bias, with the S segment having the lowest codon bias, suggesting that the S segment has greater viral replication efficiency than the other segments.

The codon bias of SEOV may be driven by both mutational pressure and natural selection, findings that are consistent with previous reports (Suresh et al., 2023; Wang et al., 2023; Wei et al., 2014; Zu et al., 2022). Furthermore, neutral analyses suggest that natural selection is the main factor influencing codon bias in different SEOV clades. SEOV is commonly transmitted from *R. norvegicus* to *H. sapiens* and causes disease in the latter. CAI analysis reflects the effects of natural selection and is often used to assess viral gene adaptation to the host (Carbone et al., 2003; Sharp and Li, 1987). We found that the natural selection of *H. sapiens* and *R. norvegicus* influences SEOV codon usage patterns and that SEOV is highly adaptable to *H. sapiens*, resulting in high replication rates and potentially explaining its pathogenicity in *H. sapiens*. The low adaptability of SEOV to *R. norvegicus* also suggests that SEOV maintains only the low translation rate necessary for protein survival, which is consistent with its pathogenic features in this host, where it often causes chronic, latent infection (Escutenaire and Pastoret, 2000). CAI analysis of SEOV segments also showed that the S segment was the most virulent pathogen and had the highest fitness and gene expression for *H. sapiens* and *R. norvegicus*. Conversely, the L segment was least pathogenic and virulent.

RCDI analysis of individual SEOV segments was consistent with the CAI results and showed that codon deoptimization of individual SEOV segments was greater in *R. norvegicus* than in *H. sapiens* (Ata et al., 2021; Khandia et al., 2019). This finding indicates that the similarities in codon usage between SEOV and *R. norvegicus* are insufficient for efficient viral gene expression, whereas a low RCDI in *H. sapiens* indicates that the codon usage patterns of SEOV and *H. sapiens* are highly similar and adaptive, enabling efficient gene expression at high replication rates. SiD analysis is a valuable tool for exploring how the codon usage pattern of a host organism, including SEOV, impact the evolution of codon usage in viral genomes. Our study suggests that the evolution of codon usage in SEOV is influenced by selective pressures exerted by both *H. sapiens* and *R. norvegicus*. Interestingly, we observed that the S segment of SEOV experiences relatively lower selection pressure than the L and M segments. This finding is consistent with the results of the CAI and RCDI analyses. The results from different codon usage analysis methods consistently indicated that SEOV exhibits a weak codon usage bias, which is predominantly influenced by natural selection. This suggests that SEOV has evolved to optimize its codon usage for efficient replication in its host, particularly in *H. sapiens* (Butt et al., 2016; van Hemert et al., 2016). The low codon bias observed in SEOV may facilitate its adaptation to different host environments, thereby enhancing its ability to cause disease in humans (Wang et al., 2023; Noor et al., 2023).

The findings from this study provide valuable insights into the evolutionary dynamics of SEOV and its adaptation to different hosts. Understanding the codon usage patterns and selection pressures acting on SEOV can inform the design of live attenuated vaccines and other preventive measures (Ata et al., 2021; Luo et al., 2020). By targeting regions under positive selection, it may be possible to develop more effective strategies for controlling SEOV outbreaks and reducing the burden of HFRS in affected populations. Currently, the lack of a complete genome sequence is a major obstacle limiting the understanding of the evolution of SEOV. This is particularly evident in clade analysis, where the limited sample size results in inadequate representativeness, thereby compromising the accuracy of phylogenetic inferences. For instance, Clade C (Southeast Asia) and Clade F (U.S./U.K.) had fewer representatives, which may introduce bias in identifying mutation patterns. Small sample sizes in these clades could amplify the apparent significance of rare mutations or obscure true clade-specific trends. Notably, the majority of sequences were from China (Clades A, B, D, G), potentially overemphasizing mutations linked to regional strains. With the enrichment of SEOV sequences, especially those of the clades, the molecular epidemiological mechanisms of SEOV will be better elucidated.

## 5 Conclusion

This study comprehensively delineates the genetic evolution and codon usage patterns of SEOV. The virus diverged into seven geographically clustered clades, shaped by recombination events and dual selection pressures (purifying and positive selection). Weak codon usage bias across all three segments was predominantly governed by natural selection, with host adaptation playing a pivotal role in viral evolution. The S segment exhibited the highest pathogenicity, attributed to its codon usage optimization for host compatibility, while the L segment displayed the lowest adaptive efficiency. Clade-specific codon preference variations underscored adaptive diversification during host interactions. These insights enhance our understanding of SEOV evolutionary dynamics and offer critical guidance for targeted vaccine development and regional epidemic management. Future studies should incorporate expanded genomic datasets to unravel molecular mechanisms of host-virus interplay and epidemiological linkages.

## Data Availability

The data presented in the study are deposited in the GENBANK repository, accession numbers between OQ739630-OQ739815.
