# Supplementary material for: Genetic and codon usage analyses reveal the evolution of the seoul virus
Source: Front Genet. 2025 Jun 12;16:1544577. doi: 10.3389/fgene.2025.1544577 (PMC12198216; doi:10.3389/fgene.2025.1544577)
Supplement: Supplementary file 1 [file Table1.docx]

Table S1. Sequence information used in this study

| Groups | isolate | L | M | S | County | province | host |
| --- | --- | --- | --- | --- | --- | --- | --- |
| A | Fj372/2013 | KP645196 | KP645197 | KP645198 | China | Fujian | Suncus murinus |
| A | DPRK08 | JX853574 | JX853576 | JX853575 | North Korea |  | Rattus norvegicus |
| A | YaluRiver12 |  |  | HQ611980 | North Korea |  | Rattus norvegicus |
| A | RuianRn180 |  | GU592931 | GU592953 | China | Jiangsu | Rattus norvegicus |
| A | ShenyangRn139 |  |  | GU592951 | China | Liaoning | Rattus norvegicus |
| A | ShenyangRn19 |  |  | GU592950 | China | Liaoning | Rattus norvegicus |
| A | Longwan581 |  | GU592930 | GU592946 | China | Zhejiang | Rattus norvegicus |
| A | RuianRn242 |  | GU592928 | GU592945 | China | Zhejiang | Rattus norvegicus |
| A | XiaotangshanRn7 |  | GU592929 | GU592944 | China | Beijing | Rattus norvegicus |
| A | FeixianRn1 |  |  | GU592942 | China | Shandong | Rattus norvegicus |
| A | GaomiRn9 |  |  | GU592941 | China | Shandong | Rattus norvegicus |
| A | ShuangyangRn470 |  | GU592925 |  | China | Jilin | Rattus norvegicus |
| A | HaixingRn40 |  | GU592924 |  | China | Hebei | Rattus norvegicus |
| A | China |  | EU163437 |  | China | shanxi |  |
| A | Pf26 |  |  | AY006465^#^ | China | Heilongjiang |  |
| A | ZT71 | EF190551 | EF117248 | AY750171 | China | Zhejiang | Rattus norvegicus |
| A | ZT10 | EF581094 | DQ159911 | AY766368 | China | Zhejiang | Microtus fortis |
| A | RuianRr57 |  |  | FJ803217 | China | Zhejiang | Rattus rattus |
| A | RuianRn23 |  |  | FJ803214 | China | Zhejiang | Rattus norvegicus |
| A | OuhaiRf35 |  |  | FJ803208 | China | Zhejiang | Rattus flavipectus |
| A | CixiRn76 |  |  | FJ803206^$^ | China | Zhejiang | Rattus norvegicus |
| A | CixiRn21 |  |  | FJ803205 | China | Zhejiang | Rattus norvegicus |
| A | CixiRf56 |  |  | FJ803202 | China | Zhejiang | Rattus flavipectus |
| A | RuianRn76 |  |  | FJ803216 | China | Zhejiang | Rattus norvegicus |
| A | RuianRn33 |  |  | FJ803215^$^ | China | Zhejiang | Rattus norvegicus |
| A | RuianRf74 |  |  | FJ803213 | China | Zhejiang | Rattus flavipectus |
| A | OuhaiRn251 |  |  | FJ803212 | China | Zhejiang | Rattus norvegicus |
| A | OuhaiRn189 |  |  | FJ803211 | China | Zhejiang | Rattus norvegicus |
| A | OuhaiRn146 |  |  | FJ803210 | China | Zhejiang | Rattus norvegicus |
| A | CixiRn169 |  |  | FJ803207 | China | Zhejiang | Rattus norvegicus |
| A | CixiRf23 |  |  | FJ803201 | China | Zhejiang | Rattus flavipectus |
| A | HBT-3 |  | KM233661 |  | China | hebei | Rattus norvegicus |
| A | HBT50 |  | KM233660 |  | China | hebei | Rattus norvegicus |
| A | HBT49 |  | KM233659 |  | China | hebei | Rattus norvegicus |
| A | HBT14 |  | KM233658 |  | China | hebei | Rattus norvegicus |
| A | HBQ43 |  | KM233657 |  | China | hebei | Rattus norvegicus |
| A | HBQ20 |  | KM233656 |  | China | hebei | Rattus norvegicus |
| A | HBQ17 |  | KM233655 |  | China | hebei | Mus musculus |
| A | HBQ7 |  | KM233654 |  | China | hebei | Rattus norvegicus |
| A | HBQ5 |  | KM233653 |  | China | hebei | Rattus norvegicus |
| A | Taonan420 |  |  | KF745951 | China | jilin | Mus musculus |
| A | Taonan52 |  |  | KF745950 | China | jilin | Mus musculus |
| A | Gongzhuling415 |  |  | KF745949 | China | jilin | Apodemus agrarius |
| A | Gongzhuling147 |  |  | KF745948 | China | jilin | Apodemus agrarius |
| A | Gongzhuling108 |  |  | KF745947 | China | jilin | Rattus norvegicus |
| A | Gongzhuling97 |  |  | KF745946 | China | jilin | Rattus norvegicus |
| A | Gongzhuling85 |  |  | KF745945 | China | jilin | Rattus norvegicus |
| A | Gongzhuling58 |  |  | KF745944 | China | jilin | Rattus norvegicus |
| A | Gongzhuling45 |  |  | KF745943 | China | jilin | Rattus norvegicus |
| A | Gongzhuling42 |  |  | KF745942 | China | jilin | Rattus norvegicus |
| A | HeB38 |  | JN377554 | JN377553 | China | hebei | Microtus fortis |
| A | SC106 |  |  | GU361893 | China | heilongjiang | Rattus norvegicus |
| A | zy27 |  |  | AF406965^#^ | China | heilongjiang |  |
| A | HuBJ20 |  |  | GQ279394 | China | beijing | Homo sapiens |
| A | Rn-DH27 |  |  | GQ279393 | China | beijing | Rattus norvegicus |
| A | Rn-HD11 |  |  | GQ279392 | China | beijing | Rattus norvegicus |
| A | HuBJ3 |  |  | GQ279391 | China | beijing | Homo sapiens |
| A | HuBJ15 |  |  | GQ279390 | China | beijing | Homo sapiens |
| A | HuBJ19 |  |  | GQ279389 | China | beijing | Homo sapiens |
| A | Rn-SHY17 |  |  | GQ279388 | China | beijing | Rattus norvegicus |
| A | Rn-YUE12 |  |  | GQ279387 | China | beijing | Rattus norvegicus |
| A | Rn-DC8 |  |  | GQ279386 | China | beijing | Rattus norvegicus |
| A | SD201 |  |  | GQ279385 | China | shandong | Rattus norvegicus |
| A | HuBJ9 |  |  | GQ279384 | China | beijing | Homo sapiens |
| A | Rn-M11 |  |  | GQ279383 | China | beijing | Rattus norvegicus |
| A | Rn-CP7 |  |  | GQ279382 | China | beijing | Rattus norvegicus |
| A | HuBJ7 |  |  | GQ279381 | China | beijing | Homo sapiens |
| A | HuBJ16 |  |  | GQ279380 | China | beijing | Homo sapiens |
| A | HuBJ22 |  |  | GQ279379 | China | beijing | Homo sapiens |
| A | BjHD01 |  | DQ133505 | AY627049 | China | beijing | Rattus norvegicus |
| A | FJ36 | MW449191^#^ | MW449192 | MW449193^#^ | China | Fujian | Rattus norvegicus |
| A | FJ35 | MW449188 | MW449189 | MW449190 | China | Fujian | Rattus norvegicus |
| A | LN06 | MT711945 | MT711951 | MT711957 | China | Liaoning | Rattus norvegicus |
| A | LN05 | MT711944^$^ | MT711950 | MT711956 | China | Liaoning | Rattus norvegicus |
| A | LN04 | MT711943^$^ | MT711949 | MT711955 | China | Liaoning | Rattus norvegicus |
| A | LN03 | MT711942 | MT711948 | MT711954 | China | Liaoning | Rattus norvegicus |
| A | LN02 | MT711941 | MT711947 | MT711953 | China | Liaoning | Rattus norvegicus |
| A | LN01 | MT711940 | MT711946 | MT711952 | China | Liaoning | Rattus norvegicus |
| A | Z37 | AF285266 | AF187081 | AF187082 | China | Zhejiang | Rattus norvegicus |
| A | JUN5-14 |  |  | DQ217791 | China | Shandong | Rattus norvegicus |
| A | 93HBQ3* | OQ739631 | OQ739693 | OQ739754 | China | Hebei | Rattus norvegicus |
| A | 93HBQ4* | OQ739632 | OQ739694 | OQ739756 | China | Hebei | Striped Hamster |
| A | 93HBX10* | OQ739633 | OQ739695 | OQ739757 | China | Hebei | Rattus norvegicus |
| A | 93HBX11* | OQ739634 | OQ739696 | OQ739758 | China | Hebei | Rattus norvegicus |
| A | 93HBX12* | OQ739635 | OQ739697 | OQ739759 | China | Hebei | Rattus norvegicus |
| A | 93HBJ20* | OQ739630 | OQ739692 | OQ739755 | China | Hebei | Rattus norvegicus |
| A | HBCZ88/1999* | OQ739651 | OQ739713 | OQ739775 | China | Hebei | Rattus norvegicus |
| A | HBQ72/2000* | OQ739675 | OQ739737 | OQ739799 | China | Hebei | Rattus norvegicus |
| A | HBQ73/2000* | OQ739676 | OQ739738 | OQ739800 | China | Hebei | Rattus norvegicus |
| A | HBQ74/2000* | OQ739677 | OQ739739 | OQ739801 | China | Hebei | Rattus norvegicus |
| A | HBQ62/2001* | OQ739670 | OQ739732 | OQ739794 | China | Hebei | Rattus norvegicus |
| A | HBQ63/2001* | OQ739671 | OQ739733 | OQ739795 | China | Hebei | Rattus norvegicus |
| A | HBCD52/2002* | OQ739647 | OQ739709 | OQ739771 | China | Hebei | Rattus norvegicus |
| A | HBCD56/2002* | OQ739650 | OQ739712 | OQ739774 | China | Hebei | Rattus norvegicus |
| A | HBQ49/2004* | OQ739664 | OQ739726 | OQ739788 | China | Hebei | Rattus norvegicus |
| A | HBL3/2005* | OQ739653 | OQ739714 | OQ739777 | China | Hebei | Rattus norvegicus |
| A | HBL131/2007* | OQ739654 | OQ739716 | OQ739778 | China | Hebei | Rattus norvegicus |
| A | HBQ65/2009* | OQ739674 | OQ739736 | OQ739798 | China | Hebei | Rattus norvegicus |
| A | HBQ24/2011* | OQ739662 | OQ739724 | OQ739786 | China | Hebei | Rattus norvegicus |
| A | HBQ4/2012* | OQ739658 | OQ739720 | OQ739782 | China | Hebei | Rattus norvegicus |
| A | HBQ7/2012* | OQ739660 | OQ739722 | OQ739784 | China | Hebei | Rattus norvegicus |
| A | HBT41/2012* | OQ739683 | OQ739745 | OQ739807 | China | Hebei | Rattus norvegicus |
| A | HBT43/2012* | OQ739684 | OQ739746 | OQ739808 | China | Hebei | Rattus norvegicus |
| A | HBT52/2013* | OQ739685 | OQ739747 | OQ739809 | China | Hebei | Rattus norvegicus |
| A | HBT60/2013* | OQ739686 | OQ739748 | OQ739810 | China | Hebei | Rattus norvegicus |
| A | HBT63/2013* | OQ739689 | OQ739751 | OQ739813 | China | Hebei | Rattus norvegicus |
| A | HBT61/2014* | OQ739687 | OQ739749^#^ | OQ739811 | China | Hebei | Rattus norvegicus |
| A | HBT62/2014* | OQ739688 | OQ739750 | OQ739812 | China | Hebei | Rattus norvegicus |
| A | HBT64/2014* | OQ739690 | OQ739752 | OQ739814 | China | Hebei | Rattus norvegicus |
| A | HBT65/2014* | OQ739691^#^ | OQ739753 | OQ739815^#^ | China | Hebei | Rattus norvegicus |
| A | HBQ1/2015* | OQ739655 | OQ739717 | OQ739779 | China | Hebei | Rattus norvegicus |
| A | HBQ2/2015* | OQ739656 | OQ739718 | OQ739780 | China | Hebei | Rattus norvegicus |
| A | HBQ5/2015* | OQ739659 | OQ739721 | OQ739783 | China | Hebei | Rattus norvegicus |
| A | HBT6/2015* | OQ739681 | OQ739743 | OQ739805 | China | Hebei | Rattus norvegicus |
| A | HBT4/2016* | OQ739679 | OQ739741^#^ | OQ739803^#^ | China | Hebei | Rattus norvegicus |
| A | HBT5/2016* | OQ739680 | OQ739742 | OQ739804 | China | Hebei | Rattus norvegicus |
| A | HBT7/2016* | OQ739682 | OQ739744 | OQ739806 | China | Hebei | Rattus norvegicus |
| A | HBCD1/2017* | OQ739638 | OQ739700 | OQ739762 | China | Hebei | Rattus norvegicus |
| A | HBCD3/2017* | OQ739640 | OQ739702 | OQ739764 | China | Hebei | Rattus norvegicus |
| A | HBCD4/2017* | OQ739641 | OQ739703 | OQ739765 | China | Hebei | Rattus norvegicus |
| A | HBCD6/2017* | OQ739642 | OQ739704 | OQ739766 | China | Hebei | Rattus norvegicus |
| A | HBCD8/2017* | OQ739643 | OQ739705 | OQ739767 | China | Hebei | Rattus norvegicus |
| A | HBCD9/2017* | OQ739644 | OQ739706 | OQ739768 | China | Hebei | Rattus norvegicus |
| A | HBCD1/2018* | OQ739639 | OQ739701^#^ | OQ739763^#^ | China | Hebei | Rattus norvegicus |
| A | HBQ15/2018* | OQ739661 | OQ739723 | OQ739785 | China | Hebei | Rattus norvegicus |
| A | HBCD13/2019* | OQ739645 | OQ739707 | OQ739769 | China | Hebei | Rattus norvegicus |
| A | HBCD44/2021* | OQ739646 | OQ739708 | OQ739770 | China | Hebei | Rattus norvegicus |
| A | HBQ47/2021* | OQ739663 | OQ739725 | OQ739787 | China | Hebei | Rattus norvegicus |
| A | HBQ50/2021* | OQ739665 | OQ739727 | OQ739789 | China | Hebei | Rattus norvegicus |
| A | HBH51/2021* | OQ739652 | OQ739715 | OQ739776 | China | Hebei | Rattus norvegicus |
| A | HBCD55/2021* | OQ739649 | OQ739711 | OQ739773 | China | Hebei | Rattus norvegicus |
| A | HBQ57/2021* | OQ739666 | OQ739728 | OQ739790 | China | Hebei | Rattus norvegicus |
| A | HBQ58/2021* | OQ739667 | OQ739729 | OQ739791 | China | Hebei | Rattus norvegicus |
| A | HBQ59/2021* | OQ739668^#^ | OQ739730 | OQ739792 | China | Hebei | Rattus norvegicus |
| A | HBQ60/2021* | OQ739669 | OQ739731 | OQ739793 | China | Hebei | Rattus norvegicus |
| A | HBQ63/2021* | OQ739672 | OQ739734 | OQ739796 | China | Hebei | Rattus norvegicus |
| A | HBQ64/2022* | OQ739673 | OQ739735 | OQ739797 | China | Hebei | Rattus norvegicus |
| B | JiangxiXinjianRn-07-2011 | KP900346 | KP859513 | KP859511 | China | jiangxi | Rattus norvegicus |
| B | JiangxiXinjianRn-09-2011 | MZ504243 | KP859514^#^ | KP859512 | China | jiangxi | Rattus norvegicus |
| B | WuhanRn98 |  | JQ665904 | JQ665928 | China | hubei | Rattus norvegicus |
| B | WuhanRn75 |  | JQ665903^#^ | JQ665927 | China | hubei | Rattus norvegicus |
| B | WuhanRn67u |  | JQ665902 | JQ665926 | China | hubei | Rattus norvegicus |
| B | WuhanRn63 |  | JQ665901^#^ | JQ665925 | China | hubei | Rattus norvegicus |
| B | WuhanRn58 |  | JQ665900 | JQ665924^#^ | China | hubei | Rattus norvegicus |
| B | WuhanRn57 |  | JQ665899 | JQ665923^#^ | China | hubei | Rattus norvegicus |
| B | WuhanRn53 |  | JQ665898 | JQ665922 | China | hubei | Rattus norvegicus |
| B | WuhanRn25 |  | JQ665897 | JQ665921 | China | hubei | Rattus norvegicus |
| B | WuhanRn10 |  | JQ665896^#^ | JQ665920 | China | hubei | Rattus norvegicus |
| B | WuhanRf49 |  | JQ665895 | JQ665919 | China | hubei | Rattus flavipectus |
| B | WuhanRf33 |  | JQ665894^#^ | JQ665918 | China | hubei | Rattus flavipectus |
| B | WuhanRf18 |  | JQ665893^#^ | JQ665917 | China | hubei | Rattus flavipectus |
| B | WuhanRf12 |  | JQ665892 | JQ665916 | China | hubei | Rattus flavipectus |
| B | WuhanRf11 |  | JQ665891# | JQ665915# | China | hubei | Rattus flavipectus |
| B | WuhanRf08 |  | JQ665890 | JQ665914 | China | hubei | Rattus flavipectus |
| B | WuhanRf07 |  | JQ665889^#^ | JQ665913 | China | hubei | Rattus flavipectus |
| B | WuhanRf02 |  | JQ665888 | JQ665912 | China | hubei | Rattus flavipectus |
| B | WuhanMm24 |  | JQ665887^$^ | JQ665911 | China | hubei | Mus musculus |
| B | WuhanMm13 |  | JQ665886 | JQ665910 | China | hubei | Mus musculus |
| B | XJ5/2011 |  |  | KY807170 | China | Jiangxi | Rattus norvegicus |
| B | XJ2/2011 |  |  | KY807169 | China | Jiangxi | Rattus norvegicus |
| B | HBCD55/2002* | OQ739648 | OQ739710 | OQ739772 | China | Hebei | Rattus norvegicus |
| C | LYON/Rn/FRA/2013/LYO852 | KF387723 | KF387724 | KF387725 | France |  | Rattus norvegicus |
| C | REPLONGES/Hu/FRA/2012/12-0882 |  |  | KC902522 | France |  | Homo sapiens |
| C | SEO/Belgium/Rn895/2005 |  |  | JQ898106 | Belgium |  | Rattus norvegicus |
| C | Singapore/06(RN46) |  | GQ274943 | GQ274945 | Singapore |  | Rattus norvegicus |
| C | Singapore/06(RN41) |  | GQ274942^#^ | GQ274944^#^ | Singapore |  | Rattus norvegicus |
| C | 5CSG |  | AB618130 |  | Vietnam:Saigon harbor | | Rattus norvegicus |
| C | CSG5 |  |  | AB618112 | Vietnam:Saigon harbor | | Rattus norvegicus |
| C | MANTENAY-MONTLIN/Rn/FRA/2015/2015.00179 | | | KX064273 | France |  | Rattus norvegicus |
| C | MANTENAY-MONTLIN/Rn/FRA/2015/2015.00173 | | | KX064272^#^ | France |  | Rattus norvegicus |
| C | DN2 | KX289952 | KX289953 | KX289954 | China | Heilongjiang | Rattus norvegicus |
| D | HuludaoRn101 |  | GU592923 | GU592952# | China | Liaoning | Rattus norvegicus |
| D | QixianRn10 |  |  | GU592949 | China | Henan | Rattus norvegicus |
| D | GuangzhouRn36 |  |  | GU592948 | China | Guangzhou | Rattus norvegicus |
| D | JiningCt13 |  |  | GU592940 | China | Shandong | Cricetulus triton |
| D | QingdaoMm15 |  |  | GU592939 | China | Shandong | Mus musculus |
| D | GaomiRn47 |  |  | GU592938 | China | Shandong | Rattus norvegicus |
| D | JinanRn1 |  |  | GU592937 | China | Shandong | Rattus norvegicus |
| D | HebeiRn9 |  |  | GU592936 | China | Hebei | Rattus norvegicus |
| D | HebeiMm7 |  |  | GU592935 | China | Hebei | Mus musculus |
| D | GanyuRn187 |  |  | GU592934 | China | Jiangsu | Rattus norvegicus |
| D | GanyuRn137 |  |  | GU592933 | China | Jiangsu | Rattus norvegicus |
| D | GanyuRn66 |  |  | GU592932 | China | Jiangsu | Rattus norvegicus |
| D | Hb8610 |  |  | AF288643 | China | shanxi | Rattus norvegicus |
| D | L99 | AF288297 | AF288298 | AF288299 | China |  | Rattus loseda |
| D | R22 |  | S68035 | AF488707 | China |  | Rattus norvegicus |
| D | GZ488 |  |  | MZ031964 | China | Guangdong | Rattus norvegicus |
| D | YN45 |  |  | MZ031963 | China | Guangdong | Rattus norvegicus |
| D | XM47 |  |  | MZ031962 | China | Guangdong | Rattus norvegicus |
| D | GZ45 |  |  | MZ031961 | China | Guangdong | Rattus norvegicus |
| D | MM23 |  |  | MZ031960 | China | Guangdong | Rattus norvegicus |
| D | GZ473 |  |  | MZ031959 | China | Guangdong | Rattus norvegicus |
| D | GZ325 |  |  | MZ031958 | China | Guangdong | Rattus norvegicus |
| D | GZ15 |  |  | MZ031957 | China | Guangdong | Rattus norvegicus |
| D | YY27 |  | MZ031947 | MZ031956 | China | Guangdong | Rattus tanezumi |
| D | GZRn148 |  | MN022843 |  | China | Guangdong | Rattus norvegicus |
| D | GZRn134 |  | MN022842 |  | China | Guangdong | Rattus norvegicus |
| D | GZRn133 |  | MN022841 |  | China | Guangdong | Rattus norvegicus |
| D | GZRn127 |  | MN022840 |  | China | Guangdong | Rattus norvegicus |
| D | GZRn110 |  | MN022839^$^ |  | China | Guangdong | Rattus norvegicus |
| D | GZRn107 |  | MN022838 |  | China | Guangdong | Rattus norvegicus |
| D | GZRn104 |  | MN022837 |  | China | Guangdong | Rattus norvegicus |
| D | GZRn100 |  | MN022836 |  | China | Guangdong | Rattus norvegicus |
| D | GZRn98 |  | MN022835 |  | China | Guangdong | Rattus norvegicus |
| D | GZRn96 |  | MN022834 |  | China | Guangdong | Rattus norvegicus |
| D | GZRn95 |  | MN022833^#^ |  | China | Guangdong | Rattus norvegicus |
| D | GZRn93 |  | MN022832^#^ |  | China | Guangdong | Rattus norvegicus |
| D | GZRn92 |  | MN022831 |  | China | Guangdong | Rattus norvegicus |
| D | GZRn84 |  | MN022830 |  | China | Guangdong | Rattus norvegicus |
| D | GZRn83 |  | MN022829# |  | China | Guangdong | Rattus norvegicus |
| D | GZRn77 |  | MN022828 |  | China | Guangdong | Rattus norvegicus |
| D | GZRn76 |  | MN022827 |  | China | Guangdong | Rattus norvegicus |
| D | GZRn74 |  | MN022826 |  | China | Guangdong | Rattus norvegicus |
| D | GZRn73 |  | MN022825 |  | China | Guangdong | Rattus norvegicus |
| D | GZRn60 |  | MN022824 |  | China | Guangdong | Rattus norvegicus |
| D | GZRn55 |  | MN022823^#^ |  | China | Guangdong | Rattus norvegicus |
| D | GZRn54 |  | MN022822 |  | China | Guangdong | Rattus norvegicus |
| D | GZRn53 |  | MN022821 |  | China | Guangdong | Rattus norvegicus |
| D | GZRn51 |  | MN022820 |  | China | Guangdong | Rattus norvegicus |
| D | GZRn44 |  | MN022819^#^ |  | China | Guangdong | Rattus norvegicus |
| D | GZRn42 |  | MN022818^#^ |  | China | Guangdong | Rattus norvegicus |
| D | GZRn41 |  | MN022817^#^ |  | China | Guangdong | Rattus norvegicus |
| D | GZRn36 |  | MN022816 |  | China | Guangdong | Rattus norvegicus |
| D | SZ148 |  |  | OM049762 | China | Guangdong | Rattus norvegicus |
| D | SZ54 |  |  | OM049760 | China | Guangdong | Rattus norvegicus |
| D | HN4 | MZ670780^$^ | MZ670781 | MZ670782 | China | Hainan | Rattus norvegicus |
| D | HN1 | MZ670777 | MZ670778 | MZ670779 | China | Hainan | Rattus norvegicus |
| D | HB55 |  | AF035832 |  | China | Hubei | Homo sapiens |
| D | HBQ75/2000* | OQ739678 | OQ739740 | OQ739802 | China | Hebei | Rattus norvegicus |
| D | HBB35/2002* | OQ739636 | OQ739698 | OQ739760 | China | Hebei | Rattus norvegicus |
| D | HBB36/2002* | OQ739637 | OQ739699 | OQ739761 | China | Hebei | Rattus norvegicus |
| E | 80-39 | NC_005238 | NC_005237 | NC_005236 | North Korea |  | Rattus norvegicus |
| E | Tchoupitoulas/POR | KU204958 | KU204959 | KU204960 | USA |  | Rattus norvegicus |
| E | YZG-Changchun |  |  | EF536376 | China | Jilin | Mus norvegicus albus |
| E | KI-88-15 |  | D17594 |  | Japan |  | Rattus norvegicus |
| E | KI-85-1 |  | D17593 |  | Japan |  | Rattus norvegicus |
| E | KI-83-262 |  | D17592 |  | Japan |  | Rattus norvegicus |
| E | B-1 |  | X53861 |  |  |  | Rattus norvegicus |
| E | Rn 10-145 |  | JF693885 | JF693884 | South Korea |  | Rattus norvegicus |
| E | Rn 10-134 |  | JF693883^#^ | F693882^#^ | South Korea |  | Rattus norvegicus |
| E | SOV/Rn19-5 | OK746250 | OK746252 | OK746254 | South Korea |  | Rattus norvegicus |
| E | SOV/Rn18-1 | OK746249 | OK746251 | OK746253 | South Korea |  | Rattus norvegicus |
| E | Hu02-529/NGS |  |  | MF149956 | South Korea |  | Homo sapiens |
| E | Hu02-294/NGS |  |  | MF149955 | South Korea |  | Homo sapiens |
| E | Hu02-258/NGS | MF149951^$^ | MF149946 | MF149954 | South Korea |  | Homo sapiens |
| E | Rn11-53/NGS | MF149941 | MF149945 | MF149950 | South Korea |  | Rattus norvegicus |
| E | Rn11-44/NGS | MF149940 | MF149944 | MF149949 | South Korea |  | Rattus norvegicus |
| E | Rn10-145/NGS | MF149939 | MF149943 | MF149948 | South Korea |  | Rattus norvegicus |
| E | Rn10-134/NGS | MF149938 | MF149942 | MF149947 | South Korea |  | Rattus norvegicus |
| E | Rn10-145 | MF149937 |  |  | South Korea |  | Rattus norvegicus |
| E | Rn10-134 | MF149936 |  |  | South Korea |  | Rattus norvegicus |
| E | Tchoupitoulas (TCH) |  |  | AF329389 | United Kingdom | | Rattus norvegicus |
| E | SR-11 | OK500096 | OK500097 | OK500098 | Japan |  | Rattus norvegicus |
| F | Humber | JX879770 | JX879768 | JX879769 | United Kingdom | | Rattus norvegicus |
| F | IR461 | KM948595 | AF458104 | AF329388 | United Kingdom | | Homo sapiens |
| F | Cherwell | KM948594 | KM948593 | KC626089 | United Kingdom | | Rattus norvegicus |
| F | Seoul-Baxter/NYC-D23 |  | KJ950864 | KJ950868 | USA |  | Rattus norvegicus |
| F | Seoul-Baxter/NYC-D1 |  | KJ950863^#^ | KJ950867^#^ | USA |  | Rattus norvegicus |
| F | Seoul-Baxter/NYC-D17 |  | KJ950862^#^ | KJ950866^#^ | USA |  | Rattus norvegicus |
| F | Seoul-Baxter/NYC-D3 |  | KJ950865^#^ | KJ950869^#^ | USA |  | Rattus norvegicus |
| F | ERIZE-ST-DIZIER/Hu/FRA/2014/2014.00479 | |  | KX064275 | France |  | Homo sapiens |
| F | ERIZE-ST-DIZIER/Rn/FRA/2014/2014.00417 | |  | KX064274 | France |  | Rattus norvegicus |
| F | TURCKHEIM/Rn/FRA/2016/2016.00033 | |  | KX064271 | France |  | Rattus norvegicus |
| F | TURCKHEIM/Hu/FRA/2016/2016.00044 | |  | KX064270 | France |  | Homo sapiens |
| F | IR162 | KX079474 | KX079475 | KX079476 | United Kingdom | | Rattus norvegicus |
| F | IR473 | KX079468^#^ | KX079469 | KX079470 | United Kingdom | | Rattus norvegicus |
| F | IR33 | KX079471^#^ | KX079472^#^ | KX079473^#^ | United Kingdom | | Rattus norvegicus |
| F | Gelderland_Rn84 |  | MG972934 | MG972939 | Netherlands |  | Rattus norvegicus |
| F | Gelderland_Rn22 |  |  | MG972937 | Netherlands |  | Rattus norvegicus |
| F | Gelderland_Rn33 |  |  | MG972935^#^ | Netherlands |  | Rattus norvegicus |
| F | 201701093/SEOV/Illinois_US/Rat | MK360803 | MK360792 | MK360784^#^ | USA |  | Rattus norvegicus |
| F | 201700683/SEOV/Illinois_US/Rat | MK360810^#^ | MK360793^#^ | MK360782^#^ | USA |  | Rattus norvegicus |
| F | 201700423/SEOV/Illinois_US/Rat | MK360807 | MK360797^#^ | MK360778^#^ | USA |  | Rattus norvegicus |
| F | 201701022/SEOV/Illinois_US/Rat | MK360808^#^ | MK360796^#^ | MK360777 | USA |  | Rattus norvegicus |
| F | 201802480/SEOV/Illinois_US/Rat | MK360800^#^ | |  | USA |  | Rattus norvegicus |
| F | 201700860/SEOV/Wisconsin_US/Rat | MK360813^#^ | MK360795^#^ |  | USA |  | Rattus norvegicus |
| F | 201701554/SEOV/Utah_US/Rat MK360806^#^ | | MK360788 | MK360783^#^ | USA |  | Rattus norvegicus |
| F | 201701321/SEOV/Colorado_US/Rat MK360811^#^ | | MK360790 | MK360780^#^ | USA |  | Rattus norvegicus |
| F | 201701555/SEOV/Utah_US/Rat |  | MK360787 |  | USA |  | Rattus norvegicus |
| F | 201700420/SEOV/Illinois_US/Rat |  | MK360798 ^#^ |  | USA |  | Rattus norvegicus |
| F | 201701593/SEOV/Colorado_US/Rat |  |  | MK360781 | USA |  | Rattus norvegicus |
| F | 201700048/SEOV/Illinois_US/Rat |  |  | MK360773 | USA |  | Rattus norvegicus |
| F | CVR/2019 | MZ343375 | MZ343376 | MZ343377 | United Kingdom | | Homo sapiens |
| G | YongjiaRn14 |  | GU592927 | GU592947 | China | Zhejiang | Rattus norvegicus |
| G | YongjiaRf45 |  | GU592926 | GU592943 | China | Zhejiang | Rattus flavipectus |
| G | ZJ5 |  | FJ811839 | FJ753400 | China | Zhejiang | Rattus norvegicus |
| G | Gou3 |  | AF145977 | AF184988 | China |  | Rattus rattus |
| G | GAW50/2021 | MZ504242 | MZ504240 | MZ504238 | China | Jiangxi | Rattus losea |
| G | GAW30/2021 | MZ504241 | MZ504239 | MZ504237 | China | Jiangxi | Rattus norvegicus |
| G | GAN36/2020 |  |  | MZ061897 | China | Jiangxi | Rattus norvegicus |
| G | SG42/2011 |  |  | KY807171 | China | Jiangxi | Apodemus agrarius |
| G | TGN07/2019 |  |  | MZ061896 | China | Jiangxi | Rattus norvegicus |
| G | TGN07/2018 |  |  | MZ061895 | China | Jiangxi | Rattus norvegicus |
| G | GAN08/2018 |  |  | MZ061894 | China | Jiangxi | Rattus norvegicus |
| G | AYN5/2018 |  |  | MZ061893^#^ | China | Jiangxi | Rattus norvegicus |
| G | AYN4/2018 |  |  | MZ061892^#^ | China | Jiangxi | Rattus norvegicus |
| G | AYN21/2018 |  |  | MZ061891 | China | Jiangxi | Rattus norvegicus |

* The virus strains reported by the present study; ^#^ Sequences with 100% homology; ^$^ Recombination Sequences

Table S2. Different clades of SEOV RdRp with amino acid substitutions

| Position | A | B | C | D | E | F | G |
| --- | --- | --- | --- | --- | --- | --- | --- |
| 66 | 65 I | 3 I | 2 I | 4 I | 11 I | 8 I | 2 V |
| 100 | 65 R | 3 R | 2 R | 4 R | 11 R | 7 R | 2 K |
| 102 | 65 T | 3 T | 2 T | 4 T | 11 T | 7 T | 2 S |
| 104 | 65 S | 3 S | 2 S | 4 S | 11 S | 7 S | 2 L |
| 105 | 65 L | 3 L | 2 L | 4 L | 11 L | 3 L, 4 S | 2 L |
| 133 | 65 K | 3 K | 2 R | 4 K | 11 K | 6 K, 1 R | 2 K |
| 140 | 65 M | 2 I, 1 M | 2 M | 4 M | 11 M | 7 M | 2 L |
| 233 | 65 I | 3 I | 2 V | 4 I | 11 I | 7 I | 2 I |
| 234 | 65 D | 3 D | 2 D | 4 D | 11 D | 7 D | 2 E |
| 237 | 65 G | 3 G | 2 G | 4 G | 11 R | 7 G | 2 G |
| 249 | 7 N, 55 S, 3 V | 3 N | 2 S | 4 S | 11 S | 7 S | 2 G |
| 254 | 65 T | 3 T | 2 T | 4 T | 11 T | 4 S, 3 T | 2 T |
| 273 | 65 E | 1 E, 2 K | 2 E | 4 E | 11 E | 7 E | 2 E |
| 277 | 64 G, 1 R | 3 G | 2 G | 4 G | 11 G | 7 G | 2 E |
| 280 | 4 I, 61 V | 3 I | 2 V | 4 V | 11 V | 7 V | 2A |
| 281 | 64 K, 1 R | 3 K | 2 R | 4 K | 11 K | 7 K | 2 R |
| 284 | 65 M | 3 M | 2 M | 4 M | 10 M, 1 V | 6 I, 1 M | 2 L |
| 297 | 65 T | 3 T | 2 T | 4 T | 11 T | 7 T | 2 S |
| 330 | 65 A | 3A | 2 T | 4A | 11A | 7A | 2A |
| 331 | 65 S | 3 S | 2 S | 4 S | 11 S | 7 S | 2A |
| 354 | 65 A | 3A | 2A | 4A | 11A | 7A | 2 S |
| 360 | 65 S | 3 N | 2 N | 4 N | 10 N, 1 S | 7 S | 2 N |
| 391 | 65 Q | 3 Q | 2 Q | 4 Q | 11 Q | 7 Q | 2 K |
| 401 | 65 S | 3 S | 2 S | 4 S | 11 S | 4 L, 3 S | 2 L |
| 507 | 65 S | 3 S | 2 S | 4 N | 11 S | 7 S | 2 S |
| 509 | 65 I | 3 I | 2 L | 4 I | 11 I | 7 I | 2 M |
| 548 | 65 A | 3A | 2A | 4A | 11A | 7A | 2 T |
| 559 | 65 V | 3 V | 2V | 4 V | 11 I | 7 V | 2 V |
| 601 | 65I | 3 I | 2 I | 4 T | 11 I | 7 I | 2 T |
| 644 | 65 V | 3 V | 2 V | 4 V | 11 V | 7 V | 2 I |
| 754 | 65 E | 3 E | 2 E | 4 E | 11 E | 7 E | 2 Q |
| 766 | 65 N | 3 N | 2 N | 4 N | 11 H | 7 N | 2 N |
| 770 | 65 I | 3 I | 2 I | 4 I | 11 I | 7 L | 2 I |
| 776 | 65 S | 3 S | 2 S | 4 S | 11 S | 7 S | 2 N |
| 778 | 65 E | 3 E | 2 E | 4 E | 11 E | 7 E | 2 D |
| 781 | 8 A, 57 V | 3 V | 2 V | 4 V | 11 V | 5 A, 2 V | 2 I |
| 800 | 65 I | 3 I | 2 I | 4 I | 2 I, 9 V | 7 I | 2 I |
| 816 | 65 Y | 3 Y | 2 Y | 4 Y | 11 Y | 7 Y | 2 F |
| 844 | 65 S | 3 S | 2 S | 4 S | 11 S | 7 S | 2A |
| 847 | 65 T | 3 T | 2 T | 4 T | 11 T | 7 T | 2 V |
| 930 | 65 R | 3 R | 2 R | 4 R | 11 R | 7 R | 2 K |
| 937 | 65 A | 3A | 2A | 4A | 11A | 7A | 2 S |
| 1039 | 65 D | 3 D | 2 D | 4 D | 11 D | 7 D | 2 E |
| 1042 | 65 N | 3 N | 2 N | 4 N | 11 N | 4 D, 3 N | 2 D |
| 1247 | 65 V | 3 V | 2 V | 4 V | 11 V | 7 V | 2 I |
| 1350 | 65 S | 3 S | 2 S | 4 S | 11 S | 7 S | 2A |
| 1470 | 65 N | 3 N | 2 N | 4 N | 11 N | 7 N | 2 H |
| 1478 | 65 T | 3 T | 2 T | 4 T | 11 T | 5 I, 2 T | 2 T |
| 1566 | 65 S | 3 S | 2 S | 4 S | 11 S | 4 N, 3 S | 2 S |
| 1591 | 65 R | 2 K, 1 R | 2 R | 4 R | 11 R | 4 K, 3 R | 2 R |
| 1596 | 65 I | 3 I | 2 I | 4 I | 11 I | 7 I | 2 V |
| 1675 | 64 I, 1 V | 3 I | 2 I | 4 I | 11 I | 7 I | 2 V |
| 1721 | 65 E | 3 E | 2 E | 4 E | 11 E | 5 D, 2 E | 2 E |
| 1749 | 64 K, 1 R | 3 K | 2 K | 4 K | 11 K | 7 K | 2 R |
| 1776 | 1 K, 64 R | 3 R | 2 R | 4 R | 11 R | 7 R | 2 K |
| 1847 | 65 Y | 3 Y | 2 H | 4 Y | 11 Y | 7 Y | 2 Y |
| 1884 | 65 I | 3 I | 2 I | 4 I | 11 I | 7 I | 2 V |
| 1939 | 65G | 3 G | 2 G | 4 G | 11 G | 5 A, 2 G | 2 G |
| 1949 | 65I | 3 I | 2 M | 4 I | 9 I, 2 L | 7 I | 2 I |
| 1960 | 65 L | 3 L | 2 L | 4 L | 11 L | 7 L | 2 I |
| 1974 | 65 D | 3 D | 2 D | 4 D | 11 D | 7 D | 2 E |
| 2071 | 65 F | 3 F | 2 F | 4 L | 11 F | 7 F | 2 F |
| 2074 | 65 K | 3 K | 2 K | 4 K | 10 K, 1 R | 7 K | 2 R |
| 2103 | 63 D, 2 E | 3 D | 2 D | 4 E | 11 D | 1 A, 6 D | 2 D |

Red marked: Frequency of the mutation site in corresponding group was 100%.

Table S3. Different clades of SEOV Gn with amino acid substitutions

| Position | A | B | C | D | E | F | G |
| --- | --- | --- | --- | --- | --- | --- | --- |
| 3 | 85 S | 14 S | 4 S | 30 S | 15 S | 12 S | 6 G |
| 11 | 1 A, 1 I, 83 V | 14 V | 4A | 30 V | 1 I, 14 V | 12 V | 1 T, 5 V |
| 15 | 84 F, 1 V | 14 F | 4 F | 30 F | 15 F | 6 F, 6 S | 3 L, 3 S |
| 24 | 1 K, 84 R | 14 R | 4 R | 30 R | 15 R | 1 K, 11 R | 6 K |
| 48 | 85 S | 14 S | 4 S | 30 S | 15 S | 12 S | 6P |
| 90 | 85 N | 14 N | 4 S | 23 N, 7 S | 15 N | 12 N | 6 S |
| 133 | 3 N, 82 S | 14 S | 4 S | 30 S | 15 S | 12 S | 6 T |
| 167 | 85 V | 14 V | 4 V | 30 V | 15 V | 12 V | 6 I |
| 186 | 85 P | 14 P | 4A | 30 P | 15 P | 12P | 1 H, 5 P |
| 201 | 85 I | 3 I, 11 V | 4 I | 30 I | 15 I | 12 I | 6 I |
| 207 | 85 I | 14 I | 4 I | 30 I | 15 I | 12 I | 1 I, 5 V |
| 211 | 85 I | 14 I | 4 I | 30 I | 15 I | 10 I, 2 V | 6 V |
| 237 | 85 T | 14 T | 4 T | 30 T | 15 T | 12 T | 6 S |
| 421 | 5 I, 80 V | 14 V | 4 V | 7 I, 23 V | 15 V | 2 I, 10 V | 6 I |
| 425 | 85 V | 14 V | 1 I, 3 V | 30 V | 15 V | 12 V | 6 V |
| 487 | 85 I | 3 I, 11 T | 4 I | 30 I | 15 I | 12 I | 4 I, 2 V |
| 503 | 85 I | 14 I | 4 I | 28 I, 2 V | 15 I | 10 I, 2 V | 6 V |
| 506 | 85 V | 14 V | 4 V | 30 V | 15 V | 12 V | 6 F |
| 606 | 85 Q | 14 Q | 4 Q | 2 K, 28 Q | 15 Q | 8 P, 4 Q | 6 Q |
| 632 | 85 T | 14 T | 4 T | 30 T | 15 T | 12 T | 4 I, 2 V |
| 633 | 85 L | 10 I, 4 L | 4 L | 30 L | 15 L | 12 L | 6 L |
| 636 | 84 I, 1 V | 14 I | 4 I | 30 I | 15 I | 12 I | 6 V |

Red marked: Frequency of the mutation site in corresponding group was 100%.

Table S4. Different clades of SEOV Gc with amino acid substitutions

| Position | A | B | C | D | E | F | G |
| --- | --- | --- | --- | --- | --- | --- | --- |
| 682 | 85 K | 13 K, 1 R | 4 K | 30 K | 6 K, 9 R | 12 K | 6 K |
| 795 | 85 I | 14 I | 4 I | 30 I | 15 I | 6 I, 6 V | 6 I |
| 908 | 85 I | 13 I, 1 T | 2 I, 2 T | 30 I | 15 I | 12 I | 6 V |
| 913 | 85 K | 14 K | 4 K | 30 K | 15 K | 12 K | 6 R |
| 930 | 85 I | 14 I | 4 I | 30 I | 15 I | 12 I | 2 I, 4 V |
| 1052 | 85 T | 14 T | 4 T | 30 T | 15 T | 2 N, 10 T | 6 N |
| 1109 | 85 V | 12 A, 2 V | 4 V | 30 V | 15 V | 12 V | 6 V |

Red marked: Frequency of the mutation site in corresponding group was 100%.

Table S5. Different clades of SEOV NP with amino acid substitutions

| Position | A | B | C | D | E | F | G |
| --- | --- | --- | --- | --- | --- | --- | --- |
| 233 | 119 T | 21 T | 7 T | 31 T | 15 T | 15 T | 8S，4T |
| 247 | 119 M | 21 M | 7 M | 30 M, 1 T | 15 M | 9 I, 6 M | 12 M |
| 288 | 116 A, 3 S | 1 P, 20 S | 7 S | 16 A, 15 S | 15 S | 15 S | 12 S |

Red marked: Frequency of the mutation site in corresponding group was 100%.

Table S6. Positive selection points identified by PAML

| Gene | ω | InLNull | InL A | 2InDL | Significance | Site |
| --- | --- | --- | --- | --- | --- | --- |
| L | 0.02089 | -15612.677 | -15616.053 | 6.753 | 9.35×10^-3^ | - |
| M | 0.03447 | -16423.508 | -16426.486 | 5.956 | 1.47×10^-2^ | 11 |
| S | 0.06275 | -10676.399 | -10685.862 | 18.926 | 1.36×10^-5^ | 259 |

| Table S7. The nucleotide composition of L segment (%) | | | | | | | | | | | | | |
| --- | --- | --- | --- | --- | --- | --- | --- | --- | --- | --- | --- | --- | --- |
| **SEQUENCES \ PARAMETERS** | **A** | **C** | **U** | **G** | **GC** | **AU** | **GC1** | **GC2** | **A3** | **C3** | **U3** | **G3** | **GC3** |
| 201700423/SEOV/Illinois_US/Rat | 32.39 | 16.28 | 30.30 | 21.03 | 37.31 | 62.69 | 45.96 | 34.20 | 43.45 | 15.77 | 46.83 | 23.15 | 31.78 |
| 201701093/SEOV/Illinois_US/Rat | 32.42 | 16.25 | 30.34 | 20.99 | 37.24 | 62.76 | 45.86 | 34.20 | 43.45 | 15.65 | 47.01 | 23.10 | 31.64 |
| 80-39 | 32.53 | 16.39 | 29.89 | 21.19 | 37.58 | 62.42 | 46.14 | 34.29 | 44.03 | 15.88 | 45.62 | 23.79 | 32.30 |
| 93HBJ20 | 32.47 | 16.45 | 29.76 | 21.33 | 37.78 | 62.22 | 45.77 | 34.29 | 43.24 | 16.39 | 45.13 | 24.66 | 33.27 |
| 93HBQ3 | 32.48 | 16.45 | 29.76 | 21.31 | 37.76 | 62.24 | 45.82 | 34.25 | 43.37 | 16.39 | 45.07 | 24.59 | 33.22 |
| 93HBQ4 | 32.48 | 16.51 | 29.72 | 21.28 | 37.79 | 62.21 | 45.96 | 34.25 | 43.43 | 16.49 | 45.02 | 24.45 | 33.18 |
| 93HBX10 | 32.48 | 16.39 | 29.94 | 21.19 | 37.58 | 62.42 | 45.59 | 34.34 | 43.24 | 16.12 | 45.64 | 24.34 | 32.81 |
| 93HBX11 | 32.50 | 16.47 | 29.76 | 21.28 | 37.75 | 62.25 | 45.77 | 34.25 | 43.41 | 16.46 | 45.04 | 24.50 | 33.22 |
| 93HBX12 | 32.50 | 16.45 | 29.76 | 21.30 | 37.75 | 62.25 | 45.77 | 34.25 | 43.43 | 16.45 | 45.01 | 24.52 | 33.22 |
| Cherwell | 32.36 | 16.33 | 30.27 | 21.05 | 37.38 | 62.62 | 45.86 | 34.20 | 43.20 | 15.95 | 46.71 | 23.38 | 32.06 |
| CVR/2019 | 32.34 | 16.37 | 30.25 | 21.03 | 37.41 | 62.59 | 45.86 | 34.15 | 43.16 | 16.12 | 46.59 | 23.40 | 32.20 |
| DN2 | 32.37 | 16.45 | 29.97 | 21.21 | 37.65 | 62.35 | 46.05 | 34.29 | 43.42 | 16.11 | 45.79 | 23.92 | 32.62 |
| DPRK08 | 32.23 | 16.28 | 30.08 | 21.41 | 37.69 | 62.31 | 45.72 | 34.39 | 42.72 | 15.82 | 45.97 | 24.84 | 32.95 |
| FJ35 | 32.54 | 16.43 | 29.83 | 21.19 | 37.62 | 62.38 | 46.10 | 34.39 | 43.93 | 15.82 | 45.62 | 24.01 | 32.39 |
| Fj372/2013 | 32.56 | 16.22 | 30.08 | 21.14 | 37.36 | 62.64 | 45.86 | 34.29 | 43.85 | 15.36 | 46.26 | 23.81 | 31.92 |
| GAW30/2021 | 32.31 | 15.89 | 30.76 | 21.03 | 36.93 | 63.07 | 45.40 | 34.25 | 43.02 | 15.12 | 47.98 | 22.91 | 31.13 |
| GAW50/2021 | 32.37 | 15.94 | 30.68 | 21.00 | 36.94 | 63.06 | 45.49 | 34.15 | 43.24 | 15.24 | 47.75 | 22.86 | 31.18 |
| HBB35/2002 | 32.51 | 16.33 | 30.11 | 21.05 | 37.38 | 62.62 | 46.14 | 34.34 | 43.91 | 15.47 | 46.53 | 23.23 | 31.64 |
| HBB36/2002 | 32.47 | 16.36 | 30.07 | 21.11 | 37.47 | 62.53 | 46.24 | 34.34 | 43.72 | 15.47 | 46.47 | 23.51 | 31.83 |
| HBCD1/2017 | 32.42 | 16.33 | 30.11 | 21.14 | 37.47 | 62.53 | 45.86 | 34.29 | 43.30 | 15.71 | 46.35 | 23.93 | 32.25 |
| HBCD1/2018 | 32.43 | 16.26 | 30.13 | 21.17 | 37.44 | 62.56 | 45.91 | 34.29 | 43.45 | 15.46 | 46.39 | 24.02 | 32.11 |
| HBCD13/2019 | 32.50 | 16.31 | 30.08 | 21.11 | 37.42 | 62.58 | 45.86 | 34.29 | 43.62 | 15.50 | 46.15 | 24.02 | 32.11 |
| HBCD3/2017 | 32.45 | 16.26 | 30.13 | 21.16 | 37.42 | 62.58 | 45.91 | 34.29 | 43.52 | 15.46 | 46.39 | 23.95 | 32.06 |
| HBCD4/2017 | 32.39 | 16.33 | 30.10 | 21.19 | 37.52 | 62.48 | 45.86 | 34.34 | 43.27 | 15.77 | 46.24 | 23.99 | 32.34 |
| HBCD44/2021 | 32.34 | 16.31 | 30.14 | 21.21 | 37.52 | 62.48 | 45.96 | 34.29 | 43.10 | 15.59 | 46.47 | 24.15 | 32.30 |
| HBCD52/2002 | 32.48 | 16.31 | 30.03 | 21.17 | 37.48 | 62.52 | 45.86 | 34.29 | 43.56 | 15.73 | 46.05 | 23.97 | 32.30 |
| HBCD55/2002 | 32.45 | 16.26 | 30.13 | 21.16 | 37.42 | 62.58 | 45.86 | 34.20 | 43.43 | 15.60 | 46.32 | 24.02 | 32.20 |
| HBCD55/2021 | 32.43 | 16.22 | 30.17 | 21.17 | 37.39 | 62.61 | 45.86 | 34.29 | 43.45 | 15.34 | 46.50 | 24.02 | 32.02 |
| HBCD56/2002 | 32.50 | 16.28 | 30.05 | 21.17 | 37.45 | 62.55 | 45.82 | 34.29 | 43.63 | 15.67 | 46.05 | 23.97 | 32.25 |
| HBCD6/2017 | 32.40 | 16.33 | 30.10 | 21.17 | 37.50 | 62.50 | 45.91 | 34.29 | 43.30 | 15.71 | 46.30 | 24.01 | 32.30 |
| HBCD8/2017 | 32.47 | 16.23 | 30.14 | 21.16 | 37.39 | 62.61 | 45.96 | 34.29 | 43.55 | 15.29 | 46.53 | 23.93 | 31.92 |
| HBCD9/2017 | 32.45 | 16.28 | 30.11 | 21.16 | 37.44 | 62.56 | 45.91 | 34.25 | 43.48 | 15.52 | 46.33 | 24.04 | 32.16 |
| HBCZ88/1999 | 32.54 | 16.42 | 29.88 | 21.16 | 37.58 | 62.42 | 46.10 | 34.39 | 43.87 | 15.83 | 45.82 | 23.76 | 32.25 |
| HBH51/2021 | 32.33 | 16.37 | 29.89 | 21.41 | 37.78 | 62.22 | 45.96 | 34.48 | 43.01 | 15.82 | 45.73 | 24.73 | 32.90 |
| HBL131/2007 | 32.28 | 16.25 | 30.19 | 21.28 | 37.53 | 62.47 | 45.86 | 34.20 | 42.78 | 15.60 | 46.50 | 24.50 | 32.53 |
| HBL3/2005 | 32.51 | 16.39 | 29.91 | 21.19 | 37.58 | 62.42 | 46.05 | 34.43 | 43.81 | 15.76 | 45.85 | 23.81 | 32.25 |
| HBQ1/2015 | 32.36 | 16.23 | 30.19 | 21.22 | 37.45 | 62.55 | 45.86 | 34.29 | 43.10 | 15.41 | 46.59 | 24.22 | 32.20 |
| HBQ15/2018 | 32.33 | 16.33 | 30.05 | 21.30 | 37.62 | 62.38 | 45.86 | 34.25 | 43.07 | 15.90 | 45.97 | 24.43 | 32.76 |
| HBQ2/2015 | 32.34 | 16.23 | 30.16 | 21.27 | 37.50 | 62.50 | 45.82 | 34.29 | 43.13 | 15.47 | 46.35 | 24.43 | 32.39 |
| HBQ24/2011 | 32.33 | 16.28 | 30.11 | 21.28 | 37.56 | 62.44 | 45.82 | 34.34 | 42.98 | 15.65 | 46.30 | 24.43 | 32.53 |
| HBQ4/2012 | 32.34 | 16.29 | 30.10 | 21.27 | 37.56 | 62.44 | 45.77 | 34.34 | 42.98 | 15.71 | 46.24 | 24.45 | 32.57 |
| HBQ47/2021 | 32.34 | 16.25 | 30.14 | 21.27 | 37.52 | 62.48 | 45.82 | 34.34 | 43.10 | 15.52 | 46.33 | 24.36 | 32.39 |
| HBQ49/2004 | 32.36 | 16.34 | 30.03 | 21.27 | 37.61 | 62.39 | 45.86 | 34.29 | 43.10 | 15.83 | 46.00 | 24.43 | 32.67 |
| HBQ5/2015 | 32.42 | 16.34 | 30.07 | 21.17 | 37.52 | 62.48 | 45.86 | 34.29 | 43.30 | 15.77 | 46.18 | 24.09 | 32.39 |
| HBQ50/2021 | 32.48 | 16.28 | 30.08 | 21.16 | 37.44 | 62.56 | 45.77 | 34.34 | 43.55 | 15.59 | 46.18 | 24.01 | 32.20 |
| HBQ57/2021 | 32.43 | 16.33 | 30.08 | 21.16 | 37.48 | 62.52 | 45.86 | 34.20 | 43.35 | 15.77 | 46.18 | 24.11 | 32.39 |
| HBQ58/2021 | 32.34 | 16.29 | 30.13 | 21.24 | 37.53 | 62.47 | 45.91 | 34.29 | 43.07 | 15.58 | 46.39 | 24.31 | 32.39 |
| HBQ60/2021 | 32.43 | 16.33 | 30.08 | 21.16 | 37.48 | 62.52 | 45.86 | 34.20 | 43.35 | 15.77 | 46.18 | 24.11 | 32.39 |
| HBQ62/2001 | 32.43 | 16.28 | 30.10 | 21.19 | 37.47 | 62.53 | 45.86 | 34.34 | 43.43 | 15.53 | 46.30 | 24.06 | 32.20 |
| HBQ63/2001 | 32.39 | 16.39 | 30.03 | 21.19 | 37.58 | 62.42 | 45.77 | 34.29 | 43.17 | 16.06 | 45.94 | 24.16 | 32.67 |
| HBQ63/2021 | 32.39 | 16.29 | 30.07 | 21.25 | 37.55 | 62.45 | 45.86 | 34.34 | 43.30 | 15.65 | 46.12 | 24.26 | 32.43 |
| HBQ64/2022 | 32.50 | 16.26 | 30.08 | 21.16 | 37.42 | 62.58 | 45.82 | 34.29 | 43.65 | 15.60 | 46.14 | 23.92 | 32.16 |
| HBQ65/2009 | 32.39 | 16.37 | 30.05 | 21.19 | 37.56 | 62.44 | 45.96 | 34.29 | 43.26 | 15.82 | 46.15 | 24.09 | 32.43 |
| HBQ7/2012 | 32.43 | 16.25 | 30.13 | 21.19 | 37.44 | 62.56 | 45.77 | 34.29 | 43.40 | 15.59 | 46.24 | 24.06 | 32.25 |
| HBQ72/2000 | 32.39 | 16.39 | 30.03 | 21.19 | 37.58 | 62.42 | 45.77 | 34.29 | 43.17 | 16.06 | 45.94 | 24.16 | 32.67 |
| HBQ73/2000 | 32.36 | 16.28 | 30.14 | 21.22 | 37.50 | 62.50 | 45.86 | 34.29 | 43.10 | 15.59 | 46.41 | 24.22 | 32.34 |
| HBQ74/2000 | 32.40 | 16.33 | 30.05 | 21.22 | 37.55 | 62.45 | 45.91 | 34.29 | 43.30 | 15.71 | 46.12 | 24.22 | 32.43 |
| HBQ75/2000 | 32.51 | 16.37 | 30.05 | 21.07 | 37.44 | 62.56 | 46.14 | 34.34 | 43.82 | 15.60 | 46.38 | 23.37 | 31.83 |
| HBT4/2016 | 32.42 | 16.22 | 30.16 | 21.21 | 37.42 | 62.58 | 45.82 | 34.29 | 43.36 | 15.41 | 46.41 | 24.15 | 32.16 |
| HBT41/2012 | 32.45 | 16.34 | 30.07 | 21.14 | 37.48 | 62.52 | 45.91 | 34.25 | 43.49 | 15.83 | 46.12 | 23.86 | 32.30 |
| HBT43/2012 | 32.42 | 16.34 | 30.10 | 21.14 | 37.48 | 62.52 | 45.96 | 34.29 | 43.36 | 15.71 | 46.35 | 23.86 | 32.20 |
| HBT5/2016 | 32.42 | 16.23 | 30.14 | 21.21 | 37.44 | 62.56 | 45.86 | 34.29 | 43.36 | 15.41 | 46.41 | 24.15 | 32.16 |
| HBT52/2013 | 32.33 | 16.33 | 30.07 | 21.28 | 37.61 | 62.39 | 45.82 | 34.29 | 42.91 | 15.83 | 46.12 | 24.52 | 32.71 |
| HBT6/2015 | 32.37 | 16.29 | 30.08 | 21.25 | 37.55 | 62.45 | 45.86 | 34.29 | 43.04 | 15.59 | 46.30 | 24.45 | 32.48 |
| HBT60/2013 | 32.36 | 16.36 | 30.07 | 21.22 | 37.58 | 62.42 | 45.91 | 34.29 | 43.13 | 15.82 | 46.15 | 24.24 | 32.53 |
| HBT61/2014 | 32.39 | 16.33 | 30.08 | 21.21 | 37.53 | 62.47 | 45.82 | 34.29 | 43.23 | 15.83 | 46.12 | 24.15 | 32.48 |
| HBT62/2014 | 32.39 | 16.36 | 30.05 | 21.21 | 37.56 | 62.44 | 45.86 | 34.25 | 43.23 | 15.95 | 46.00 | 24.16 | 32.57 |
| HBT63/2013 | 32.36 | 16.34 | 30.08 | 21.22 | 37.56 | 62.44 | 45.91 | 34.29 | 43.13 | 15.76 | 46.21 | 24.24 | 32.48 |
| HBT64/2014 | 32.30 | 16.28 | 30.11 | 21.31 | 37.59 | 62.41 | 45.82 | 34.34 | 42.95 | 15.71 | 46.18 | 24.47 | 32.62 |
| HBT7/2016 | 32.50 | 16.29 | 30.13 | 21.08 | 37.38 | 62.62 | 45.86 | 34.29 | 43.68 | 15.65 | 46.35 | 23.58 | 31.97 |
| HN1 | 32.56 | 16.16 | 30.30 | 20.99 | 37.14 | 62.86 | 45.82 | 34.39 | 43.92 | 15.05 | 46.98 | 23.21 | 31.23 |
| Humber | 32.37 | 16.47 | 30.03 | 21.13 | 37.59 | 62.41 | 46.10 | 34.48 | 43.50 | 15.79 | 46.07 | 23.78 | 32.20 |
| IR162 | 32.36 | 16.33 | 30.19 | 21.13 | 37.45 | 62.55 | 46.05 | 34.25 | 43.22 | 15.70 | 46.50 | 23.76 | 32.06 |
| IR461 | 32.34 | 16.39 | 30.14 | 21.13 | 37.52 | 62.48 | 46.05 | 34.34 | 43.22 | 15.81 | 46.36 | 23.76 | 32.16 |
| JiangxiXianjianRn-07-2011 | 32.61 | 16.23 | 30.16 | 21.00 | 37.24 | 62.76 | 45.54 | 34.20 | 43.81 | 15.71 | 46.24 | 23.61 | 31.97 |
| JiangxiXinjianRn-09-2011 | 32.57 | 16.25 | 30.11 | 21.07 | 37.31 | 62.69 | 45.63 | 34.25 | 43.72 | 15.70 | 46.15 | 23.81 | 32.06 |
| L99 | 32.59 | 16.42 | 29.99 | 21.00 | 37.42 | 62.58 | 46.19 | 34.34 | 44.11 | 15.66 | 46.20 | 23.13 | 31.74 |
| LN01 | 32.57 | 16.25 | 30.05 | 21.13 | 37.38 | 62.62 | 45.86 | 34.25 | 43.97 | 15.60 | 46.03 | 23.70 | 32.02 |
| LN02 | 32.45 | 16.25 | 30.14 | 21.16 | 37.41 | 62.59 | 45.86 | 34.34 | 43.55 | 15.41 | 46.41 | 23.93 | 32.02 |
| LN03 | 32.42 | 16.33 | 30.07 | 21.19 | 37.52 | 62.48 | 45.86 | 34.29 | 43.31 | 15.77 | 46.12 | 24.13 | 32.39 |
| LN06 | 32.45 | 16.31 | 29.96 | 21.28 | 37.59 | 62.41 | 46.00 | 34.43 | 43.58 | 15.59 | 46.00 | 24.11 | 32.34 |
| LYON/Rn/FRA/2013/LYO852 | 32.11 | 16.47 | 29.96 | 21.47 | 37.93 | 62.07 | 46.05 | 34.25 | 42.11 | 16.13 | 45.91 | 25.27 | 33.50 |
| Rn10-134 | 32.54 | 16.34 | 30.05 | 21.07 | 37.41 | 62.59 | 46.19 | 34.39 | 44.32 | 15.45 | 46.12 | 23.21 | 31.64 |
| Rn10-134/NGS | 32.59 | 16.31 | 29.99 | 21.11 | 37.42 | 62.58 | 46.14 | 34.25 | 44.29 | 15.58 | 45.91 | 23.49 | 31.88 |
| Rn10-145 | 32.59 | 16.33 | 30.07 | 21.02 | 37.35 | 62.65 | 46.10 | 34.39 | 44.45 | 15.45 | 46.12 | 23.09 | 31.55 |
| Rn10-145/NGS | 32.59 | 16.25 | 30.03 | 21.13 | 37.38 | 62.62 | 46.10 | 34.29 | 44.29 | 15.40 | 46.09 | 23.51 | 31.74 |
| Rn11-44/NGS | 32.56 | 16.37 | 29.86 | 21.21 | 37.58 | 62.42 | 46.00 | 34.29 | 43.87 | 15.89 | 45.58 | 23.99 | 32.43 |
| Rn11-53/NGS | 32.56 | 16.37 | 29.93 | 21.14 | 37.52 | 62.48 | 46.19 | 34.25 | 44.22 | 15.82 | 45.68 | 23.56 | 32.11 |
| SOV/Rn18-1 | 32.67 | 16.40 | 29.80 | 21.13 | 37.53 | 62.47 | 45.96 | 34.39 | 44.17 | 15.79 | 45.52 | 23.81 | 32.25 |
| SOV/Rn19-5 | 32.79 | 16.39 | 29.88 | 20.94 | 37.33 | 62.67 | 46.10 | 34.29 | 44.87 | 15.70 | 45.68 | 23.00 | 31.60 |
| SR-11 | 32.59 | 16.50 | 29.79 | 21.13 | 37.62 | 62.38 | 45.86 | 34.34 | 44.26 | 16.49 | 44.96 | 23.55 | 32.67 |
| Tchoupitoulas/POR | 32.59 | 16.29 | 30.00 | 21.11 | 37.41 | 62.59 | 45.86 | 34.20 | 43.99 | 15.65 | 45.88 | 23.83 | 32.16 |
| Z37 | 32.53 | 16.40 | 29.96 | 21.11 | 37.52 | 62.48 | 45.91 | 34.29 | 43.91 | 16.00 | 45.64 | 23.70 | 32.34 |
| ZT10 | 32.57 | 16.36 | 29.94 | 21.13 | 37.48 | 62.52 | 45.86 | 34.29 | 44.07 | 15.95 | 45.52 | 23.70 | 32.30 |
| ZT71 | 32.56 | 16.40 | 29.94 | 21.10 | 37.50 | 62.50 | 45.77 | 34.39 | 44.01 | 16.06 | 45.52 | 23.62 | 32.34 |
|  |  |  |  |  |  |  |  |  |  |  |  |  |  |
| Mean | 32.44 | 16.32 | 30.06 | 21.17 | 37.49 | 62. 51 | 45.90 | 34.30 | 43.48 | 15. 73 | 46.13 | 23.96 | 32.28 |
| Standard deviation | 0.10 | 0.09 | 0.16 | 0.09 | 0.15 | 0.15 | 0.15 | 0.06 | 0.43 | 0.28 | 0.48 | 0.45 | 0.43 |

| Table S8. The nucleotide composition of M segment (%) | | | | | | | | | | | | | |
| --- | --- | --- | --- | --- | --- | --- | --- | --- | --- | --- | --- | --- | --- |
| SEQUENCES \ PARAMETERS | A | C | U | G | GC | AU | GC1 | GC2 | A3 | C3 | U3 | G3 | GC3 |
| 5CSG | 30.36 | 18.46 | 29.69 | 21.49 | 39.95 | 60.05 | 44.71 | 41.89 | 41.89 | 21.32 | 44.91 | 19.37 | 33.25 |
| ZT10 | 30.36 | 18.37 | 29.78 | 21.49 | 39.86 | 60.14 | 44.71 | 41.45 | 41.77 | 21.32 | 44.81 | 19.83 | 33.42 |
| ZJ5 | 30.42 | 18.31 | 29.75 | 21.52 | 39.83 | 60.17 | 45.59 | 41.71 | 42.86 | 20.24 | 45.45 | 19.02 | 32.19 |
| Z37 | 30.25 | 18.40 | 29.69 | 21.66 | 40.06 | 59.94 | 44.71 | 41.53 | 41.15 | 21.24 | 44.75 | 20.66 | 33.95 |
| YY27 | 30.28 | 18.49 | 29.72 | 21.52 | 40.01 | 59.99 | 45.15 | 41.89 | 42.07 | 21.08 | 45.08 | 19.23 | 32.98 |
| YongjiaRn14 | 29.81 | 18.90 | 29.10 | 22.19 | 41.09 | 58.91 | 45.59 | 41.71 | 40.27 | 22.54 | 43.12 | 21.92 | 35.98 |
| YongjiaRf45 | 29.81 | 18.72 | 29.31 | 22.16 | 40.89 | 59.11 | 45.33 | 41.53 | 40.35 | 22.29 | 43.40 | 22.04 | 35.80 |
| XiaotangshanRn7 | 30.19 | 18.52 | 29.72 | 21.58 | 40.09 | 59.91 | 45.24 | 41.71 | 41.63 | 21.19 | 45.08 | 19.67 | 33.33 |
| WuhanRn98 | 30.57 | 18.46 | 29.78 | 21.19 | 39.65 | 60.35 | 44.71 | 41.89 | 42.93 | 21.32 | 45.13 | 18.02 | 32.36 |
| WuhanRn67u | 30.54 | 18.43 | 29.78 | 21.25 | 39.68 | 60.32 | 44.71 | 41.80 | 42.73 | 21.32 | 45.13 | 18.32 | 32.54 |
| WuhanRn58 | 30.54 | 18.43 | 29.78 | 21.25 | 39.68 | 60.32 | 44.71 | 41.89 | 42.80 | 21.21 | 45.13 | 18.29 | 32.45 |
| WuhanRn57 | 30.57 | 18.46 | 29.78 | 21.19 | 39.65 | 60.35 | 44.71 | 41.89 | 42.93 | 21.32 | 45.13 | 18.02 | 32.36 |
| WuhanRn53 | 30.51 | 18.46 | 29.78 | 21.25 | 39.71 | 60.29 | 44.71 | 41.98 | 42.80 | 21.30 | 45.08 | 18.16 | 32.45 |
| WuhanRn25 | 30.54 | 18.46 | 29.78 | 21.22 | 39.68 | 60.32 | 44.71 | 41.80 | 42.73 | 21.43 | 45.13 | 18.18 | 32.54 |
| WuhanRf49 | 30.51 | 18.46 | 29.78 | 21.25 | 39.71 | 60.29 | 44.71 | 41.98 | 42.80 | 21.30 | 45.08 | 18.16 | 32.45 |
| WuhanRf12 | 30.51 | 18.43 | 29.81 | 21.25 | 39.68 | 60.32 | 44.71 | 41.89 | 42.68 | 21.21 | 45.24 | 18.29 | 32.45 |
| WuhanRf08 | 30.51 | 18.52 | 29.75 | 21.22 | 39.74 | 60.26 | 44.80 | 41.89 | 42.63 | 21.32 | 45.13 | 18.27 | 32.54 |
| WuhanRf02 | 30.31 | 18.61 | 29.66 | 21.43 | 40.04 | 59.96 | 45.41 | 42.15 | 42.45 | 21.23 | 45.04 | 18.33 | 32.54 |
| WuhanMm24 | 30.22 | 18.81 | 29.39 | 21.58 | 40.39 | 59.61 | 44.80 | 41.71 | 41.50 | 22.62 | 43.61 | 19.83 | 34.66 |
| WuhanMm13 | 30.51 | 18.43 | 29.81 | 21.25 | 39.68 | 60.32 | 44.71 | 41.80 | 42.56 | 21.21 | 45.24 | 18.43 | 32.54 |
| Tchoupitoulas/POR | 30.36 | 18.90 | 29.22 | 21.52 | 40.42 | 59.58 | 44.97 | 41.71 | 42.07 | 22.79 | 43.09 | 19.56 | 34.57 |
| SR-11 | 30.63 | 18.75 | 29.34 | 21.28 | 40.04 | 59.96 | 44.97 | 41.71 | 43.00 | 22.19 | 43.72 | 18.54 | 33.42 |
| SOV/Rn19-5 | 30.39 | 18.49 | 29.72 | 21.40 | 39.89 | 60.11 | 44.53 | 41.53 | 41.82 | 21.75 | 44.59 | 19.39 | 33.60 |
| SOV/Rn18-1 | 30.25 | 18.72 | 29.45 | 21.58 | 40.30 | 59.70 | 44.62 | 41.71 | 41.38 | 22.51 | 43.83 | 19.83 | 34.57 |
| Singapore/06RN46 | 30.39 | 18.43 | 29.66 | 21.52 | 39.95 | 60.05 | 44.53 | 41.80 | 41.89 | 21.43 | 44.59 | 19.67 | 33.51 |
| ShuangyangRn470 | 30.13 | 18.64 | 29.48 | 21.75 | 40.39 | 59.61 | 44.89 | 41.71 | 41.26 | 21.84 | 43.89 | 20.74 | 34.57 |
| Seoul-Baxter/NYC-D23 | 30.45 | 18.78 | 29.48 | 21.28 | 40.06 | 59.94 | 45.33 | 41.53 | 42.44 | 22.10 | 44.42 | 18.54 | 33.33 |
| RuianRn242 | 30.13 | 18.69 | 29.54 | 21.63 | 40.33 | 59.67 | 45.06 | 41.62 | 41.26 | 22.08 | 44.26 | 20.08 | 34.30 |
| RuianRn180 | 30.07 | 18.67 | 29.57 | 21.69 | 40.36 | 59.64 | 45.24 | 41.62 | 40.84 | 21.75 | 44.70 | 20.39 | 34.22 |
| Gelderland_Rn84 | 30.66 | 18.69 | 29.57 | 21.08 | 39.77 | 60.23 | 44.89 | 41.62 | 43.23 | 21.84 | 44.32 | 18.04 | 32.80 |
| Rn11-53/NGS | 30.25 | 18.84 | 29.34 | 21.58 | 40.42 | 59.58 | 44.89 | 41.71 | 41.52 | 22.70 | 43.46 | 19.78 | 34.66 |
| Rn11-44/NGS | 30.25 | 18.93 | 29.28 | 21.55 | 40.48 | 59.52 | 44.80 | 41.71 | 41.50 | 23.03 | 43.24 | 19.70 | 34.92 |
| Rn10-145/NGS | 30.19 | 18.99 | 29.19 | 21.63 | 40.62 | 59.38 | 44.89 | 41.71 | 41.28 | 23.24 | 42.92 | 20.05 | 35.27 |
| Rn10-134/NGS | 30.13 | 18.90 | 29.28 | 21.69 | 40.59 | 59.41 | 44.97 | 41.71 | 41.15 | 22.92 | 43.24 | 20.16 | 35.10 |
| Rn10-145 | 30.28 | 18.78 | 29.37 | 21.58 | 40.36 | 59.64 | 44.97 | 41.53 | 41.70 | 22.59 | 43.46 | 19.81 | 34.57 |
| R22 | 30.54 | 18.58 | 29.66 | 21.22 | 39.80 | 60.20 | 44.09 | 41.89 | 42.47 | 21.95 | 44.32 | 18.87 | 33.42 |
| LYON/Rn/FRA/2013/LYO852 | 30.31 | 18.52 | 29.66 | 21.52 | 40.04 | 59.96 | 44.62 | 41.71 | 41.82 | 21.75 | 44.48 | 19.56 | 33.77 |
| LongwanRn581 | 30.34 | 18.52 | 29.60 | 21.55 | 40.06 | 59.94 | 44.89 | 41.53 | 41.87 | 21.65 | 44.37 | 19.81 | 33.77 |
| LN06 | 30.28 | 18.55 | 29.69 | 21.49 | 40.04 | 59.96 | 45.06 | 41.62 | 41.87 | 21.54 | 44.81 | 19.39 | 33.42 |
| LN05 | 30.25 | 18.52 | 29.66 | 21.58 | 40.09 | 59.91 | 44.71 | 41.62 | 41.63 | 21.86 | 44.37 | 19.81 | 33.95 |
| LN04 | 30.28 | 18.43 | 29.75 | 21.55 | 39.98 | 60.02 | 45.06 | 41.62 | 41.87 | 21.10 | 45.02 | 19.67 | 33.25 |
| LN03 | 30.31 | 18.55 | 29.60 | 21.55 | 40.09 | 59.91 | 44.80 | 41.62 | 41.70 | 21.86 | 44.37 | 19.67 | 33.86 |
| LN02 | 30.13 | 18.52 | 29.69 | 21.66 | 40.18 | 59.82 | 44.97 | 41.62 | 41.18 | 21.56 | 44.85 | 20.11 | 33.95 |
| LN01 | 30.28 | 18.40 | 29.75 | 21.58 | 39.98 | 60.02 | 44.89 | 41.62 | 41.57 | 21.21 | 45.02 | 19.81 | 33.42 |
| L99 | 30.39 | 18.40 | 29.72 | 21.49 | 39.89 | 60.11 | 44.36 | 41.80 | 42.21 | 22.05 | 44.22 | 18.79 | 33.51 |
| KI-88-15 | 30.51 | 18.81 | 29.28 | 21.40 | 40.21 | 59.79 | 44.80 | 41.62 | 42.36 | 22.51 | 43.40 | 19.45 | 34.22 |
| KI-85-1 | 30.42 | 18.81 | 29.28 | 21.49 | 40.30 | 59.70 | 45.06 | 41.62 | 42.36 | 22.51 | 43.40 | 19.42 | 34.22 |
| KI-83-262 | 30.42 | 18.78 | 29.31 | 21.49 | 40.27 | 59.73 | 45.06 | 41.71 | 42.26 | 22.29 | 43.61 | 19.37 | 34.04 |
| JiangxiXinjianRn-07-2011 | 30.63 | 18.40 | 29.84 | 21.13 | 39.54 | 60.46 | 44.36 | 41.71 | 43.28 | 21.65 | 44.70 | 17.93 | 32.54 |
| IR473 | 30.57 | 18.81 | 29.34 | 21.28 | 40.09 | 59.91 | 44.89 | 41.62 | 42.61 | 22.35 | 43.63 | 18.90 | 33.77 |
| IR461 | 30.48 | 18.87 | 29.31 | 21.34 | 40.21 | 59.79 | 44.80 | 41.80 | 42.42 | 22.52 | 43.43 | 18.92 | 34.04 |
| IR162 | 30.51 | 18.81 | 29.34 | 21.34 | 40.15 | 59.85 | 44.89 | 41.62 | 42.36 | 22.35 | 43.63 | 19.17 | 33.95 |
| Humber | 30.81 | 18.64 | 29.54 | 21.02 | 39.65 | 60.35 | 45.24 | 41.62 | 43.84 | 21.41 | 44.65 | 17.49 | 32.10 |
| HuludaoRn101 | 30.42 | 18.64 | 29.57 | 21.37 | 40.01 | 59.99 | 44.44 | 41.62 | 42.07 | 22.38 | 43.89 | 19.15 | 33.95 |
| Hu02-258/NGS | 30.39 | 18.90 | 29.28 | 21.43 | 40.33 | 59.67 | 44.80 | 41.71 | 42.01 | 22.94 | 43.29 | 19.23 | 34.48 |
| HN4 | 30.28 | 18.55 | 29.66 | 21.52 | 40.06 | 59.94 | 44.80 | 41.62 | 41.82 | 21.86 | 44.37 | 19.51 | 33.77 |
| HN1 | 30.28 | 18.46 | 29.78 | 21.49 | 39.95 | 60.05 | 45.06 | 41.62 | 41.82 | 21.24 | 45.18 | 19.20 | 33.16 |
| HeB38 | 30.36 | 18.64 | 29.57 | 21.43 | 40.06 | 59.94 | 44.89 | 41.45 | 41.82 | 22.04 | 44.41 | 19.39 | 33.86 |
| HBT50 | 30.22 | 18.58 | 29.63 | 21.58 | 40.15 | 59.85 | 44.89 | 41.71 | 41.63 | 21.84 | 44.43 | 19.67 | 33.86 |
| HBT49 | 30.34 | 18.58 | 29.60 | 21.49 | 40.06 | 59.94 | 44.80 | 41.62 | 42.00 | 21.97 | 44.26 | 19.39 | 33.77 |
| HBT14 | 30.16 | 18.43 | 29.75 | 21.66 | 40.09 | 59.91 | 44.89 | 41.62 | 41.38 | 21.41 | 44.76 | 20.11 | 33.77 |
| HBT-3 | 30.25 | 18.61 | 29.69 | 21.46 | 40.06 | 59.94 | 44.89 | 41.62 | 41.68 | 21.86 | 44.70 | 19.42 | 33.69 |
| HBQ43 | 30.25 | 18.64 | 29.57 | 21.55 | 40.18 | 59.82 | 44.80 | 41.62 | 41.63 | 22.19 | 44.16 | 19.67 | 34.13 |
| HBQ20 | 30.16 | 18.52 | 29.72 | 21.60 | 40.12 | 59.88 | 44.89 | 41.62 | 41.26 | 21.65 | 44.81 | 19.92 | 33.86 |
| HBQ17 | 30.01 | 18.84 | 29.42 | 21.72 | 40.56 | 59.44 | 45.06 | 41.80 | 40.71 | 22.38 | 44.00 | 20.52 | 34.83 |
| HBQ7 | 30.25 | 18.61 | 29.57 | 21.58 | 40.18 | 59.82 | 44.89 | 41.62 | 41.63 | 21.97 | 44.26 | 19.81 | 34.04 |
| HBQ5 | 30.22 | 18.49 | 29.66 | 21.63 | 40.12 | 59.88 | 44.80 | 41.62 | 41.50 | 21.65 | 44.48 | 20.08 | 33.95 |
| HB55 | 30.36 | 18.55 | 29.60 | 21.49 | 40.04 | 59.96 | 44.62 | 41.62 | 42.19 | 22.29 | 43.94 | 19.15 | 33.86 |
| HaixingRn40 | 30.13 | 18.46 | 29.75 | 21.66 | 40.12 | 59.88 | 45.24 | 41.71 | 41.43 | 20.95 | 45.14 | 20.11 | 33.42 |
| GZRn148 | 30.19 | 18.49 | 29.69 | 21.63 | 40.12 | 59.88 | 44.80 | 41.71 | 41.52 | 21.65 | 44.48 | 19.89 | 33.86 |
| GZRn134 | 30.31 | 18.52 | 29.72 | 21.46 | 39.98 | 60.02 | 44.62 | 41.53 | 41.94 | 21.97 | 44.26 | 19.37 | 33.77 |
| GZRn133 | 30.31 | 18.49 | 29.72 | 21.49 | 39.98 | 60.02 | 44.62 | 41.53 | 42.00 | 21.97 | 44.26 | 19.39 | 33.77 |
| GZRn127 | 30.31 | 18.52 | 29.69 | 21.49 | 40.01 | 59.99 | 44.62 | 41.62 | 41.94 | 21.97 | 44.26 | 19.37 | 33.77 |
| GZRn110 | 30.19 | 18.46 | 29.72 | 21.63 | 40.09 | 59.91 | 44.80 | 41.71 | 41.52 | 21.54 | 44.59 | 19.89 | 33.77 |
| GZRn107 | 30.31 | 18.55 | 29.66 | 21.49 | 40.04 | 59.96 | 44.62 | 41.62 | 41.94 | 22.08 | 44.16 | 19.37 | 33.86 |
| GZRn100 | 30.25 | 18.69 | 29.45 | 21.60 | 40.30 | 59.70 | 44.62 | 41.71 | 41.65 | 22.51 | 43.51 | 19.92 | 34.57 |
| GZRn98 | 30.19 | 18.67 | 29.54 | 21.60 | 40.27 | 59.73 | 44.62 | 41.71 | 41.57 | 22.38 | 43.68 | 19.92 | 34.48 |
| GZRn96 | 30.28 | 18.52 | 29.69 | 21.52 | 40.04 | 59.96 | 44.62 | 41.62 | 41.82 | 21.97 | 44.26 | 19.51 | 33.86 |
| GZRn92 | 30.22 | 18.69 | 29.54 | 21.55 | 40.24 | 59.76 | 44.44 | 41.71 | 41.40 | 22.51 | 43.72 | 19.92 | 34.57 |
| GZRn84 | 30.28 | 18.69 | 29.51 | 21.52 | 40.21 | 59.79 | 44.97 | 41.62 | 41.70 | 22.19 | 44.16 | 19.53 | 34.04 |
| GZRn77 | 30.22 | 18.69 | 29.45 | 21.63 | 40.33 | 59.67 | 44.71 | 41.71 | 41.65 | 22.51 | 43.51 | 19.89 | 34.57 |
| GZRn76 | 30.28 | 18.69 | 29.51 | 21.52 | 40.21 | 59.79 | 44.97 | 41.62 | 41.70 | 22.19 | 44.16 | 19.53 | 34.04 |
| GZRn74 | 30.28 | 18.52 | 29.69 | 21.52 | 40.04 | 59.96 | 44.62 | 41.62 | 41.82 | 21.97 | 44.26 | 19.51 | 33.86 |
| GZRn73 | 30.19 | 18.69 | 29.48 | 21.63 | 40.33 | 59.67 | 44.71 | 41.71 | 41.70 | 22.51 | 43.51 | 19.89 | 34.57 |
| GZRn60 | 30.25 | 18.49 | 29.69 | 21.58 | 40.06 | 59.94 | 44.53 | 41.62 | 41.50 | 21.75 | 44.37 | 20.14 | 34.04 |
| GZRn54 | 30.22 | 18.40 | 29.75 | 21.63 | 40.04 | 59.96 | 44.62 | 41.36 | 41.45 | 21.73 | 44.22 | 20.36 | 34.13 |
| GZRn53 | 30.25 | 18.49 | 29.69 | 21.58 | 40.06 | 59.94 | 44.53 | 41.62 | 41.50 | 21.75 | 44.37 | 20.14 | 34.04 |
| GZRn51 | 30.25 | 18.49 | 29.69 | 21.58 | 40.06 | 59.94 | 44.62 | 41.53 | 41.55 | 21.75 | 44.37 | 20.14 | 34.04 |
| GZRn36 | 30.36 | 18.52 | 29.63 | 21.49 | 40.01 | 59.99 | 44.71 | 41.53 | 42.19 | 21.95 | 44.00 | 19.53 | 33.77 |
| Gou3 | 30.69 | 17.75 | 30.01 | 21.55 | 39.30 | 60.70 | 45.15 | 41.36 | 43.84 | 19.28 | 45.61 | 19.09 | 31.39 |
| GAW50/2021 | 30.78 | 18.28 | 29.75 | 21.19 | 39.48 | 60.52 | 45.24 | 41.53 | 45.01 | 21.04 | 44.12 | 17.10 | 31.66 |
| GAW30/2021 | 30.72 | 18.43 | 29.60 | 21.25 | 39.68 | 60.32 | 45.24 | 41.62 | 44.83 | 21.57 | 43.58 | 17.21 | 32.19 |
| Fj372/2013 | 30.45 | 18.05 | 30.07 | 21.43 | 39.48 | 60.52 | 44.89 | 41.62 | 42.44 | 19.70 | 46.21 | 19.26 | 31.92 |
| FJ36 | 30.19 | 18.67 | 29.69 | 21.46 | 40.12 | 59.88 | 44.97 | 41.53 | 41.26 | 21.99 | 44.85 | 19.56 | 33.86 |
| FJ35 | 30.19 | 18.67 | 29.69 | 21.46 | 40.12 | 59.88 | 44.97 | 41.53 | 41.26 | 21.99 | 44.85 | 19.56 | 33.86 |
| DPRK08 | 30.34 | 18.28 | 29.95 | 21.43 | 39.71 | 60.29 | 44.71 | 41.62 | 41.75 | 20.91 | 45.72 | 19.15 | 32.80 |
| DN2 | 30.39 | 18.34 | 29.69 | 21.58 | 39.92 | 60.08 | 44.18 | 41.98 | 42.01 | 21.30 | 44.32 | 19.94 | 33.60 |
| CVR/2019 | 30.54 | 18.55 | 29.72 | 21.19 | 39.74 | 60.26 | 45.24 | 41.80 | 43.00 | 21.30 | 45.19 | 17.67 | 32.19 |
| China | 30.28 | 18.46 | 29.72 | 21.55 | 40.01 | 59.99 | 44.80 | 41.45 | 41.30 | 21.45 | 44.75 | 20.36 | 33.77 |
| Cherwell | 30.54 | 18.58 | 29.66 | 21.22 | 39.80 | 60.20 | 45.15 | 41.80 | 42.87 | 21.41 | 44.97 | 17.95 | 32.45 |
| BjHD01 | 30.22 | 18.58 | 29.63 | 21.58 | 40.15 | 59.85 | 45.06 | 41.62 | 41.75 | 21.86 | 44.48 | 19.51 | 33.77 |
| B-1 | 30.39 | 18.64 | 29.51 | 21.46 | 40.09 | 59.91 | 45.33 | 41.53 | 42.24 | 21.75 | 44.48 | 18.98 | 33.42 |
| 201701555/SEOV/Utah_US/Rat | 30.51 | 18.46 | 29.81 | 21.22 | 39.68 | 60.32 | 45.15 | 41.71 | 42.75 | 20.97 | 45.41 | 18.11 | 32.19 |
| 201701554/SEOV/Utah_US/Rat | 30.54 | 18.46 | 29.81 | 21.19 | 39.65 | 60.35 | 45.15 | 41.71 | 42.87 | 20.97 | 45.41 | 17.97 | 32.10 |
| 201701321/SEOV/Colorado_US/Rat | 30.48 | 18.46 | 29.84 | 21.22 | 39.68 | 60.32 | 45.15 | 41.71 | 42.68 | 20.95 | 45.46 | 18.13 | 32.19 |
| 201701093/SEOV/Illinois_US/Rat | 30.54 | 18.46 | 29.81 | 21.19 | 39.65 | 60.35 | 45.15 | 41.71 | 42.87 | 20.97 | 45.41 | 17.97 | 32.10 |
| 80-39 | 30.07 | 18.93 | 29.31 | 21.69 | 40.62 | 59.38 | 44.89 | 41.71 | 40.84 | 22.94 | 43.40 | 20.33 | 35.27 |
| ZT71 | 30.28 | 18.40 | 29.69 | 21.63 | 40.04 | 59.96 | 44.97 | 41.62 | 41.65 | 21.13 | 44.85 | 20.08 | 33.51 |
| HBT65/2014 | 30.34 | 18.61 | 29.51 | 21.55 | 40.15 | 59.85 | 44.80 | 41.62 | 41.75 | 22.08 | 44.16 | 19.67 | 34.04 |
| HBT64/2014 | 30.28 | 18.67 | 29.54 | 21.52 | 40.18 | 59.82 | 44.97 | 41.62 | 41.75 | 22.08 | 44.26 | 19.53 | 33.95 |
| HBT63/2013 | 30.28 | 18.58 | 29.63 | 21.52 | 40.09 | 59.91 | 44.89 | 41.62 | 41.75 | 21.86 | 44.48 | 19.53 | 33.77 |
| HBT62/2014 | 30.34 | 18.61 | 29.63 | 21.43 | 40.04 | 59.96 | 44.89 | 41.62 | 42.05 | 21.97 | 44.48 | 19.15 | 33.60 |
| HBT60/2013 | 30.28 | 18.58 | 29.63 | 21.52 | 40.09 | 59.91 | 44.89 | 41.62 | 41.75 | 21.86 | 44.48 | 19.53 | 33.77 |
| HBT52/2013 | 30.22 | 18.55 | 29.63 | 21.60 | 40.15 | 59.85 | 44.80 | 41.62 | 41.38 | 21.75 | 44.48 | 20.11 | 34.04 |
| HBT43/2012 | 30.28 | 18.55 | 29.63 | 21.55 | 40.09 | 59.91 | 44.89 | 41.53 | 41.68 | 21.75 | 44.48 | 19.83 | 33.86 |
| HBT41/2012 | 30.19 | 18.55 | 29.66 | 21.60 | 40.15 | 59.85 | 44.80 | 41.62 | 41.26 | 21.75 | 44.59 | 20.11 | 34.04 |
| HBT7/2016 | 30.42 | 18.46 | 29.66 | 21.46 | 39.92 | 60.08 | 45.06 | 41.62 | 42.61 | 21.43 | 44.59 | 18.93 | 33.07 |
| HBT6/2015 | 30.34 | 18.58 | 29.57 | 21.52 | 40.09 | 59.91 | 44.89 | 41.62 | 41.87 | 21.86 | 44.37 | 19.53 | 33.77 |
| HBT5/2016 | 30.19 | 18.52 | 29.66 | 21.63 | 40.15 | 59.85 | 44.97 | 41.62 | 41.33 | 21.51 | 44.65 | 20.22 | 33.86 |
| HBQ75/2000 | 30.36 | 18.55 | 29.66 | 21.43 | 39.98 | 60.02 | 44.36 | 41.62 | 42.07 | 22.38 | 43.89 | 19.15 | 33.95 |
| HBQ74/2000 | 30.25 | 18.61 | 29.60 | 21.55 | 40.15 | 59.85 | 44.89 | 41.62 | 41.63 | 21.97 | 44.37 | 19.67 | 33.95 |
| HBQ73/2000 | 30.13 | 18.61 | 29.63 | 21.63 | 40.24 | 59.76 | 44.89 | 41.62 | 41.18 | 21.97 | 44.48 | 20.11 | 34.22 |
| HBQ72/2000 | 30.36 | 18.58 | 29.60 | 21.46 | 40.04 | 59.96 | 44.89 | 41.62 | 42.00 | 21.75 | 44.48 | 19.42 | 33.60 |
| HBQ65/2009 | 30.31 | 18.61 | 29.60 | 21.49 | 40.09 | 59.91 | 44.89 | 41.71 | 41.82 | 21.86 | 44.48 | 19.37 | 33.69 |
| HBQ64/2022 | 30.19 | 18.75 | 29.48 | 21.58 | 40.33 | 59.67 | 44.97 | 41.62 | 41.50 | 22.40 | 43.94 | 19.81 | 34.39 |
| HBQ63/2021 | 30.28 | 18.55 | 29.69 | 21.49 | 40.04 | 59.96 | 44.80 | 41.62 | 41.75 | 21.86 | 44.59 | 19.39 | 33.69 |
| HBQ63/2001 | 30.36 | 18.58 | 29.60 | 21.46 | 40.04 | 59.96 | 44.89 | 41.62 | 42.00 | 21.75 | 44.48 | 19.42 | 33.60 |
| HBQ62/2001 | 30.16 | 18.64 | 29.60 | 21.60 | 40.24 | 59.76 | 44.89 | 41.62 | 41.33 | 22.08 | 44.26 | 19.94 | 34.22 |
| HBQ60/2021 | 30.34 | 18.61 | 29.51 | 21.55 | 40.15 | 59.85 | 44.89 | 41.62 | 41.80 | 21.95 | 44.22 | 19.70 | 33.95 |
| HBQ59/2021 | 30.28 | 18.64 | 29.60 | 21.49 | 40.12 | 59.88 | 45.06 | 41.62 | 41.75 | 21.86 | 44.59 | 19.39 | 33.69 |
| HBQ58/2021 | 30.28 | 18.64 | 29.60 | 21.49 | 40.12 | 59.88 | 45.06 | 41.62 | 41.75 | 21.86 | 44.59 | 19.39 | 33.69 |
| HBQ57/2021 | 30.34 | 18.61 | 29.51 | 21.55 | 40.15 | 59.85 | 44.89 | 41.62 | 41.80 | 21.95 | 44.22 | 19.70 | 33.95 |
| HBQ50/2021 | 30.10 | 18.49 | 29.78 | 21.63 | 40.12 | 59.88 | 44.97 | 41.71 | 41.26 | 21.54 | 45.02 | 19.81 | 33.69 |
| HBQ49/2004 | 30.19 | 18.52 | 29.72 | 21.58 | 40.09 | 59.91 | 44.97 | 41.62 | 41.50 | 21.54 | 44.81 | 19.81 | 33.69 |
| HBQ47/2021 | 30.19 | 18.67 | 29.57 | 21.58 | 40.24 | 59.76 | 44.80 | 41.53 | 41.31 | 22.32 | 44.20 | 19.97 | 34.39 |
| HBQ24/2011 | 30.34 | 18.58 | 29.60 | 21.49 | 40.06 | 59.94 | 44.80 | 41.62 | 41.87 | 21.86 | 44.37 | 19.56 | 33.77 |
| HBQ15/2018 | 30.22 | 18.69 | 29.45 | 21.63 | 40.33 | 59.67 | 44.89 | 41.45 | 41.55 | 22.54 | 43.66 | 19.97 | 34.66 |
| HBQ7/2012 | 30.16 | 18.61 | 29.60 | 21.63 | 40.24 | 59.76 | 44.80 | 41.53 | 41.06 | 21.97 | 44.37 | 20.41 | 34.39 |
| HBQ5/2015 | 30.10 | 18.75 | 29.48 | 21.66 | 40.42 | 59.58 | 45.06 | 41.71 | 41.13 | 22.19 | 44.16 | 20.22 | 34.48 |
| HBQ4/2012 | 30.36 | 18.58 | 29.60 | 21.46 | 40.04 | 59.96 | 44.80 | 41.62 | 42.00 | 21.86 | 44.37 | 19.42 | 33.69 |
| HBQ2/2015 | 30.16 | 18.61 | 29.60 | 21.63 | 40.24 | 59.76 | 44.97 | 41.62 | 41.26 | 21.86 | 44.48 | 20.08 | 34.13 |
| HBQ1/2015 | 30.22 | 18.52 | 29.72 | 21.55 | 40.06 | 59.94 | 44.80 | 41.62 | 41.45 | 21.62 | 44.65 | 19.92 | 33.77 |
| HBL131/2007 | 30.19 | 18.64 | 29.57 | 21.60 | 40.24 | 59.76 | 44.80 | 41.62 | 41.26 | 22.08 | 44.26 | 20.08 | 34.30 |
| HBL3/2005 | 30.16 | 18.37 | 29.81 | 21.66 | 40.04 | 59.96 | 45.15 | 41.62 | 41.38 | 20.78 | 45.35 | 20.22 | 33.33 |
| HBH51/2021 | 30.22 | 18.46 | 29.69 | 21.63 | 40.09 | 59.91 | 45.24 | 41.53 | 41.55 | 21.00 | 45.02 | 20.25 | 33.51 |
| HBCZ88/1999 | 30.31 | 18.40 | 29.78 | 21.52 | 39.92 | 60.08 | 45.24 | 41.62 | 42.00 | 20.89 | 45.35 | 19.39 | 32.89 |
| HBCD56/2002 | 30.19 | 18.61 | 29.63 | 21.58 | 40.18 | 59.82 | 44.89 | 41.62 | 41.38 | 21.86 | 44.48 | 19.97 | 34.04 |
| HBCD55/2021 | 30.19 | 18.69 | 29.54 | 21.58 | 40.27 | 59.73 | 45.06 | 41.62 | 41.50 | 22.08 | 44.26 | 19.81 | 34.13 |
| HBCD55/2002 | 30.51 | 18.31 | 29.95 | 21.22 | 39.54 | 60.46 | 44.44 | 41.80 | 42.86 | 21.10 | 45.24 | 18.29 | 32.36 |
| HBCD52/2002 | 30.16 | 18.64 | 29.63 | 21.58 | 40.21 | 59.79 | 44.97 | 41.62 | 41.38 | 21.86 | 44.48 | 19.94 | 34.04 |
| HBCD44/2021 | 30.13 | 18.72 | 29.51 | 21.63 | 40.36 | 59.64 | 45.06 | 41.62 | 41.26 | 22.19 | 44.16 | 20.08 | 34.39 |
| HBCD13/2019 | 30.22 | 18.72 | 29.51 | 21.55 | 40.27 | 59.73 | 45.06 | 41.53 | 41.63 | 22.29 | 44.05 | 19.67 | 34.22 |
| HBCD9/2017 | 30.22 | 18.72 | 29.54 | 21.52 | 40.24 | 59.76 | 44.97 | 41.62 | 41.63 | 22.19 | 44.16 | 19.67 | 34.13 |
| HBCD8/2017 | 30.22 | 18.69 | 29.54 | 21.55 | 40.24 | 59.76 | 45.06 | 41.62 | 41.63 | 22.08 | 44.26 | 19.67 | 34.04 |
| HBCD6/2017 | 30.13 | 18.69 | 29.54 | 21.63 | 40.33 | 59.67 | 45.06 | 41.62 | 41.26 | 22.08 | 44.26 | 20.08 | 34.30 |
| HBCD4/2017 | 30.16 | 18.67 | 29.57 | 21.60 | 40.27 | 59.73 | 45.06 | 41.62 | 41.38 | 21.97 | 44.37 | 19.94 | 34.13 |
| HBCD3/2017 | 30.22 | 18.72 | 29.51 | 21.55 | 40.27 | 59.73 | 45.06 | 41.62 | 41.63 | 22.19 | 44.16 | 19.67 | 34.13 |
| HBCD1/2017 | 30.16 | 18.67 | 29.54 | 21.63 | 40.30 | 59.70 | 45.06 | 41.62 | 41.38 | 21.97 | 44.26 | 20.08 | 34.22 |
| HBB36/2002 | 30.42 | 18.58 | 29.57 | 21.43 | 40.01 | 59.99 | 44.27 | 41.62 | 42.19 | 22.49 | 43.57 | 19.28 | 34.13 |
| HBB35/2002 | 30.45 | 18.55 | 29.63 | 21.37 | 39.92 | 60.08 | 44.09 | 41.62 | 42.07 | 22.38 | 43.78 | 19.34 | 34.04 |
| 93HBX12 | 30.31 | 18.64 | 29.57 | 21.49 | 40.12 | 59.88 | 44.89 | 41.45 | 41.57 | 22.04 | 44.41 | 19.67 | 34.04 |
| 93HBX11 | 30.31 | 18.67 | 29.54 | 21.49 | 40.15 | 59.85 | 44.80 | 41.62 | 41.75 | 22.02 | 44.25 | 19.72 | 34.04 |
| 93HBX10 | 30.34 | 18.72 | 29.60 | 21.34 | 40.06 | 59.94 | 45.24 | 41.71 | 41.94 | 21.89 | 44.96 | 18.68 | 33.25 |
| 93HBQ4 | 30.36 | 18.58 | 29.69 | 21.37 | 39.95 | 60.05 | 44.62 | 41.53 | 41.92 | 21.78 | 44.53 | 19.48 | 33.69 |
| 93HBQ3 | 30.36 | 18.64 | 29.57 | 21.43 | 40.06 | 59.94 | 44.71 | 41.36 | 41.75 | 22.15 | 44.19 | 19.72 | 34.13 |
| 93HBJ20 | 30.31 | 18.64 | 29.57 | 21.49 | 40.12 | 59.88 | 44.89 | 41.45 | 41.57 | 22.04 | 44.41 | 19.67 | 34.04 |
|  |  |  |  |  |  |  |  |  |  |  |  |  |  |
| Mean | 30.31 | 18.58 | 29.61 | 21.50 | 40. 08 | 59.92 | 44.87 | 41.65 | 41.89 | 21. 82 | 44.41 | 19.47 | 33.71 |
| Standard deviation | 0.16 | 0.16 | 0.16 | 0.16 | 0.25 | 0.25 | 0.25 | 0.12 | 0.67 | 0.56 | 0.58 | 0.75 | 0.77 |

| Table S9. The nucleotide composition of S segment (%) | | | | | | | | | | | | | |
| --- | --- | --- | --- | --- | --- | --- | --- | --- | --- | --- | --- | --- | --- |
| **SEQUENCES \ PARAMETERS** | **A** | **C** | **U** | **G** | **GC** | **AU** | **GC1** | **GC2** | **A3** | **C3** | **U3** | **G3** | **GC3** |
| ZT71 | 31.40 | 19.92 | 22.48 | 26.20 | 46.12 | 53.88 | 53.26 | 39.77 | 38.53 | 23.38 | 33.23 | 32.21 | 45.35 |
| ZT10 | 31.24 | 19.92 | 22.40 | 26.43 | 46.36 | 53.64 | 53.49 | 40.23 | 38.53 | 23.31 | 33.13 | 32.11 | 45.35 |
| ZJ5 | 30.62 | 19.77 | 23.49 | 26.12 | 45.89 | 54.11 | 53.95 | 39.77 | 36.97 | 21.28 | 35.87 | 32.67 | 43.95 |
| Z37 | 31.40 | 19.92 | 22.48 | 26.20 | 46.12 | 53.88 | 53.02 | 40.23 | 38.84 | 23.24 | 33.03 | 31.88 | 45.12 |
| YZG-Changchun | 31.40 | 19.69 | 22.71 | 26.20 | 45.89 | 54.11 | 53.02 | 40.00 | 38.72 | 22.02 | 33.64 | 32.44 | 44.65 |
| YY27 | 31.16 | 19.84 | 22.71 | 26.28 | 46.12 | 53.88 | 52.79 | 40.00 | 37.80 | 22.94 | 33.33 | 32.78 | 45.58 |
| YongjiaRn14 | 30.70 | 20.16 | 23.02 | 26.12 | 46.28 | 53.72 | 53.49 | 40.00 | 37.20 | 23.24 | 34.25 | 32.11 | 45.35 |
| YongjiaRf45 | 30.62 | 20.16 | 23.02 | 26.20 | 46.36 | 53.64 | 53.49 | 40.00 | 36.89 | 23.24 | 34.25 | 32.44 | 45.58 |
| YN45 | 31.24 | 19.84 | 22.79 | 26.12 | 45.97 | 54.03 | 52.79 | 40.00 | 38.11 | 22.94 | 33.64 | 32.11 | 45.12 |
| YaluRiver12 | 30.78 | 20.31 | 22.09 | 26.82 | 47.13 | 52.87 | 53.02 | 40.23 | 36.06 | 24.54 | 31.60 | 34.55 | 48.14 |
| XM47 | 31.24 | 19.77 | 22.95 | 26.05 | 45.81 | 54.19 | 52.79 | 40.00 | 38.23 | 22.56 | 34.15 | 31.88 | 44.65 |
| XJ5/2011 | 31.32 | 19.53 | 22.95 | 26.20 | 45.74 | 54.26 | 52.33 | 40.00 | 38.11 | 22.02 | 33.94 | 32.78 | 44.88 |
| XJ2/2011 | 31.24 | 19.46 | 23.02 | 26.28 | 45.74 | 54.26 | 52.09 | 40.47 | 38.41 | 21.95 | 33.84 | 32.44 | 44.65 |
| XiaotangshanRn7 | 31.32 | 19.84 | 22.79 | 26.05 | 45.89 | 54.11 | 53.02 | 40.23 | 38.60 | 22.63 | 33.94 | 31.33 | 44.42 |
| WuhanRn98 | 31.01 | 19.53 | 23.02 | 26.43 | 45.97 | 54.03 | 52.56 | 40.00 | 37.20 | 22.02 | 34.25 | 33.44 | 45.35 |
| WuhanRn75 | 31.09 | 19.53 | 23.02 | 26.36 | 45.89 | 54.11 | 52.56 | 40.00 | 37.50 | 22.02 | 34.25 | 33.11 | 45.12 |
| WuhanRn67u | 31.09 | 19.53 | 22.95 | 26.43 | 45.97 | 54.03 | 52.56 | 40.00 | 37.50 | 22.02 | 33.94 | 33.44 | 45.35 |
| WuhanRn63 | 31.01 | 19.53 | 23.02 | 26.43 | 45.97 | 54.03 | 52.56 | 40.00 | 37.20 | 22.02 | 34.25 | 33.44 | 45.35 |
| WuhanRn53 | 31.09 | 19.53 | 22.95 | 26.43 | 45.97 | 54.03 | 52.56 | 40.00 | 37.50 | 22.02 | 33.94 | 33.44 | 45.35 |
| WuhanRn25 | 31.09 | 19.53 | 23.02 | 26.36 | 45.89 | 54.11 | 52.56 | 40.00 | 37.50 | 22.02 | 34.25 | 33.11 | 45.12 |
| WuhanRn10 | 31.01 | 19.53 | 23.10 | 26.36 | 45.89 | 54.11 | 52.56 | 40.00 | 37.20 | 22.02 | 34.56 | 33.11 | 45.12 |
| WuhanRf49 | 31.01 | 19.53 | 23.02 | 26.43 | 45.97 | 54.03 | 52.56 | 40.00 | 37.20 | 22.02 | 34.25 | 33.44 | 45.35 |
| WuhanRf33 | 31.16 | 19.61 | 22.95 | 26.28 | 45.89 | 54.11 | 52.56 | 40.00 | 37.80 | 22.32 | 33.94 | 32.78 | 45.12 |
| WuhanRf18 | 31.16 | 19.53 | 22.95 | 26.36 | 45.89 | 54.11 | 52.56 | 40.00 | 37.80 | 22.02 | 33.94 | 33.11 | 45.12 |
| WuhanRf12 | 31.09 | 19.46 | 23.02 | 26.43 | 45.89 | 54.11 | 52.56 | 40.00 | 37.50 | 22.02 | 34.25 | 33.11 | 45.12 |
| WuhanRf08 | 31.09 | 19.38 | 23.02 | 26.51 | 45.89 | 54.11 | 52.56 | 40.00 | 37.50 | 21.71 | 34.25 | 33.44 | 45.12 |
| WuhanRf07 | 31.16 | 19.46 | 23.02 | 26.36 | 45.81 | 54.19 | 52.56 | 40.00 | 37.80 | 22.02 | 34.25 | 32.78 | 44.88 |
| WuhanRf02 | 30.85 | 19.69 | 22.71 | 26.74 | 46.43 | 53.57 | 53.26 | 40.00 | 37.20 | 22.63 | 33.33 | 33.78 | 46.05 |
| WuhanMm24 | 31.01 | 19.69 | 22.79 | 26.51 | 46.20 | 53.80 | 52.56 | 40.47 | 37.50 | 22.26 | 33.54 | 33.44 | 45.58 |
| WuhanMm13 | 31.09 | 19.53 | 22.95 | 26.43 | 45.97 | 54.03 | 52.56 | 40.00 | 37.50 | 22.02 | 33.94 | 33.44 | 45.35 |
| TURCKHEIM/Rn/FRA/2016/2016.00033 | 31.63 | 19.69 | 22.79 | 25.89 | 45.58 | 54.42 | 52.79 | 39.77 | 39.33 | 22.26 | 33.54 | 31.88 | 44.19 |
| TURCKHEIM/Hu/FRA/2016/2016.00044 | 31.63 | 19.69 | 22.79 | 25.89 | 45.58 | 54.42 | 52.79 | 39.77 | 39.33 | 22.26 | 33.54 | 31.88 | 44.19 |
| TGN07/2019 | 31.16 | 19.84 | 23.33 | 25.66 | 45.50 | 54.50 | 52.79 | 40.00 | 38.72 | 22.63 | 34.86 | 30.54 | 43.72 |
| TGN07/2018 | 30.85 | 19.61 | 23.57 | 25.97 | 45.58 | 54.42 | 52.79 | 40.00 | 37.80 | 22.02 | 35.47 | 31.44 | 43.95 |
| Tchoupitoulas/POR | 31.32 | 19.53 | 22.95 | 26.20 | 45.74 | 54.26 | 53.02 | 40.00 | 38.53 | 21.41 | 34.56 | 32.55 | 44.19 |
| TchoupitoulasTCH | 31.24 | 19.53 | 23.02 | 26.20 | 45.74 | 54.26 | 53.02 | 40.00 | 38.53 | 21.41 | 34.56 | 32.55 | 44.19 |
| Taonan420 | 30.85 | 20.16 | 23.18 | 25.81 | 45.97 | 54.03 | 53.26 | 40.23 | 37.92 | 22.19 | 34.65 | 32.21 | 44.42 |
| Taonan52 | 30.78 | 20.47 | 22.40 | 26.36 | 46.82 | 53.18 | 53.95 | 41.40 | 37.76 | 22.73 | 33.33 | 31.80 | 45.12 |
| SZ148 | 31.32 | 19.84 | 22.79 | 26.05 | 45.89 | 54.11 | 53.02 | 40.00 | 38.72 | 22.63 | 33.64 | 31.77 | 44.65 |
| SZ54 | 31.16 | 19.69 | 22.95 | 26.20 | 45.89 | 54.11 | 52.79 | 40.00 | 37.80 | 22.32 | 34.25 | 32.44 | 44.88 |
| SR-11 | 31.55 | 20.00 | 22.40 | 26.05 | 46.05 | 53.95 | 53.02 | 40.00 | 39.63 | 23.24 | 32.11 | 31.77 | 45.12 |
| SOV/Rn19-5 | 31.24 | 19.69 | 22.95 | 26.12 | 45.81 | 54.19 | 53.02 | 40.23 | 38.30 | 21.71 | 34.56 | 32.00 | 44.19 |
| SOV/Rn18-1 | 31.55 | 19.30 | 22.95 | 26.20 | 45.50 | 54.50 | 52.33 | 40.23 | 39.33 | 21.34 | 33.84 | 32.11 | 43.95 |
| Singapore/06RN46 | 31.32 | 19.77 | 22.64 | 26.28 | 46.05 | 53.95 | 52.56 | 40.00 | 38.72 | 23.24 | 32.42 | 32.44 | 45.58 |
| ShenyangRn139 | 31.47 | 20.08 | 22.33 | 26.12 | 46.20 | 53.80 | 53.02 | 40.00 | 39.02 | 23.85 | 32.11 | 31.77 | 45.58 |
| ShenyangRn19 | 31.55 | 19.92 | 22.48 | 26.05 | 45.97 | 54.03 | 53.02 | 40.00 | 39.33 | 23.24 | 32.72 | 31.44 | 44.88 |
| SG42/2011 | 30.47 | 19.38 | 24.03 | 26.12 | 45.50 | 54.50 | 53.49 | 40.23 | 36.59 | 19.88 | 38.23 | 32.11 | 42.79 |
| Seoul-Baxter/NYC-D23 | 31.40 | 19.53 | 22.95 | 26.12 | 45.66 | 54.34 | 53.02 | 40.00 | 38.72 | 21.41 | 34.56 | 32.11 | 43.95 |
| SEO/Belgium/Rn895/2005 | 31.09 | 19.69 | 22.95 | 26.28 | 45.97 | 54.03 | 52.09 | 40.00 | 37.61 | 23.17 | 33.23 | 32.89 | 45.81 |
| SD201 | 31.55 | 19.92 | 22.56 | 25.97 | 45.89 | 54.11 | 53.02 | 40.00 | 39.33 | 23.24 | 33.03 | 31.10 | 44.65 |
| SC106 | 31.24 | 19.84 | 22.79 | 26.12 | 45.97 | 54.03 | 53.26 | 40.00 | 38.11 | 22.63 | 34.25 | 31.77 | 44.65 |
| RuianRr57 | 31.55 | 20.08 | 22.33 | 26.05 | 46.12 | 53.88 | 53.02 | 40.00 | 39.33 | 23.85 | 32.11 | 31.44 | 45.35 |
| RuianRn242 | 31.47 | 19.77 | 22.79 | 25.97 | 45.74 | 54.26 | 53.02 | 40.00 | 39.02 | 22.63 | 33.94 | 31.10 | 44.19 |
| RuianRn180 | 31.47 | 19.77 | 22.71 | 26.05 | 45.81 | 54.19 | 53.02 | 40.00 | 39.02 | 22.63 | 33.64 | 31.44 | 44.42 |
| RuianRn76 | 31.40 | 20.16 | 22.25 | 26.20 | 46.36 | 53.64 | 53.02 | 40.00 | 38.72 | 24.16 | 31.80 | 32.11 | 46.05 |
| RuianRn33 | 31.47 | 20.39 | 22.87 | 25.27 | 45.66 | 54.34 | 53.26 | 39.30 | 39.51 | 23.48 | 32.93 | 30.23 | 44.42 |
| RuianRn23 | 31.47 | 20.08 | 22.25 | 26.20 | 46.28 | 53.72 | 53.02 | 40.00 | 38.60 | 23.85 | 32.11 | 32.33 | 45.81 |
| RuianRf74 | 31.55 | 20.00 | 22.33 | 26.12 | 46.12 | 53.88 | 53.02 | 39.77 | 39.33 | 23.85 | 31.80 | 31.88 | 45.58 |
| Rn-YUE12 | 31.55 | 20.00 | 22.40 | 26.05 | 46.05 | 53.95 | 53.02 | 40.23 | 39.51 | 23.55 | 32.42 | 31.00 | 44.88 |
| Rn-SHY17 | 31.24 | 19.77 | 22.79 | 26.20 | 45.97 | 54.03 | 53.02 | 40.23 | 38.30 | 22.32 | 33.94 | 32.00 | 44.65 |
| Rn-M11 | 31.55 | 19.84 | 22.56 | 26.05 | 45.89 | 54.11 | 52.79 | 40.47 | 39.51 | 22.87 | 32.93 | 31.00 | 44.42 |
| Rn-HD11 | 31.71 | 19.84 | 22.56 | 25.89 | 45.74 | 54.26 | 53.02 | 40.00 | 39.94 | 22.94 | 33.03 | 30.77 | 44.19 |
| Rn-DH27 | 31.24 | 20.08 | 22.25 | 26.43 | 46.51 | 53.49 | 53.02 | 40.00 | 38.11 | 23.85 | 31.80 | 33.11 | 46.51 |
| Rn-DC8 | 31.40 | 19.84 | 22.56 | 26.20 | 46.05 | 53.95 | 53.26 | 40.23 | 39.21 | 22.94 | 33.03 | 31.33 | 44.65 |
| Rn-CP7 | 31.55 | 20.00 | 22.40 | 26.05 | 46.05 | 53.95 | 53.02 | 40.23 | 39.51 | 23.55 | 32.42 | 31.00 | 44.88 |
| Rn11-53/NGS | 31.32 | 19.84 | 22.56 | 26.28 | 46.12 | 53.88 | 53.26 | 40.00 | 38.41 | 22.32 | 33.33 | 32.78 | 45.12 |
| Rn11-44/NGS | 31.32 | 19.46 | 22.95 | 26.28 | 45.74 | 54.26 | 52.79 | 40.00 | 38.41 | 21.41 | 34.25 | 32.78 | 44.42 |
| Rn10-145/NGS | 31.55 | 19.69 | 22.71 | 26.05 | 45.74 | 54.26 | 53.26 | 40.00 | 39.33 | 21.71 | 33.94 | 31.77 | 43.95 |
| Rn10-134/NGS | 31.47 | 19.69 | 22.71 | 26.12 | 45.81 | 54.19 | 53.26 | 40.00 | 39.02 | 21.71 | 33.94 | 32.11 | 44.19 |
| Rn10-145 | 31.16 | 20.08 | 22.40 | 26.36 | 46.43 | 53.57 | 53.26 | 40.00 | 37.80 | 22.94 | 32.72 | 33.44 | 46.05 |
| REPLONGES/Hu/FRA/2012/12-0882 | 31.47 | 19.69 | 22.64 | 26.20 | 45.89 | 54.11 | 52.79 | 40.00 | 39.02 | 22.32 | 33.03 | 32.44 | 44.88 |
| R22 | 31.78 | 19.53 | 22.87 | 25.81 | 45.35 | 54.65 | 52.79 | 39.77 | 39.63 | 21.78 | 34.36 | 31.10 | 43.49 |
| QixianRn10 | 31.47 | 19.61 | 22.79 | 26.12 | 45.74 | 54.26 | 53.02 | 40.00 | 39.02 | 22.02 | 33.94 | 31.77 | 44.19 |
| QingdaoMm15 | 31.71 | 19.53 | 22.87 | 25.89 | 45.43 | 54.57 | 53.02 | 40.00 | 39.94 | 21.71 | 34.25 | 30.77 | 43.26 |
| OuhaiRn251 | 31.71 | 19.46 | 22.87 | 25.97 | 45.43 | 54.57 | 52.79 | 40.00 | 39.63 | 21.71 | 34.25 | 31.10 | 43.49 |
| OuhaiRn189 | 31.32 | 19.69 | 22.71 | 26.28 | 45.97 | 54.03 | 53.02 | 40.00 | 38.72 | 22.94 | 33.33 | 31.77 | 44.88 |
| OuhaiRn146 | 31.40 | 19.77 | 22.64 | 26.20 | 45.97 | 54.03 | 53.02 | 40.00 | 39.02 | 23.24 | 33.03 | 31.44 | 44.88 |
| OuhaiRf35 | 31.55 | 20.16 | 22.17 | 26.12 | 46.28 | 53.72 | 52.79 | 40.00 | 39.33 | 24.77 | 31.19 | 31.44 | 46.05 |
| MM23 | 31.32 | 19.77 | 22.79 | 26.12 | 45.89 | 54.11 | 52.79 | 40.00 | 38.41 | 22.63 | 33.64 | 32.11 | 44.88 |
| MANTENAY-MONTLIN/Rn/FRA/2015/2015.00179 | 31.24 | 19.61 | 22.71 | 26.43 | 46.05 | 53.95 | 52.56 | 40.00 | 38.11 | 22.32 | 33.03 | 33.44 | 45.58 |
| LYON/Rn/FRA/2013/LYO852 | 30.85 | 19.69 | 22.79 | 26.67 | 46.36 | 53.64 | 52.33 | 40.00 | 36.59 | 22.94 | 33.03 | 34.45 | 46.74 |
| Longwan581 | 31.24 | 20.08 | 22.40 | 26.28 | 46.36 | 53.64 | 53.26 | 40.00 | 38.11 | 23.55 | 32.72 | 32.44 | 45.81 |
| LN06 | 31.24 | 19.69 | 22.95 | 26.12 | 45.81 | 54.19 | 53.02 | 40.23 | 38.30 | 22.02 | 34.56 | 31.67 | 44.19 |
| LN05 | 31.55 | 19.77 | 22.56 | 26.12 | 45.89 | 54.11 | 52.56 | 40.00 | 39.02 | 22.94 | 32.72 | 32.11 | 45.12 |
| LN04 | 31.47 | 19.84 | 22.56 | 26.12 | 45.97 | 54.03 | 52.79 | 40.00 | 39.02 | 23.24 | 32.72 | 31.77 | 45.12 |
| LN03 | 31.47 | 20.08 | 22.33 | 26.12 | 46.20 | 53.80 | 52.79 | 40.00 | 39.02 | 24.16 | 31.80 | 31.77 | 45.81 |
| LN02 | 31.71 | 20.00 | 22.40 | 25.89 | 45.89 | 54.11 | 53.02 | 40.00 | 39.94 | 23.55 | 32.42 | 30.77 | 44.65 |
| LN01 | 31.24 | 19.92 | 22.48 | 26.36 | 46.28 | 53.72 | 53.26 | 40.00 | 38.11 | 22.94 | 33.03 | 32.78 | 45.58 |
| L99 | 31.63 | 19.61 | 22.87 | 25.89 | 45.50 | 54.50 | 52.56 | 40.00 | 39.33 | 22.02 | 33.94 | 31.44 | 43.95 |
| JUN5-14 | 31.24 | 19.92 | 22.64 | 26.20 | 46.12 | 53.88 | 53.02 | 40.00 | 38.53 | 23.24 | 33.03 | 32.11 | 45.35 |
| JiningCt13 | 31.71 | 19.46 | 22.95 | 25.89 | 45.35 | 54.65 | 53.02 | 40.00 | 39.94 | 21.41 | 34.56 | 30.77 | 43.02 |
| JinanRn1 | 31.63 | 19.38 | 23.02 | 25.97 | 45.35 | 54.65 | 53.02 | 40.00 | 39.63 | 21.10 | 34.86 | 31.10 | 43.02 |
| JiangxiXinjianRn-09-2011 | 31.24 | 19.38 | 23.10 | 26.28 | 45.66 | 54.34 | 52.33 | 39.77 | 38.23 | 22.02 | 33.94 | 32.89 | 44.88 |
| JiangxiXinjianRn-07-2011 | 31.32 | 19.61 | 22.87 | 26.20 | 45.81 | 54.19 | 52.33 | 40.00 | 38.41 | 22.63 | 33.33 | 32.44 | 45.12 |
| IR473 | 31.55 | 19.46 | 23.02 | 25.97 | 45.43 | 54.57 | 52.33 | 39.53 | 38.84 | 22.02 | 33.94 | 32.21 | 44.42 |
| IR461 | 31.55 | 19.46 | 23.02 | 25.97 | 45.43 | 54.57 | 52.33 | 39.53 | 38.72 | 21.95 | 33.84 | 32.55 | 44.42 |
| IR162 | 31.55 | 19.46 | 23.02 | 25.97 | 45.43 | 54.57 | 52.33 | 39.53 | 38.84 | 22.02 | 33.94 | 32.21 | 44.42 |
| Humber | 31.55 | 19.84 | 22.40 | 26.20 | 46.05 | 53.95 | 53.02 | 40.00 | 39.33 | 22.63 | 32.42 | 32.44 | 45.12 |
| HuBJ22 | 31.16 | 19.92 | 22.71 | 26.20 | 46.12 | 53.88 | 53.02 | 40.23 | 37.99 | 22.94 | 33.64 | 32.00 | 45.12 |
| HuBJ20 | 31.01 | 19.77 | 22.79 | 26.43 | 46.20 | 53.80 | 53.02 | 40.00 | 37.20 | 22.63 | 33.94 | 33.11 | 45.58 |
| HuBJ19 | 31.40 | 19.61 | 23.02 | 25.97 | 45.58 | 54.42 | 52.79 | 40.23 | 38.91 | 22.02 | 34.56 | 31.00 | 43.72 |
| HuBJ16 | 31.40 | 19.61 | 23.10 | 25.89 | 45.50 | 54.50 | 52.79 | 40.23 | 38.60 | 21.71 | 35.17 | 31.00 | 43.49 |
| HuBJ15 | 31.40 | 19.61 | 22.87 | 26.12 | 45.74 | 54.26 | 52.79 | 40.00 | 38.41 | 22.32 | 34.25 | 31.77 | 44.42 |
| HuBJ9 | 31.63 | 19.92 | 22.48 | 25.97 | 45.89 | 54.11 | 53.02 | 40.00 | 39.63 | 23.24 | 32.72 | 31.10 | 44.65 |
| HuBJ7 | 31.16 | 20.00 | 22.64 | 26.20 | 46.20 | 53.80 | 53.26 | 40.23 | 37.99 | 22.94 | 33.64 | 32.00 | 45.12 |
| HuBJ3 | 31.24 | 20.08 | 22.40 | 26.28 | 46.36 | 53.64 | 53.26 | 40.23 | 38.79 | 23.85 | 32.11 | 31.56 | 45.58 |
| Hu02-529/NGS | 31.32 | 19.61 | 22.79 | 26.28 | 45.89 | 54.11 | 53.02 | 40.00 | 38.41 | 21.71 | 33.94 | 32.78 | 44.65 |
| Hu02-294/NGS | 31.40 | 19.69 | 22.71 | 26.20 | 45.89 | 54.11 | 53.02 | 40.00 | 38.72 | 22.02 | 33.64 | 32.44 | 44.65 |
| Hu02-258/NGS | 31.47 | 19.84 | 22.56 | 26.12 | 45.97 | 54.03 | 53.49 | 40.00 | 39.02 | 22.02 | 33.64 | 32.11 | 44.42 |
| HN4 | 31.09 | 19.69 | 22.95 | 26.28 | 45.97 | 54.03 | 52.79 | 40.00 | 37.50 | 22.32 | 34.25 | 32.78 | 45.12 |
| HN1 | 31.09 | 19.84 | 22.79 | 26.28 | 46.12 | 53.88 | 52.79 | 40.00 | 37.50 | 22.94 | 33.64 | 32.78 | 45.58 |
| HebeiRn9 | 31.71 | 19.46 | 22.95 | 25.89 | 45.35 | 54.65 | 53.02 | 40.00 | 39.94 | 21.41 | 34.56 | 30.77 | 43.02 |
| HebeiMm7 | 31.47 | 19.46 | 22.95 | 26.12 | 45.58 | 54.42 | 52.79 | 40.00 | 39.02 | 21.71 | 34.25 | 31.77 | 43.95 |
| HeB38 | 31.32 | 20.16 | 22.40 | 26.12 | 46.28 | 53.72 | 53.49 | 39.77 | 38.41 | 23.62 | 32.82 | 32.11 | 45.58 |
| Hb8610 | 31.40 | 19.77 | 22.71 | 26.12 | 45.89 | 54.11 | 52.79 | 40.23 | 38.60 | 22.63 | 33.64 | 31.67 | 44.65 |
| GZ488 | 30.93 | 19.77 | 22.87 | 26.43 | 46.20 | 53.80 | 52.79 | 40.00 | 36.89 | 22.63 | 33.94 | 33.44 | 45.81 |
| GZ473 | 31.01 | 19.77 | 22.87 | 26.36 | 46.12 | 53.88 | 52.79 | 40.00 | 37.20 | 22.63 | 33.94 | 33.11 | 45.58 |
| GZ325 | 31.24 | 19.92 | 22.79 | 26.05 | 45.97 | 54.03 | 52.79 | 40.00 | 38.23 | 23.17 | 33.54 | 31.88 | 45.12 |
| GZ45 | 31.09 | 20.08 | 22.56 | 26.28 | 46.36 | 53.64 | 52.79 | 40.00 | 37.50 | 23.85 | 32.72 | 32.78 | 46.28 |
| GZ15 | 31.09 | 19.77 | 22.87 | 26.28 | 46.05 | 53.95 | 52.79 | 40.00 | 37.50 | 22.63 | 33.94 | 32.78 | 45.35 |
| GuangzhouRn36 | 31.16 | 19.61 | 23.02 | 26.20 | 45.81 | 54.19 | 52.79 | 40.00 | 37.80 | 22.02 | 34.56 | 32.44 | 44.65 |
| Gou3 | 30.16 | 20.16 | 23.02 | 26.67 | 46.82 | 53.18 | 54.42 | 40.00 | 35.89 | 22.19 | 34.65 | 34.34 | 46.05 |
| Gongzhuling415 | 31.09 | 20.00 | 23.10 | 25.81 | 45.81 | 54.19 | 53.49 | 39.53 | 38.04 | 22.36 | 34.44 | 31.53 | 44.42 |
| Gongzhuling147 | 30.78 | 20.47 | 23.18 | 25.58 | 46.05 | 53.95 | 53.49 | 40.23 | 37.42 | 22.99 | 34.63 | 31.29 | 44.42 |
| Gongzhuling108 | 30.70 | 20.08 | 23.10 | 26.12 | 46.20 | 53.80 | 53.72 | 40.00 | 36.56 | 22.49 | 34.95 | 32.34 | 44.88 |
| Gongzhuling97 | 30.62 | 20.08 | 23.33 | 25.97 | 46.05 | 53.95 | 53.49 | 40.23 | 37.65 | 21.99 | 34.94 | 31.53 | 44.42 |
| Gongzhuling85 | 30.70 | 20.39 | 23.18 | 25.74 | 46.12 | 53.88 | 53.72 | 40.23 | 37.92 | 22.29 | 34.34 | 31.31 | 44.42 |
| Gongzhuling58 | 30.93 | 20.16 | 23.02 | 25.89 | 46.05 | 53.95 | 53.49 | 40.23 | 38.15 | 22.29 | 34.34 | 31.53 | 44.42 |
| Gongzhuling45 | 30.78 | 20.39 | 23.10 | 25.74 | 46.12 | 53.88 | 53.72 | 40.23 | 38.27 | 22.22 | 34.23 | 31.63 | 44.42 |
| Gongzhuling42 | 31.32 | 19.84 | 22.48 | 26.36 | 46.20 | 53.80 | 53.26 | 40.23 | 37.69 | 22.46 | 34.15 | 32.33 | 45.12 |
| Gelderland_Rn84 | 31.71 | 19.84 | 22.40 | 26.05 | 45.89 | 54.11 | 52.56 | 40.00 | 39.94 | 23.24 | 31.80 | 31.77 | 45.12 |
| Rn22 | 31.71 | 19.84 | 22.40 | 26.05 | 45.89 | 54.11 | 52.56 | 40.00 | 39.94 | 23.24 | 31.80 | 31.77 | 45.12 |
| GAW50/2021 | 31.47 | 19.92 | 23.33 | 25.27 | 45.19 | 54.81 | 53.72 | 40.00 | 40.55 | 22.02 | 35.47 | 28.43 | 41.86 |
| GAW30/2021 | 31.55 | 19.92 | 23.33 | 25.19 | 45.12 | 54.88 | 53.72 | 40.00 | 40.98 | 21.95 | 35.37 | 28.19 | 41.63 |
| GaomiRn47 | 31.71 | 19.46 | 22.95 | 25.89 | 45.35 | 54.65 | 53.02 | 40.00 | 39.94 | 21.41 | 34.56 | 30.77 | 43.02 |
| GaomiRn9 | 31.47 | 20.16 | 22.25 | 26.12 | 46.28 | 53.72 | 53.02 | 40.00 | 39.02 | 24.16 | 31.80 | 31.77 | 45.81 |
| GanyuRn137 | 31.55 | 19.46 | 23.02 | 25.97 | 45.43 | 54.57 | 53.02 | 40.00 | 39.33 | 21.41 | 34.86 | 31.10 | 43.26 |
| GanyuRn66 | 31.55 | 19.61 | 22.79 | 26.05 | 45.66 | 54.34 | 53.02 | 40.23 | 39.21 | 21.65 | 34.15 | 31.67 | 43.72 |
| GanyuMm187 | 31.55 | 19.53 | 22.87 | 26.05 | 45.58 | 54.42 | 53.02 | 40.00 | 39.33 | 21.71 | 34.25 | 31.44 | 43.72 |
| GAN36/2020 | 31.32 | 20.00 | 23.18 | 25.50 | 45.50 | 54.50 | 53.72 | 40.47 | 40.43 | 22.26 | 34.76 | 28.67 | 42.33 |
| GAN08/2018 | 31.63 | 20.00 | 23.10 | 25.27 | 45.27 | 54.73 | 53.72 | 40.00 | 41.16 | 22.32 | 34.56 | 28.43 | 42.09 |
| Fj372/2013 | 31.40 | 20.08 | 22.25 | 26.28 | 46.36 | 53.64 | 53.26 | 40.23 | 38.91 | 23.55 | 32.11 | 32.00 | 45.58 |
| FJ35 | 31.55 | 19.77 | 22.71 | 25.97 | 45.74 | 54.26 | 53.02 | 40.00 | 39.33 | 22.63 | 33.64 | 31.10 | 44.19 |
| FeixianRn1 | 31.55 | 20.08 | 22.33 | 26.05 | 46.12 | 53.88 | 52.79 | 40.00 | 39.02 | 23.85 | 32.11 | 31.77 | 45.58 |
| ERIZE-ST-DIZIER/Rn/FRA/2014/2014.00417 | 31.32 | 19.22 | 23.33 | 26.12 | 45.35 | 54.65 | 52.33 | 40.00 | 38.72 | 21.41 | 34.86 | 31.77 | 43.72 |
| ERIZE-ST-DIZIER/Hu/FRA/2014/2014.00479 | 31.32 | 19.22 | 23.33 | 26.12 | 45.35 | 54.65 | 52.33 | 40.00 | 38.72 | 21.41 | 34.86 | 31.77 | 43.72 |
| DPRK08 | 30.85 | 20.47 | 22.02 | 26.67 | 47.13 | 52.87 | 53.26 | 40.00 | 36.59 | 25.08 | 31.19 | 34.11 | 48.14 |
| DN2 | 31.55 | 19.84 | 22.56 | 26.05 | 45.89 | 54.11 | 52.56 | 40.00 | 39.33 | 23.55 | 32.42 | 31.44 | 45.12 |
| CVR/2019 | 31.47 | 19.77 | 22.71 | 26.05 | 45.81 | 54.19 | 52.79 | 40.00 | 38.91 | 22.56 | 33.23 | 32.11 | 44.65 |
| CSG5 | 31.32 | 19.77 | 22.71 | 26.20 | 45.97 | 54.03 | 52.33 | 40.00 | 38.41 | 23.24 | 32.72 | 32.44 | 45.58 |
| CixiRn169 | 31.40 | 19.69 | 22.87 | 26.05 | 45.74 | 54.26 | 52.56 | 40.00 | 38.72 | 22.63 | 33.64 | 31.77 | 44.65 |
| CixiRn76 | 31.47 | 19.53 | 22.79 | 26.20 | 45.74 | 54.26 | 52.56 | 40.00 | 38.60 | 23.24 | 33.64 | 31.33 | 44.65 |
| CixiRn21 | 31.32 | 20.39 | 22.33 | 25.97 | 46.36 | 53.64 | 52.56 | 39.77 | 38.34 | 25.30 | 31.40 | 31.99 | 46.74 |
| CixiRf56 | 31.55 | 20.16 | 22.71 | 25.58 | 45.74 | 54.26 | 52.56 | 40.00 | 39.63 | 23.78 | 32.62 | 30.43 | 44.65 |
| CixiRf23 | 31.63 | 19.61 | 22.87 | 25.89 | 45.50 | 54.50 | 53.26 | 40.00 | 39.51 | 21.71 | 34.56 | 30.67 | 43.26 |
| Cherwell | 31.55 | 19.69 | 22.79 | 25.97 | 45.66 | 54.34 | 52.79 | 40.00 | 39.21 | 22.26 | 33.54 | 31.77 | 44.19 |
| BjHD01 | 31.63 | 19.84 | 22.56 | 25.97 | 45.81 | 54.19 | 53.02 | 40.00 | 39.63 | 22.94 | 33.03 | 31.10 | 44.42 |
| AYN21/2018 | 30.85 | 20.23 | 22.87 | 26.05 | 46.28 | 53.72 | 53.95 | 40.00 | 38.11 | 22.94 | 33.94 | 31.77 | 44.88 |
| 201701593/SEOV/Colorado_US/Rat | 31.63 | 19.61 | 22.87 | 25.89 | 45.50 | 54.50 | 52.56 | 39.77 | 39.33 | 22.26 | 33.54 | 31.88 | 44.19 |
| 201701022/SEOV/Illinois_US/Rat | 31.55 | 19.69 | 22.79 | 25.97 | 45.66 | 54.34 | 52.79 | 39.77 | 39.33 | 22.56 | 33.23 | 31.88 | 44.42 |
| 201700048/SEOV/Illinois_US/Rat | 31.63 | 19.61 | 22.87 | 25.89 | 45.50 | 54.50 | 52.56 | 39.77 | 39.33 | 22.26 | 33.54 | 31.88 | 44.19 |
| 80-39 | 31.40 | 19.69 | 22.71 | 26.20 | 45.89 | 54.11 | 53.02 | 40.00 | 38.72 | 22.02 | 33.64 | 32.44 | 44.65 |
| HBT64/2014 | 31.55 | 20.08 | 22.25 | 26.12 | 46.20 | 53.80 | 53.02 | 40.00 | 39.33 | 23.85 | 31.80 | 31.77 | 45.58 |
| HBT63/2013 | 31.63 | 19.77 | 22.48 | 26.12 | 45.89 | 54.11 | 53.02 | 40.00 | 39.63 | 22.63 | 32.72 | 31.77 | 44.65 |
| HBT62/2014 | 31.55 | 19.84 | 22.56 | 26.05 | 45.89 | 54.11 | 53.02 | 39.77 | 39.33 | 23.24 | 32.72 | 31.44 | 44.88 |
| HBT61/2014 | 31.55 | 19.92 | 22.48 | 26.05 | 45.97 | 54.03 | 53.02 | 40.00 | 39.33 | 23.24 | 32.72 | 31.44 | 44.88 |
| HBT60/2013 | 31.63 | 19.77 | 22.48 | 26.12 | 45.89 | 54.11 | 53.02 | 40.00 | 39.63 | 22.63 | 32.72 | 31.77 | 44.65 |
| HBT52/2013 | 31.63 | 19.69 | 22.48 | 26.20 | 45.89 | 54.11 | 53.02 | 40.00 | 39.63 | 22.63 | 32.72 | 31.77 | 44.65 |
| HBT43/2012 | 31.55 | 19.77 | 22.56 | 26.12 | 45.89 | 54.11 | 53.02 | 40.00 | 39.33 | 22.63 | 33.03 | 31.77 | 44.65 |
| HBT41/2012 | 31.63 | 19.77 | 22.56 | 26.05 | 45.81 | 54.19 | 52.79 | 40.00 | 39.33 | 22.63 | 33.03 | 31.77 | 44.65 |
| HBT7/2016 | 31.40 | 19.92 | 22.40 | 26.28 | 46.20 | 53.80 | 53.02 | 40.00 | 38.72 | 23.24 | 32.42 | 32.44 | 45.58 |
| HBT6/2015 | 31.47 | 20.08 | 22.25 | 26.20 | 46.28 | 53.72 | 53.02 | 40.00 | 39.02 | 23.85 | 31.80 | 32.11 | 45.81 |
| HBT5/2016 | 31.47 | 19.77 | 22.56 | 26.20 | 45.97 | 54.03 | 53.02 | 40.00 | 39.02 | 22.63 | 33.03 | 32.11 | 44.88 |
| HBQ75/2000 | 31.63 | 19.61 | 22.79 | 25.97 | 45.58 | 54.42 | 53.02 | 40.00 | 39.63 | 22.02 | 33.94 | 31.10 | 43.72 |
| HBQ74/2000 | 31.63 | 19.92 | 22.40 | 26.05 | 45.97 | 54.03 | 53.02 | 40.00 | 39.63 | 23.24 | 32.42 | 31.44 | 44.88 |
| HBQ73/2000 | 31.32 | 20.08 | 22.48 | 26.12 | 46.20 | 53.80 | 53.02 | 40.00 | 38.41 | 23.85 | 32.72 | 31.77 | 45.58 |
| HBQ72/2000 | 31.47 | 20.08 | 22.33 | 26.12 | 46.20 | 53.80 | 53.02 | 40.00 | 39.02 | 23.85 | 32.11 | 31.77 | 45.58 |
| HBQ65/2009 | 31.63 | 19.77 | 22.48 | 26.12 | 45.89 | 54.11 | 53.02 | 40.00 | 39.63 | 22.63 | 32.72 | 31.77 | 44.65 |
| HBQ64/2022 | 31.47 | 20.00 | 22.25 | 26.28 | 46.28 | 53.72 | 53.02 | 40.00 | 39.02 | 23.55 | 31.80 | 32.44 | 45.81 |
| HBQ63/2021 | 31.63 | 20.00 | 22.33 | 26.05 | 46.05 | 53.95 | 53.02 | 40.00 | 39.63 | 23.55 | 32.11 | 31.44 | 45.12 |
| HBQ63/2001 | 31.47 | 20.08 | 22.33 | 26.12 | 46.20 | 53.80 | 53.02 | 40.00 | 39.02 | 23.85 | 32.11 | 31.77 | 45.58 |
| HBQ62/2001 | 31.55 | 19.92 | 22.48 | 26.05 | 45.97 | 54.03 | 53.02 | 40.00 | 39.33 | 23.24 | 32.72 | 31.44 | 44.88 |
| HBQ60/2021 | 31.47 | 20.16 | 22.25 | 26.12 | 46.28 | 53.72 | 53.02 | 40.00 | 39.02 | 24.16 | 31.80 | 31.77 | 45.81 |
| HBQ59/2021 | 31.71 | 20.08 | 22.25 | 25.97 | 46.05 | 53.95 | 53.02 | 40.00 | 39.94 | 23.85 | 31.80 | 31.10 | 45.12 |
| HBQ58/2021 | 31.71 | 20.08 | 22.25 | 25.97 | 46.05 | 53.95 | 53.02 | 40.00 | 39.94 | 23.85 | 31.80 | 31.10 | 45.12 |
| HBQ57/2021 | 31.47 | 20.16 | 22.25 | 26.12 | 46.28 | 53.72 | 53.02 | 40.00 | 39.02 | 24.16 | 31.80 | 31.77 | 45.81 |
| HBQ50/2021 | 31.55 | 20.00 | 22.33 | 26.12 | 46.12 | 53.88 | 53.02 | 40.00 | 39.33 | 23.55 | 32.11 | 31.77 | 45.35 |
| HBQ49/2004 | 31.55 | 19.92 | 22.40 | 26.12 | 46.05 | 53.95 | 53.02 | 40.00 | 39.33 | 23.24 | 32.42 | 31.77 | 45.12 |
| HBQ47/2021 | 31.32 | 20.08 | 22.33 | 26.28 | 46.36 | 53.64 | 53.02 | 40.00 | 38.41 | 23.85 | 32.11 | 32.44 | 46.05 |
| HBQ24/2011 | 31.55 | 19.92 | 22.48 | 26.05 | 45.97 | 54.03 | 53.02 | 40.00 | 39.33 | 23.24 | 32.72 | 31.44 | 44.88 |
| HBQ15/2018 | 31.55 | 20.08 | 22.25 | 26.12 | 46.20 | 53.80 | 53.02 | 40.00 | 39.33 | 23.85 | 31.80 | 31.77 | 45.58 |
| HBQ7/2012 | 31.47 | 19.84 | 22.64 | 26.05 | 45.89 | 54.11 | 53.02 | 40.00 | 39.02 | 22.94 | 33.33 | 31.44 | 44.65 |
| HBQ5/2015 | 31.63 | 20.08 | 22.25 | 26.05 | 46.12 | 53.88 | 53.02 | 40.00 | 39.63 | 23.85 | 31.80 | 31.44 | 45.35 |
| HBQ4/2012 | 31.55 | 19.92 | 22.48 | 26.05 | 45.97 | 54.03 | 53.02 | 40.00 | 39.33 | 23.24 | 32.72 | 31.44 | 44.88 |
| HBQ2/2015 | 31.71 | 19.84 | 22.48 | 25.97 | 45.81 | 54.19 | 53.02 | 40.00 | 39.94 | 22.94 | 32.72 | 31.10 | 44.42 |
| HBQ1/2015 | 31.63 | 19.92 | 22.40 | 26.05 | 45.97 | 54.03 | 53.02 | 40.00 | 39.63 | 23.24 | 32.42 | 31.44 | 44.88 |
| HBL131/2007 | 31.63 | 20.16 | 22.17 | 26.05 | 46.20 | 53.80 | 52.79 | 40.00 | 39.33 | 24.16 | 31.50 | 31.77 | 45.81 |
| HBL3/2005 | 31.32 | 19.69 | 22.95 | 26.05 | 45.74 | 54.26 | 53.02 | 40.23 | 38.60 | 22.02 | 34.56 | 31.33 | 43.95 |
| HBH51/2021 | 31.32 | 19.61 | 23.02 | 26.05 | 45.66 | 54.34 | 53.02 | 40.23 | 38.91 | 22.02 | 34.56 | 31.00 | 43.72 |
| HBCZ88/1999 | 31.40 | 19.69 | 22.95 | 25.97 | 45.66 | 54.34 | 53.02 | 40.23 | 38.91 | 22.02 | 34.56 | 31.00 | 43.72 |
| HBCD56/2002 | 31.55 | 20.16 | 22.25 | 26.05 | 46.20 | 53.80 | 53.26 | 40.00 | 39.63 | 24.16 | 31.80 | 31.00 | 45.35 |
| HBCD55/2021 | 31.55 | 19.92 | 22.40 | 26.12 | 46.05 | 53.95 | 53.02 | 40.00 | 39.33 | 23.24 | 32.42 | 31.77 | 45.12 |
| HBCD55/2002 | 31.32 | 19.38 | 23.10 | 26.20 | 45.58 | 54.42 | 52.33 | 40.00 | 38.41 | 21.71 | 34.25 | 32.44 | 44.42 |
| HBCD52/2002 | 31.55 | 20.16 | 22.25 | 26.05 | 46.20 | 53.80 | 53.26 | 40.00 | 39.63 | 24.16 | 31.80 | 31.00 | 45.35 |
| HBCD44/2021 | 31.47 | 20.00 | 22.40 | 26.12 | 46.12 | 53.88 | 53.02 | 40.00 | 39.02 | 23.55 | 32.42 | 31.77 | 45.35 |
| HBCD13/2019 | 31.55 | 20.08 | 22.25 | 26.12 | 46.20 | 53.80 | 53.02 | 40.00 | 39.33 | 23.85 | 31.80 | 31.77 | 45.58 |
| HBCD9/2017 | 31.55 | 20.08 | 22.25 | 26.12 | 46.20 | 53.80 | 53.02 | 40.00 | 39.33 | 23.85 | 31.80 | 31.77 | 45.58 |
| HBCD8/2017 | 31.55 | 20.08 | 22.25 | 26.12 | 46.20 | 53.80 | 53.02 | 40.00 | 39.33 | 23.85 | 31.80 | 31.77 | 45.58 |
| HBCD6/2017 | 31.55 | 20.08 | 22.25 | 26.12 | 46.20 | 53.80 | 53.02 | 40.00 | 39.33 | 23.85 | 31.80 | 31.77 | 45.58 |
| HBCD4/2017 | 31.71 | 20.00 | 22.25 | 26.05 | 46.05 | 53.95 | 53.02 | 40.00 | 39.94 | 23.55 | 31.80 | 31.44 | 45.12 |
| HBCD3/2017 | 31.55 | 20.08 | 22.25 | 26.12 | 46.20 | 53.80 | 53.02 | 40.00 | 39.33 | 23.85 | 31.80 | 31.77 | 45.58 |
| HBCD1/2017 | 31.55 | 20.08 | 22.25 | 26.12 | 46.20 | 53.80 | 53.02 | 40.00 | 39.33 | 23.85 | 31.80 | 31.77 | 45.58 |
| HBB36/2002 | 31.78 | 19.38 | 23.02 | 25.81 | 45.19 | 54.81 | 53.26 | 40.00 | 40.24 | 20.80 | 35.17 | 30.43 | 42.33 |
| HBB35/2002 | 31.63 | 19.53 | 22.87 | 25.97 | 45.50 | 54.50 | 53.26 | 40.00 | 39.63 | 21.41 | 34.56 | 31.10 | 43.26 |
| 93HBX12 | 31.24 | 20.08 | 22.48 | 26.20 | 46.28 | 53.72 | 53.26 | 40.00 | 39.53 | 21.91 | 34.88 | 31.67 | 45.58 |
| 93HBX11 | 31.32 | 20.08 | 22.48 | 26.12 | 46.20 | 53.80 | 53.26 | 40.00 | 39.77 | 21.91 | 34.88 | 31.44 | 45.35 |
| 93HBX10 | 31.63 | 20.08 | 22.25 | 26.05 | 46.12 | 53.88 | 53.02 | 40.00 | 40.70 | 22.37 | 33.95 | 30.98 | 45.35 |
| 93HBQ4 | 31.32 | 20.00 | 22.56 | 26.12 | 46.12 | 53.88 | 53.26 | 40.00 | 39.77 | 22.67 | 35.12 | 31.44 | 45.12 |
| 93HBQ3 | 31.24 | 20.08 | 22.48 | 26.20 | 46.28 | 53.72 | 53.26 | 40.00 | 39.53 | 22.91 | 34.88 | 31.67 | 45.58 |
| 93HBJ20 | 31.24 | 20.08 | 22.48 | 26.20 | 46.28 | 53.72 | 53.26 | 40.00 | 39.53 | 23.91 | 34.88 | 31.67 | 45.58 |
|  |  |  |  |  |  |  |  |  |  |  |  |  |  |
| Mean | 31.36 | 19.83 | 22.70 | 26.11 | 45.94 | 54. 06 | 52.97 | 40.02 | 38.72 | 22. 73 | 33.38 | 31.90 | 44.84 |
| Standard deviation | 0.28 | 0.25 | 0.32 | 0.22 | 0.32 | 0.32 | 0.35 | 0.17 | 0.91 | 0.87 | 1.08 | 0.91 | 0.90 |

Table S10. RSCU patterns of SEOV (based on isolation hosts) with H. sapiens and R. norvegicus.

| Amino acid | Codon | SEOV-H. sapiens | | | **H. sapiens** | **SEOV-R. norvegicus** | | | **R. norvegicus** |
| --- | --- | --- | --- | --- | --- | --- | --- | --- | --- |
|  |  | L | M | S |  | **L** | **M** | **S** |  |
| Phe | UUU | 1.46 | 1.55 | 1.18 | 0.93 | 1.49 | 1.48 | 1.12 | 0.83 |
| Phe | UUC | 0.54 | 0.45 | 0.82 | 1.07 | 0.51 | 0.52 | 0.88 | 1.17 |
| Leu | UUA | 1.53 | 1.54 | 0.99 | 0.46 | 1.66 | 1.37 | 0.87 | 0.36 |
| Leu | UUG | 1.36 | 1.09 | 1.00 | 0.77 | 1.20 | 1.28 | 0.88 | 0.76 |
| Leu | CUU | 1.29 | 0.99 | 1.34 | 0.79 | 1.41 | 1.08 | 1.40 | 0.75 |
| Leu | CUC | 0.41 | 1.06 | 0.69 | 1.17 | 0.33 | 0.94 | 0.68 | 1.22 |
| Leu | CUA | 0.77 | 0.80 | 0.54 | 0.43 | 0.86 | 0.83 | 0.40 | 0.45 |
| Leu | CUG | 0.64 | 0.53 | 1.43 | **2.37** | 0.54 | 0.50 | **1.77** | **2.46** |
| Ile | AUU | 1.46 | 1.59 | 1.12 | 1.08 | 1.43 | 1.54 | 0.98 | 0.98 |
| Ile | AUC | 0.77 | 0.69 | 1.03 | 1.41 | 0.73 | 0.71 | 1.16 | 1.57 |
| Ile | AUA | 0.78 | 0.72 | 0.85 | 0.51 | 0.84 | 0.75 | 0.87 | 0.45 |
| Val | GUU | 1.54 | **1.73** | 1.18 | 0.73 | 1.45 | **1.73** | 0.80 | 0.65 |
| Val | GUC | 0.33 | 0.93 | 1.02 | 0.95 | 0.45 | 0.93 | 1.09 | 1.02 |
| Val | GUA | 1.30 | 0.64 | 0.67 | 0.47 | 1.25 | 0.64 | 0.74 | 0.45 |
| Val | GUG | 0.84 | 0.71 | 1.14 | 1.85 | 0.84 | 0.70 | 1.37 | **1.88** |
| Ser | UCU | 1.41 | 1.15 | 0.85 | 1.13 | 1.48 | 1.13 | 0.83 | 1.12 |
| Ser | UCC | 0.45 | 0.37 | 0.27 | 1.31 | 0.42 | 0.36 | 0.14 | 1.35 |
| Ser | UCA | **2.04** | **2.38** | **2.81** | 0.90 | **2.01** | **2.40** | **2.89** | 0.83 |
| Ser | UCG | 0.18 | 0.07 | 0.13 | 0.33 | 0.21 | 0.06 | 0.22 | 0.33 |
| Ser | AGU | 1.36 | 1.11 | 1.08 | 0.90 | 1.34 | 1.36 | 0.97 | 0.90 |
| Ser | AGC | 0.56 | 0.92 | 0.86 | 1.44 | 0.55 | 0.69 | 0.94 | 1.46 |
| Pro | CCU | **1.81** | 1.53 | **1.80** | 1.15 | **1.76** | 1.52 | **1.81** | 1.20 |
| Pro | CCC | 0.40 | 0.58 | 0.50 | 1.29 | 0.28 | 0.56 | 0.54 | 1.25 |
| Pro | CCA | 1.63 | **1.67** | 1.41 | 1.11 | 1.72 | **1.73** | 1.35 | 1.12 |
| Pro | CCG | 0.16 | 0.22 | 0.30 | 0.45 | 0.23 | 0.19 | 0.30 | 0.43 |
| Thr | ACU | 1.26 | 1.18 | 0.90 | 0.99 | 1.16 | 1.22 | 0.78 | 0.96 |
| Thr | ACC | 0.16 | 0.32 | 0.23 | 1.42 | 0.26 | 0.29 | 0.20 | 1.46 |
| Thr | ACA | **2.43** | **2.25** | **2.72** | 1.14 | **2.40** | **2.30** | **2.88** | 1.13 |
| Thr | ACG | 0.14 | 0.24 | 0.15 | 0.46 | 0.18 | 0.18 | 0.14 | 0.46 |
| Ala | GCU | 1.47 | 0.94 | 1.10 | 1.06 | 1.49 | 0.93 | 1.12 | 1.14 |
| Ala | GCC | 0.47 | 1.01 | 0.76 | 1.60 | 0.32 | 1.03 | 0.73 | 1.57 |
| Ala | GCA | **1.98** | **1.99** | **2.07** | 0.91 | **2.15** | **1.98** | **2.10** | 0.90 |
| Ala | GCG | 0.08 | 0.06 | 0.08 | 0.42 | 0.05 | 0.07 | 0.05 | 0.40 |
| Tyr | UAU | **1.64** | 1.40 | 1.46 | 0.89 | 1.58 | 1.29 | 1.52 | 0.81 |
| Tyr | UAC | 0.36 | 0.60 | 0.54 | 1.11 | 0.42 | 0.71 | 0.48 | 1.19 |
| His | CAU | 1.43 | 1.42 | **1.65** | 0.84 | 1.47 | 1.49 | **1.68** | 0.78 |
| His | CAC | 0.57 | 0.58 | 0.35 | 1.16 | 0.53 | 0.51 | 0.32 | 1.22 |
| Gln | CAA | 1.15 | 1.10 | 0.94 | 0.53 | 1.11 | 1.06 | 0.89 | 0.49 |
| Gln | CAG | 0.85 | 0.90 | 1.06 | 1.47 | 0.89 | 0.94 | 1.11 | 1.51 |
| Asn | AAU | 1.39 | 1.17 | 0.99 | 0.94 | 1.22 | 1.27 | 0.84 | 0.82 |
| Asn | AAC | 0.61 | 0.83 | 1.01 | 1.06 | 0.78 | 0.73 | 1.16 | 1.18 |
| Lys | AAA | 1.25 | 1.36 | 0.78 | 0.87 | 1.18 | 1.31 | 0.57 | 0.76 |
| Lys | AAG | 0.75 | 0.64 | 1.22 | 1.13 | 0.82 | 0.69 | 1.43 | 1.24 |
| Asp | GAU | 1.53 | 1.35 | 1.20 | 0.93 | 1.53 | 1.41 | 1.15 | 0.86 |
| Asp | GAC | 0.47 | 0.66 | 0.80 | 1.07 | 0.47 | 0.59 | 0.85 | 1.14 |
| Glu | GAA | 1.17 | 1.37 | 1.19 | 0.84 | 1.11 | 1.36 | 1.17 | 0.79 |
| Glu | GAG | 0.83 | 0.63 | 0.81 | 1.16 | 0.89 | 0.64 | 0.83 | 1.21 |
| Cys | UGU | 1.33 | 1.36 | 1.30 | 0.91 | 1.27 | 1.30 | 1.26 | 0.91 |
| Cys | UGC | 0.67 | 0.64 | 0.70 | 1.09 | 0.73 | 0.70 | 0.74 | 1.09 |
| Arg | CGU | 0.46 | 0.18 | 0.39 | 0.48 | 0.42 | 0.16 | 0.47 | 0.54 |
| Arg | CGC | 0.12 | 0.00 | 0.58 | 1.10 | 0.19 | 0.02 | 0.81 | 1.06 |
| Arg | CGA | 0.64 | 0.84 | 0.36 | 0.65 | 0.60 | 0.80 | 0.23 | 0.73 |
| Arg | CGG | 0.58 | 0.48 | 0.48 | 1.21 | 0.54 | 0.52 | 0.46 | 1.18 |
| Arg | AGA | **1.96** | **2.60** | **2.29** | 1.29 | **1.92** | **2.70** | **2.12** | 1.21 |
| Arg | AGG | **2.23** | 1.90 | 1.90 | 1.27 | **2.33** | 1.80 | 1.91 | 1.28 |
| Gly | GGU | **1.61** | 1.56 | 1.01 | 0.65 | **1.81** | 1.59 | 0.78 | 0.70 |
| Gly | GGC | 0.74 | 0.81 | 0.43 | 1.35 | 0.45 | 0.81 | 0.30 | 1.34 |
| Gly | GGA | 0.90 | 0.79 | 0.95 | 1.00 | 0.96 | 0.67 | 1.09 | 1.02 |
| Gly | GGG | 0.76 | 0.84 | **1.60** | 1.00 | 0.78 | 0.93 | **1.83** | 0.95 |

The bold represents the over-represented codons (RSCU ≥1.6); the underline represents the most frequently used codons

Table S11. RSCU patterns of L segment of different clades.

| **Amino acid** | Codon | Overall | A | B | C | D | E | F | G |
| --- | --- | --- | --- | --- | --- | --- | --- | --- | --- |
| Phe | UUU | 1.49 | 1.48 | 1.48 | 1.43 | 1.55 | 1.52 | 1.49 | 1.47 |
| Phe | UUC | 0.51 | 0.52 | 0.52 | 0.57 | 0.45 | 0.48 | 0.51 | 0.53 |
| Leu | UUA | **1.66** | **1.68** | **1.66** | **1.60** | 1.58 | **1.61** | 1.55 | **2.02** |
| Leu | UUG | 1.2 | 1.18 | 1.29 | 1.20 | 1.23 | 1.20 | 1.33 | 1.20 |
| Leu | CUU | 1.41 | 1.45 | 1.39 | 1.36 | 1.39 | 1.30 | 1.28 | 1.41 |
| Leu | CUC | 0.33 | 0.30 | 0.37 | 0.35 | 0.30 | 0.40 | 0.40 | 0.36 |
| Leu | CUA | 0.86 | 0.87 | 0.76 | 0.85 | 0.84 | 0.87 | 0.80 | 0.66 |
| Leu | CUG | 0.54 | 0.52 | 0.53 | 0.64 | 0.67 | 0.62 | 0.63 | 0.36 |
| Ile | AUU | 1.43 | 1.43 | 1.42 | 1.45 | 1.40 | 1.42 | 1.44 | 1.59 |
| Ile | AUC | 0.73 | 0.73 | 0.69 | 0.81 | 0.78 | 0.70 | 0.78 | 0.67 |
| Ile | AUA | 0.84 | 0.84 | 0.88 | 0.75 | 0.81 | 0.88 | 0.78 | 0.74 |
| Val | GUU | 1.46 | 1.44 | 1.45 | 1.52 | 1.57 | 1.46 | 1.54 | 1.56 |
| Val | GUC | 0.45 | 0.47 | 0.47 | 0.42 | 0.38 | 0.47 | 0.30 | 0.47 |
| Val | GUA | 1.25 | 1.24 | 1.28 | 1.22 | 1.28 | 1.29 | 1.30 | 0.99 |
| Val | GUG | 0.84 | 0.86 | 0.80 | 0.84 | 0.77 | 0.78 | 0.85 | 0.98 |
| Ser | UCU | 1.47 | 1.50 | 1.45 | 1.46 | 1.42 | 1.36 | 1.43 | 1.50 |
| Ser | UCC | 0.42 | 0.39 | 0.52 | 0.48 | 0.48 | 0.52 | 0.45 | 0.41 |
| Ser | UCA | **2.01** | **1.99** | **2.01** | **1.94** | **2.07** | **2.06** | **2.04** | **1.96** |
| Ser | UCG | 0.21 | 0.23 | 0.15 | 0.26 | 0.17 | 0.19 | 0.16 | 0.20 |
| Ser | AGU | 1.34 | 1.36 | 1.30 | 1.21 | 1.20 | 1.28 | 1.34 | 1.37 |
| Ser | AGC | 0.55 | 0.53 | 0.57 | 0.66 | 0.67 | 0.59 | 0.57 | 0.57 |
| Pro | CCU | **1.77** | **1.76** | **1.64** | **1.76** | **1.75** | **1.78** | **1.82** | **1.83** |
| Pro | CCC | 0.28 | 0.27 | 0.48 | 0.27 | 0.27 | 0.27 | 0.38 | 0.27 |
| Pro | CCA | **1.72** | **1.73** | **1.65** | **1.71** | **1.77** | **1.77** | **1.64** | **1.75** |
| Pro | CCG | 0.23 | 0.25 | 0.23 | 0.27 | 0.21 | 0.19 | 0.16 | 0.16 |
| Thr | ACU | 1.17 | 1.15 | 1.05 | 1.14 | 1.23 | 1.19 | 1.28 | 1.31 |
| Thr | ACC | 0.26 | 0.28 | 0.30 | 0.25 | 0.24 | 0.21 | 0.16 | 0.11 |
| Thr | ACA | **2.4** | **2.38** | **2.58** | **2.46** | **2.43** | **2.41** | **2.40** | **2.44** |
| Thr | ACG | 0.18 | 0.19 | 0.07 | 0.14 | 0.11 | 0.19 | 0.17 | 0.15 |
| Ala | GCU | 1.49 | 1.52 | 1.40 | 1.40 | 1.35 | 1.37 | 1.49 | 1.56 |
| Ala | GCC | 0.32 | 0.28 | 0.45 | 0.35 | 0.54 | 0.36 | 0.45 | 0.47 |
| Ala | GCA | **2.14** | **2.16** | **2.10** | **2.06** | **2.02** | **2.23** | **1.98** | **1.91** |
| Ala | GCG | 0.05 | 0.05 | 0.05 | 0.19 | 0.09 | 0.04 | 0.08 | 0.06 |
| Tyr | UAU | 1.58 | 1.56 | **1.66** | **1.64** | 1.57 | **1.64** | **1.63** | **1.65** |
| Tyr | UAC | 0.42 | 0.44 | 0.34 | 0.36 | 0.43 | 0.36 | 0.37 | 0.35 |
| His | CAU | 1.47 | 1.45 | 1.53 | 1.56 | 1.49 | 1.57 | 1.45 | 1.33 |
| His | CAC | 0.53 | 0.55 | 0.47 | 0.44 | 0.51 | 0.43 | 0.55 | 0.67 |
| Gln | CAA | 1.11 | 1.09 | 1.15 | 1.05 | 1.19 | 1.15 | 1.15 | 1.19 |
| Gln | CAG | 0.89 | 0.91 | 0.85 | 0.95 | 0.81 | 0.85 | 0.85 | 0.81 |
| Asn | AAU | 1.22 | 1.18 | 1.36 | 1.30 | 1.32 | 1.25 | 1.38 | 1.41 |
| Asn | AAC | 0.78 | 0.82 | 0.64 | 0.70 | 0.68 | 0.75 | 0.62 | 0.59 |
| Lys | AAA | 1.18 | 1.18 | 1.13 | 1.13 | 1.23 | 1.18 | 1.24 | 1.12 |
| Lys | AAG | 0.82 | 0.82 | 0.87 | 0.87 | 0.78 | 0.82 | 0.76 | 0.88 |
| Asp | GAU | 1.52 | 1.54 | 1.53 | 1.48 | 1.47 | 1.49 | 1.52 | 1.48 |
| Asp | GAC | 0.48 | 0.46 | 0.47 | 0.52 | 0.53 | 0.51 | 0.48 | 0.52 |
| Glu | GAA | 1.11 | 1.10 | 1.09 | 1.07 | 1.13 | 1.13 | 1.16 | 1.14 |
| Glu | GAG | 0.89 | 0.90 | 0.91 | 0.94 | 0.88 | 0.87 | 0.84 | 0.86 |
| Cys | UGU | 1.27 | 1.23 | 1.26 | 1.33 | 1.48 | 1.34 | 1.33 | 1.48 |
| Cys | UGC | 0.73 | 0.77 | 0.74 | 0.67 | 0.52 | 0.66 | 0.67 | 0.52 |
| Arg | CGU | 0.43 | 0.42 | 0.42 | 0.36 | 0.48 | 0.41 | 0.46 | 0.48 |
| Arg | CGC | 0.19 | 0.18 | 0.18 | 0.24 | 0.12 | 0.24 | 0.13 | 0.18 |
| Arg | CGA | 0.6 | 0.60 | 0.51 | 0.60 | 0.59 | 0.60 | 0.61 | 0.57 |
| Arg | CGG | 0.54 | 0.54 | 0.73 | 0.54 | 0.67 | 0.50 | 0.58 | 0.33 |
| Arg | AGA | **1.93** | **1.86** | **2.18** | **1.92** | **2.07** | **2.04** | **1.99** | **2.64** |
| Arg | AGG | **2.32** | **2.40** | **1.98** | **2.34** | **2.07** | **2.21** | **2.22** | **1.80** |
| Gly | GGU | **1.81** | **1.82** | **1.87** | **1.86** | **1.95** | **1.77** | **1.64** | **1.94** |
| Gly | GGC | 0.46 | 0.44 | 0.41 | 0.44 | 0.48 | 0.51 | 0.61 | 0.32 |
| Gly | GGA | 0.96 | 0.96 | 0.95 | 0.87 | 0.97 | 0.97 | 0.94 | 0.99 |
| Gly | GGG | 0.77 | 0.78 | 0.83 | 0.75 | 0.73 | 0.77 | 0.76 | 0.75 |

The bold represents the over-represented codons (RSCU ≥1.6); the underline represents the most frequently used codons

Table S12. RSCU patterns of M segment of different clades.

| **Amino acid** | Codon | Overall | A | B | C | D | E | F | G |
| --- | --- | --- | --- | --- | --- | --- | --- | --- | --- |
| Phe | UUU | 1.48 | 1.44 | 1.52 | 1.59 | 1.54 | 1.53 | 1.54 | 1.40 |
| Phe | UUC | 0.52 | 0.56 | 0.48 | 0.41 | 0.46 | 0.47 | 0.46 | 0.60 |
| Leu | UUA | 1.39 | 1.23 | **1.63** | 1.41 | **1.63** | 1.41 | 1.47 | **1.68** |
| Leu | UUG | 1.27 | 1.38 | 1.15 | 1.39 | 1.23 | 1.16 | 0.98 | 0.89 |
| Leu | CUU | 1.09 | 1.11 | 1.08 | 0.98 | 1.11 | 0.94 | 1.01 | 1.26 |
| Leu | CUC | 0.93 | 0.94 | 0.92 | 1.04 | 0.83 | 1.06 | 1.06 | 0.52 |
| Leu | CUA | 0.83 | 0.87 | 0.76 | 0.77 | 0.71 | 0.82 | 0.91 | 0.81 |
| Leu | CUG | 0.50 | 0.47 | 0.46 | 0.42 | 0.49 | 0.61 | 0.57 | 0.83 |
| Ile | AUU | 1.54 | 1.54 | 1.57 | 1.51 | 1.56 | 1.57 | 1.54 | 1.39 |
| Ile | AUC | 0.71 | 0.71 | 0.62 | 0.73 | 0.71 | 0.65 | 0.73 | 0.88 |
| Ile | AUA | 0.75 | 0.75 | 0.81 | 0.76 | 0.73 | 0.78 | 0.73 | 0.74 |
| Val | GUU | **1.72** | **1.73** | **1.63** | **1.71** | **1.73** | **1.61** | **1.90** | **1.60** |
| Val | GUC | 0.94 | 0.91 | 0.98 | 0.81 | 0.99 | 1.07 | 0.77 | 1.00 |
| Val | GUA | 0.64 | 0.63 | 0.67 | **0.72** | 0.65 | 0.58 | **0.62** | 0.65 |
| Val | GUG | 0.71 | 0.73 | 0.72 | 0.76 | 0.63 | 0.74 | 0.71 | 0.75 |
| Ser | UCU | 1.13 | 1.19 | 0.99 | 0.94 | 1.14 | 0.95 | 1.21 | 0.89 |
| Ser | UCC | 0.36 | 0.30 | 0.49 | 0.40 | 0.39 | 0.53 | 0.33 | 0.48 |
| Ser | UCA | **2.40** | **2.41** | **2.39** | **2.45** | **2.39** | **2.36** | **2.36** | **2.51** |
| Ser | UCG | 0.06 | 0.06 | 0.07 | 0.11 | 0.01 | 0.10 | 0.06 | 0.14 |
| Ser | AGU | 1.35 | 1.41 | 1.22 | 1.35 | 1.37 | 1.34 | 1.03 | 1.37 |
| Ser | AGC | 0.70 | 0.64 | 0.83 | 0.75 | 0.70 | 0.72 | 1.01 | 0.61 |
| Pro | CCU | 1.52 | 1.53 | 1.41 | 1.48 | 1.52 | 1.60 | 1.49 | 1.49 |
| Pro | CCC | 0.56 | 0.54 | 0.61 | 0.60 | 0.62 | 0.53 | 0.61 | 0.42 |
| Pro | CCA | **1.72** | **1.75** | **1.67** | **1.68** | **1.74** | **1.65** | **1.63** | **1.87** |
| Pro | CCG | 0.20 | 0.19 | 0.31 | 0.24 | 0.13 | 0.22 | 0.28 | 0.22 |
| Thr | ACU | 1.22 | 1.24 | 1.32 | 1.30 | 1.20 | 1.17 | 1.16 | 1.08 |
| Thr | ACC | 0.30 | 0.27 | 0.27 | 0.27 | 0.30 | 0.36 | 0.35 | 0.45 |
| Thr | ACA | **2.30** | **2.29** | **2.33** | **2.36** | **2.28** | **2.32** | **2.27** | **2.40** |
| Thr | ACG | 0.18 | 0.19 | 0.07 | 0.07 | 0.22 | 0.15 | 0.22 | 0.07 |
| Ala | GCU | 0.94 | 0.91 | 1.09 | 1.07 | 0.84 | 0.95 | 1.01 | 1.26 |
| Ala | GCC | 1.02 | 1.05 | 0.98 | 1.01 | 1.05 | 1.02 | 0.96 | 0.57 |
| Ala | GCA | **1.97** | **1.98** | **1.83** | **1.90** | **1.98** | **2.03** | **1.98** | **2.09** |
| Ala | GCG | 0.07 | 0.06 | 0.10 | 0.01 | 0.13 | 0.01 | 0.06 | 0.08 |
| Tyr | UAU | 1.29 | 1.24 | 1.32 | 1.26 | 1.35 | 1.25 | 1.48 | 1.30 |
| Tyr | UAC | 0.71 | 0.76 | 0.68 | 0.74 | 0.65 | 0.75 | 0.52 | 0.70 |
| His | CAU | 1.49 | 1.54 | 1.53 | 1.39 | 1.39 | 1.43 | 1.43 | 1.43 |
| His | CAC | 0.51 | 0.46 | 0.47 | 0.61 | 0.61 | 0.57 | 0.57 | 0.57 |
| Gln | CAA | 1.06 | 1.02 | 1.08 | 0.99 | 1.15 | 1.03 | 1.10 | 1.18 |
| Gln | CAG | 0.94 | 0.98 | 0.92 | 1.01 | 0.85 | 0.97 | 0.90 | 0.82 |
| Asn | AAU | 1.27 | 1.28 | 1.16 | 1.34 | 1.30 | 1.29 | 1.13 | 1.32 |
| Asn | AAC | 0.73 | 0.72 | 0.84 | 0.66 | 0.70 | 0.71 | 0.87 | 0.68 |
| Lys | AAA | 1.31 | 1.28 | 1.39 | 1.37 | 1.31 | 1.32 | 1.42 | 1.17 |
| Lys | AAG | 0.69 | 0.72 | 0.61 | 0.63 | 0.69 | 0.68 | 0.58 | 0.83 |
| Asp | GAU | 1.41 | 1.43 | 1.41 | 1.40 | 1.36 | 1.34 | 1.42 | 1.46 |
| Asp | GAC | 0.59 | 0.57 | 0.59 | 0.60 | 0.64 | 0.66 | 0.58 | 0.54 |
| Glu | GAA | 1.36 | 1.37 | 1.31 | 1.32 | 1.33 | 1.36 | 1.39 | 1.34 |
| Glu | GAG | 0.64 | 0.63 | 0.69 | 0.68 | 0.67 | 0.64 | 0.61 | 0.66 |
| Cys | UGU | 1.31 | 1.28 | 1.45 | 1.36 | 1.29 | 1.30 | 1.42 | 1.26 |
| Cys | UGC | 0.69 | 0.72 | 0.55 | 0.64 | 0.71 | 0.70 | 0.58 | 0.74 |
| Arg | CGU | 0.16 | 0.16 | 0.35 | 0.18 | 0.08 | 0.19 | 0.16 | 0.06 |
| Arg | CGC | 0.02 | 0.02 | 0.01 | 0.00 | 0.00 | 0.02 | 0.02 | 0.18 |
| Arg | CGA | 0.80 | 0.83 | 0.86 | 0.81 | 0.77 | 0.70 | 0.86 | 0.57 |
| Arg | CGG | 0.51 | 0.47 | 0.42 | 0.41 | 0.74 | 0.50 | 0.46 | 0.36 |
| Arg | AGA | **2.70** | **2.87** | **2.69** | **2.57** | **2.30** | **2.79** | **2.49** | **2.59** |
| Arg | AGG | **1.81** | **1.65** | **1.67** | **2.03** | **2.12** | **1.79** | **2.01** | **2.24** |
| Gly | GGU | **1.60** | 1.59 | **1.69** | **1.61** | 1.52 | 1.59 | **1.60** | **1.84** |
| Gly | GGC | 0.81 | 0.82 | 0.76 | 0.77 | 0.86 | 0.80 | 0.73 | 0.70 |
| Gly | GGA | 0.68 | 0.64 | 0.76 | 0.66 | 0.67 | 0.66 | 0.85 | 0.79 |
| Gly | GGG | 0.92 | 0.95 | 0.79 | 0.95 | 0.95 | 0.95 | 0.82 | 0.67 |

The bold represents the over-represented codons (RSCU ≥1.6); the underline represents the most frequently used codons

Table S13. RSCU patterns of S segment of different clades.

| **Amino acid** | Codon | **Overall** | A | **B** | C | **D** | E | **F** | G |
| --- | --- | --- | --- | --- | --- | --- | --- | --- | --- |
| Phe | UUU | 1.11 | 1.14 | 1.08 | 1.06 | 1.10 | 1.07 | 1.09 | 1.12 |
| Phe | UUC | 0.89 | 0.86 | 0.92 | 0.95 | 0.90 | 0.93 | 0.91 | 0.88 |
| Leu | UUA | 0.86 | 0.87 | 0.90 | 0.86 | 0.94 | 0.84 | 0.73 | 0.70 |
| Leu | UUG | 0.88 | 0.85 | 1.02 | 1.13 | 0.77 | 0.73 | 1.10 | 1.07 |
| Leu | CUU | 1.41 | 1.37 | 1.56 | 1.32 | 1.38 | 1.37 | 1.54 | 1.57 |
| Leu | CUC | 0.68 | 0.70 | 0.51 | 0.73 | 0.69 | 0.71 | 0.54 | 0.78 |
| Leu | CUA | 0.40 | 0.34 | 0.23 | 0.37 | 0.54 | 0.71 | 0.36 | 0.51 |
| Leu | CUG | **1.78** | **1.87** | **1.78** | 1.59 | **1.69** | **1.65** | **1.73** | **1.37** |
| Ile | AUU | 0.98 | 0.97 | 0.99 | 0.98 | 1.00 | 0.91 | 0.95 | 1.11 |
| Ile | AUC | 1.15 | 1.20 | 1.12 | 1.09 | 1.07 | 1.15 | 1.08 | 1.02 |
| Ile | AUA | 0.87 | 0.82 | 0.89 | 0.93 | 0.93 | 0.94 | 0.97 | 0.87 |
| Val | GUU | 0.81 | 0.78 | 0.71 | 0.75 | 0.76 | 0.88 | 0.93 | 1.21 |
| Val | GUC | 1.09 | 1.10 | 1.20 | 1.14 | 1.23 | 1.02 | 0.99 | 0.70 |
| Val | GUA | 0.73 | 0.73 | 0.74 | 0.77 | 0.62 | 0.72 | 0.83 | 0.90 |
| Val | GUG | 1.37 | 1.40 | 1.35 | 1.34 | 1.39 | 1.38 | 1.26 | 1.19 |
| Ser | UCU | 0.83 | 0.89 | 0.93 | 0.62 | 0.79 | 0.74 | 0.64 | 0.61 |
| Ser | UCC | 0.15 | 0.09 | 0.00 | 0.38 | 0.20 | 0.19 | 0.28 | 0.52 |
| Ser | UCA | **2.89** | **2.87** | **2.99** | **2.93** | **2.75** | **2.90** | **3.22** | **2.89** |
| Ser | UCG | 0.22 | 0.21 | 0.19 | 0.22 | 0.36 | 0.30 | 0.00 | 0.14 |
| Ser | AGU | 0.98 | 0.99 | 1.12 | 0.94 | 0.93 | 1.04 | 0.72 | 0.89 |
| Ser | AGC | 0.93 | 0.95 | 0.77 | 0.91 | 0.98 | 0.83 | 1.13 | 0.95 |
| Pro | CCU | **1.81** | **1.82** | **1.80** | **1.80** | **1.72** | **1.98** | **1.80** | **1.71** |
| Pro | CCC | 0.54 | 0.58 | 0.58 | 0.54 | 0.54 | 0.26 | 0.60 | 0.44 |
| Pro | CCA | 1.34 | 1.34 | 1.22 | 1.37 | 1.34 | 1.34 | 1.18 | 1.73 |
| Pro | CCG | 0.31 | 0.26 | 0.40 | 0.29 | 0.40 | 0.42 | 0.42 | 0.13 |
| Thr | ACU | 0.79 | 0.67 | 0.68 | 0.84 | 0.95 | 0.96 | 0.84 | 1.46 |
| Thr | ACC | 0.21 | 0.21 | 0.37 | 0.21 | 0.21 | 0.09 | 0.21 | 0.00 |
| Thr | ACA | **2.88** | **2.92** | **2.95** | **2.92** | **2.83** | **2.94** | **2.93** | **2.27** |
| Thr | ACG | 0.13 | 0.20 | 0.00 | 0.03 | 0.01 | 0.01 | 0.02 | 0.27 |
| Ala | GCU | 1.12 | 1.09 | 1.11 | 1.10 | 1.16 | 1.25 | 1.31 | 1.05 |
| Ala | GCC | 0.73 | 0.75 | 0.78 | 0.77 | 0.70 | 0.65 | 0.55 | 0.81 |
| Ala | GCA | **2.09** | **2.12** | **2.02** | **1.99** | **2.04** | **2.07** | **2.11** | **2.14** |
| Ala | GCG | 0.06 | 0.05 | 0.08 | 0.14 | 0.10 | 0.02 | 0.03 | 0.01 |
| Tyr | UAU | 1.53 | 1.56 | **1.79** | 1.51 | 1.58 | 1.37 | 1.33 | 1.03 |
| Tyr | UAC | 0.47 | 0.44 | 0.21 | 0.49 | 0.42 | 0.63 | 0.67 | 0.97 |
| His | CAU | **1.68** | **1.69** | **1.71** | **1.80** | **1.73** | **1.71** | **1.74** | 1.14 |
| His | CAC | 0.32 | 0.31 | 0.29 | 0.20 | 0.27 | 0.29 | 0.26 | 0.86 |
| Gln | CAA | 0.89 | 0.88 | 0.87 | 0.82 | 0.87 | 0.97 | 0.82 | 1.11 |
| Gln | CAG | 1.11 | 1.12 | 1.13 | 1.18 | 1.13 | 1.03 | 1.18 | 0.89 |
| Asn | AAU | 0.85 | 0.80 | 0.98 | 0.77 | 0.93 | 0.93 | 0.94 | 0.76 |
| Asn | AAC | 1.15 | 1.20 | 1.02 | 1.23 | 1.07 | 1.07 | 1.06 | 1.24 |
| Lys | AAA | 0.57 | 0.57 | 0.55 | 0.50 | 0.63 | 0.49 | 0.64 | 0.61 |
| Lys | AAG | 1.43 | 1.43 | 1.45 | 1.50 | 1.37 | 1.51 | 1.36 | 1.39 |
| Asp | GAU | 1.16 | 1.10 | 1.18 | 1.18 | 1.18 | 1.15 | 1.21 | 1.52 |
| Asp | GAC | 0.84 | 0.90 | 0.82 | 0.82 | 0.82 | 0.85 | 0.79 | 0.48 |
| Glu | GAA | 1.16 | 1.17 | 1.12 | 1.15 | 1.24 | 1.06 | 1.19 | 1.02 |
| Glu | GAG | 0.84 | 0.83 | 0.88 | 0.85 | 0.76 | 0.94 | 0.81 | 0.98 |
| Cys | UGU | 1.26 | 1.20 | 1.20 | 1.49 | 1.46 | 1.31 | 1.13 | 1.40 |
| Cys | UGC | 0.74 | 0.80 | 0.80 | 0.51 | 0.54 | 0.69 | 0.87 | 0.60 |
| Arg | CGU | 0.48 | 0.47 | 0.51 | 0.52 | 0.53 | 0.43 | 0.43 | 0.50 |
| Arg | CGC | 0.81 | 0.82 | 0.81 | 0.77 | 0.76 | 0.86 | 0.86 | 0.73 |
| Arg | CGA | 0.23 | 0.22 | 0.22 | 0.18 | 0.22 | 0.23 | 0.09 | 0.62 |
| Arg | CGG | 0.47 | 0.44 | 0.42 | 0.46 | 0.43 | 0.43 | 0.55 | 0.92 |
| Arg | AGA | **2.11** | **2.20** | **1.90** | **2.14** | **2.10** | **2.11** | **2.07** | **1.65** |
| Arg | AGG | **1.90** | **1.86** | **2.15** | **1.93** | **1.96** | **1.94** | **2.00** | 1.58 |
| Gly | GGU | 0.77 | 0.80 | 0.59 | 0.75 | 0.81 | 0.73 | 0.75 | 0.79 |
| Gly | GGC | 0.31 | 0.30 | 0.43 | 0.27 | 0.29 | 0.29 | 0.23 | 0.42 |
| Gly | GGA | 1.08 | 1.19 | 1.01 | 1.13 | 0.89 | 0.96 | 1.06 | 0.81 |
| Gly | GGG | **1.84** | **1.72** | **1.97** | **1.84** | **2.02** | **2.02** | **1.96** | **1.98** |

The bold represents the over-represented codons (RSCU ≥1.6); the underline represents the most frequently used codons

Table S14. The mean ENC values of different clades.

| **Segment** | **Overall** | **A** | **B** | **C** | **D** | **E** | **F** | **G** |
| --- | --- | --- | --- | --- | --- | --- | --- | --- |
| L | 47.02 | 47.10 | 46.93 | 47.07 | 46.91 | 46.89 | 47.17 | 45.61 |
| M | 47.98 | 47.84 | 48.30 | 47.85 | 48.32 | 48.25 | 48.06 | 46.75 |
| S | 49.19 | 49.20 | 48.25 | 48.69 | 49.45 | 49.00 | 48.51 | 51.35 |

Table S15. CAI values of different clades referenced to H. sapiens synonymous codon usage patterns.

| **Segment** | **Overall** | **A** | **B** | **C** | **D** | **E** | **F** | **G** |
| --- | --- | --- | --- | --- | --- | --- | --- | --- |
| L | 0.710±0.002 | 0.710±0.002 | 0.713±0.002 | 0.713±0.000 | 0.710±0.001 | 0.709±0.001 | 0.714±0.002 | 0.714±0.001 |
| M | 0.726±0.003 | 0.726±0.002 | 0.723±0.001 | 0.729±0.003 | 0.727±0.002 | 0.728±0.002 | 0.722±0.003 | 0.723±0.005 |
| S | 0.749±0.006 | 0.752±0.004 | 0.752±0.002 | 0.751±0.004 | 0.742±0.004 | 0.741±0.006 | 0.751±0.004 | 0.738±0.006 |

Table S16. CAI values of different clades referenced to R. norvegicus synonymous codon usage patterns.

| **Segment** | **Overall** | **A** | **B** | **C** | **D** | **E** | **F** | **G** |
| --- | --- | --- | --- | --- | --- | --- | --- | --- |
| L | 0.662±0.002 | 0.662±0.002 | 0.664±0.002 | 0.666±0.000 | 0.661±0.001 | 0.661±0.001 | 0.665±0.002 | 0.664±0.001 |
| M | 0.681±0.003 | 0.682±0.002 | 0.678±0.002 | 0.683±0.000 | 0.681±0.001 | 0.684±0.001 | 0.676±0.004 | 0.680±0.006 |
| S | 0.715±0.007 | 0.719±0.004 | 0.718±0.002 | 0.717±0.004 | 0.708±0.004 | 0.708±0.006 | 0.716±0.004 | 0.704±0.006 |

Table S17. RCDI values of different clades referenced to H. sapiens synonymous codon usage patterns.

| **Segment** | **Overall** | **A** | **B** | **C** | **D** | **E** | **F** | **G** |
| --- | --- | --- | --- | --- | --- | --- | --- | --- |
| L | 1.454±0.012 | 1.453±0.009 | 1.461±0.004 | 1.424±0.000 | 1.457±0.004 | 1.458±0.011 | 1.441±0.006 | 1.406±0.009 |
| M | 1.384±0.022 | 1.377±0.010 | 1.401±0.011 | 1.390±0.006 | 1.390±0.010 | 1.369±0.011 | 1.398±0.023 | 1.430±0.076 |
| S | 1.310±0.020 | 1.325±0.038 | 1.310±0.012 | 1.307±0.017 | 1.332±0.010 | 1.332±0.015 | 1.342±0.010 | 1.331±0.026 |

Table S18. RCDI values of different clades referenced to R. norvegicus synonymous codon usage patterns.

| **Segment** | **Overall** | **A** | **B** | **C** | **D** | **E** | **F** | **G** |
| --- | --- | --- | --- | --- | --- | --- | --- | --- |
| L | 1.536±0.014 | 1.534±0.010 | 1.546±0.004 | 1.502±0.000 | 1.540±0.005 | 1.541±0.012 | 1.524±0.007 | 1.595±0.011 |
| M | 1.454±0.026 | 1.442±0.012 | 1.477±0.012 | 1.462±0.011 | 1.467±0.013 | 1.438±0.013 | 1.473±0.026 | 1.501±0.083 |
| S | 1.345±0.022 | 1.333±0.015 | 1.364±0.012 | 1.344±0.021 | 1.346±0.012 | 1.365±0.017 | 1.381±0.011 | 1.370±0.029 |
