# Supplementary figures and images for: Genetic and codon usage analyses reveal the evolution of the seoul virus
Source: Front Genet. 2025 Jun 12;16:1544577. doi: 10.3389/fgene.2025.1544577 (PMC12198216; doi:10.3389/fgene.2025.1544577)

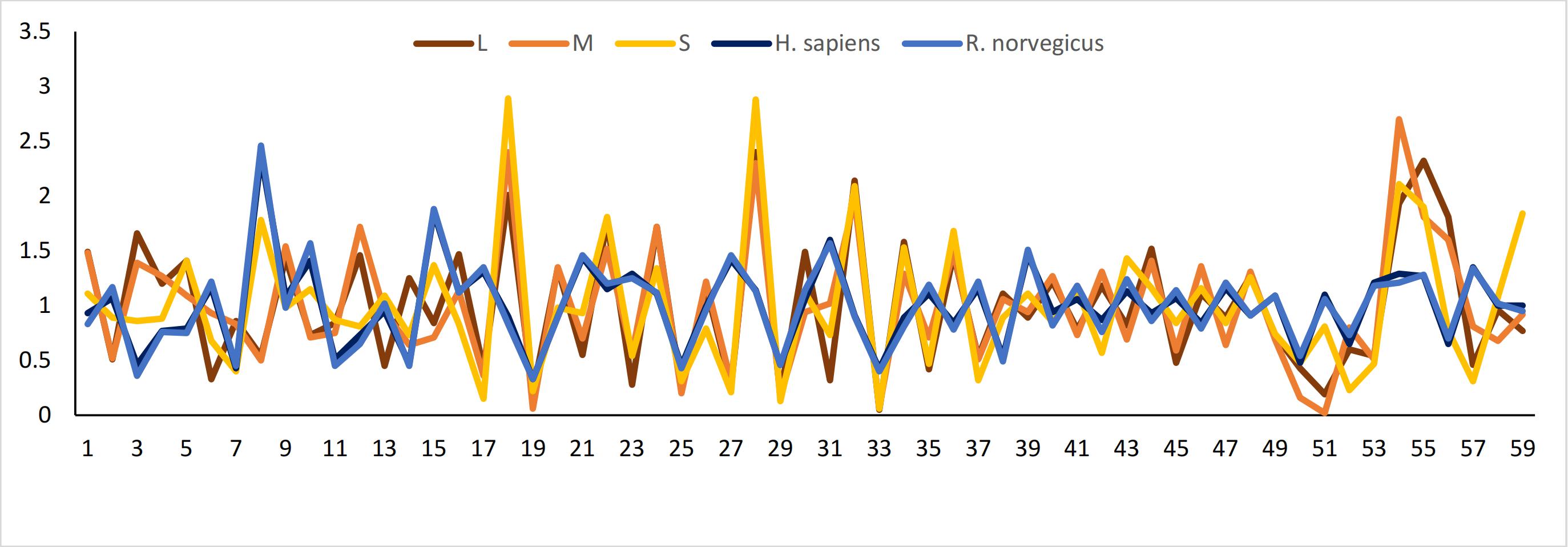

Supplement: Supplementary file 2 [file Image1.jpeg]

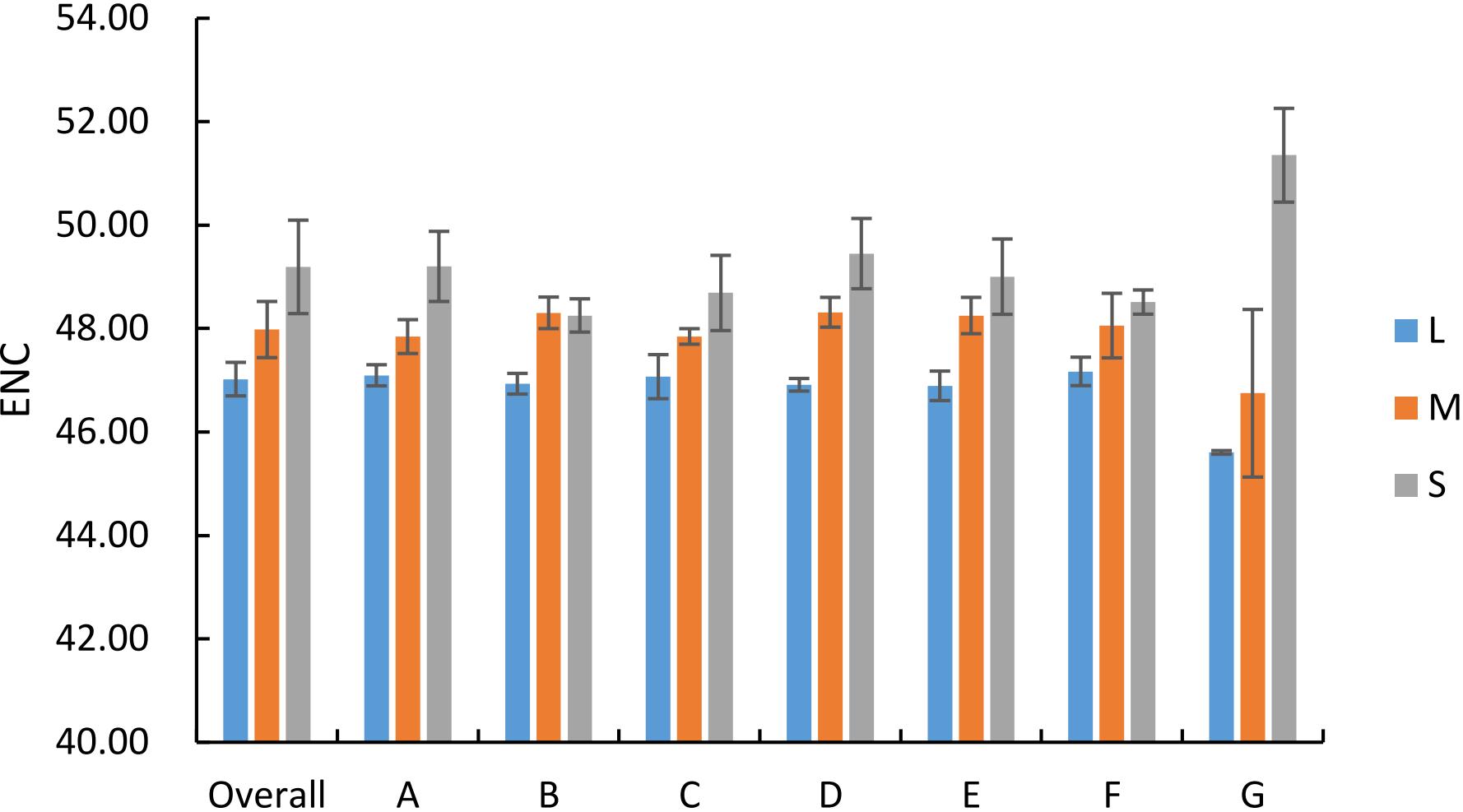

Supplement: Supplementary file 3 [file Image2.jpeg]
